# Design of mismatch closure for enhanced specificity in DNA strand displacement reactions

**DOI:** 10.1093/nar/gkaf660

**Published:** 2025-07-12

**Authors:** Hongyan Yu, Xiaole Han, Li Zhang, Na Yin, Li Wang, Ke Lv, Yongchang Wu, Dan Bai, Weitao Wang, Ying Huang, Xingping Hu, Zhi Weng, Chenlu Zhang, Gang Yang, Tingmei Chen, Guoming Xie

**Affiliations:** Key Laboratory of Clinical Laboratory Diagnostics (Chinese Ministry of Education), College of Laboratory Medicine, Chongqing Medical University, Chongqing 400016, PR China; Key Laboratory of Clinical Laboratory Diagnostics (Chinese Ministry of Education), College of Laboratory Medicine, Chongqing Medical University, Chongqing 400016, PR China; Key Laboratory of Clinical Laboratory Diagnostics (Chinese Ministry of Education), College of Laboratory Medicine, Chongqing Medical University, Chongqing 400016, PR China; Key Laboratory of Clinical Laboratory Diagnostics (Chinese Ministry of Education), College of Laboratory Medicine, Chongqing Medical University, Chongqing 400016, PR China; The Center for Clinical Molecular Medical Detection, Biobank Center, The First Affiliated Hospital of Chongqing Medical University, Chongqing 400016, PR China; Department of Neurosurgery, The First Affiliated Hospital of Chongqing Medical University, Chongqing 400016, PR China; Key Laboratory of Clinical Laboratory Diagnostics (Chinese Ministry of Education), College of Laboratory Medicine, Chongqing Medical University, Chongqing 400016, PR China; Key Laboratory of Clinical Laboratory Diagnostics (Chinese Ministry of Education), College of Laboratory Medicine, Chongqing Medical University, Chongqing 400016, PR China; Key Laboratory of Clinical Laboratory Diagnostics (Chinese Ministry of Education), College of Laboratory Medicine, Chongqing Medical University, Chongqing 400016, PR China; The Center for Clinical Molecular Medical Detection, Biobank Center, The First Affiliated Hospital of Chongqing Medical University, Chongqing 400016, PR China; The Center for Clinical Molecular Medical Detection, Biobank Center, The First Affiliated Hospital of Chongqing Medical University, Chongqing 400016, PR China; State Key Laboratory of Oncogenes and Related Genes, School of Biomedical Engineering, Shanghai Jiao Tong University, Shanghai 200030, PR China; Key Laboratory of Clinical Laboratory Diagnostics (Chinese Ministry of Education), College of Laboratory Medicine, Chongqing Medical University, Chongqing 400016, PR China; Department of Neurosurgery, The First Affiliated Hospital of Chongqing Medical University, Chongqing 400016, PR China; Key Laboratory of Clinical Laboratory Diagnostics (Chinese Ministry of Education), College of Laboratory Medicine, Chongqing Medical University, Chongqing 400016, PR China; Key Laboratory of Clinical Laboratory Diagnostics (Chinese Ministry of Education), College of Laboratory Medicine, Chongqing Medical University, Chongqing 400016, PR China

## Abstract

Specific and sensitive DNA hybridization plays a key role in biotechnology, nanotechnology, and medical technology. However, traditional DNA hybridization-based strategies often require careful tuning of the binding affinity of the probe to attain a trade-off between specificity and sensitivity. Herein, we proposed energy barrier-gated dynamic selectivity to overcome this limitation. The mismatch closure-mediated strand displacement reaction (mcSDR) induces structural constraints through helper strand binding at mismatched sites, resulting in the displacement of the mismatch target requires overcoming an additional activation energy barrier, whereas the perfect match target proceeds via a normal pathway. The mcSDR has been thermodynamically and kinetically demonstrated to be able to balance specificity and sensitivity simultaneously. The energy barrier height can be programmably adjusted by design of helper strand and works in synergy with the toehold exchange strategy to achieve multi-parameter optimization. The superior properties of the mcSDR facilitated the identification of 12 mutation types exhibits excellent specificity in 28 clinically relevant single nucleotide variations. By combining polymerase chain reaction, mutations with an abundance of 0.1% were successfully detected in plasmid samples, and a triple mcSDR was successfully constructed. Clinical validation of 95 glioma and 93 colorectal cancer samples showed that IDH1 and KRAS mutations were 100% consistent with Sanger sequencing. The energy barrier-driven identification mechanism and operational simplicity of mcSDR make it promising for wide applications in biomedical research, molecular diagnosis, and precision medicine.

## Graphical Abstract

**Graphical Abstract:**
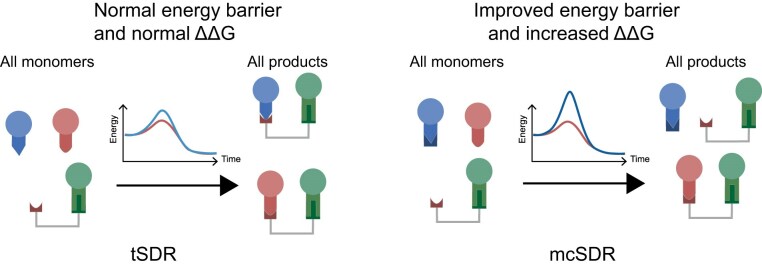

## Introduction

Key properties of nucleic acids make them useful in biology, biotechnology, and medical technology. Specific and sensitive DNA hybridization has long been the cornerstone of DNA-based tests, sensors, circuits, and materials [1–5]. Particularly in technology platforms that analyze minor alterations in nucleic acid, such as sequencing [6–8], allele-specific polymerase chain reaction (PCR) [9–11], molecular beacons [12–14], blocker displacement amplification [15, 16], probes with intricate structures [17, 18], and others [19–21]. There is an increased demand for enhanced sensitivity and specificity. In particular, single nucleotide variants (SNVs), which are of significant importance for the diagnosis, treatment, and prognosis of numerous cancers [22, 23], are present at a low frequency in the context of a large excess of wild-type DNA. Therefore, it is crucial to develop a method that can enhance specificity of nucleic acid hybridization to achieve higher-resolution identification.

An ideal system should comprise probe sets that exclusively react with the intended target (perfect match). However, there is a possibility of cross-hybridization occurring between probes and targets that are not perfectly paired [24]. Such imperfect probe-target binding can be kinetically trapped and thereby impeding the desired hybridization reaction. This is attributed to the thermodynamic gain of correctly paired bases, which can override the thermodynamic loss caused by mismatches [25], thereby posing challenges in distinguishing such mismatches. Improved methods have been developed to discriminate mismatches, mainly by increasing the yield of the intended hybridization (e.g. molecular beacons [12], altered probe lengths [26], or incorporation of artificial nucleobases [27, 28] etc.) or by implementing more stringent conditions to prevent unintended hybridization (e.g. adjusting temperatures [29], denaturing reagents [30, 31], or stoichiometry [32], etc.). In addition, using probes with Δ*G*_intended_ (Gibbs free energy of the intended target) close to zero, such as “strand-exchange probes” (TE probe) [25, 33], makes the yield of the system susceptible to minor standard free energy penalties arising from single-base mismatches (Supplementary Fig. S1). However, these methods inevitably cause Δ*G*_unintended_ evolving in the same direction as Δ*G*_intended_, and there is only a small change in ΔΔ*G* (the difference in the Gibbs free energy between the intended and unintended hybridization, ΔΔ*G* = Δ*G*_intended_ − Δ*G*_unintended_). In addition, improvements in sequence selectivity (specificity) and yield (sensitivity) have inherent limitations, with enhancement in one often leading to deterioration in the other. As a result, DNA hybridization is usually forced to find a compromise between acceptable specificity and sensitivity [34–36]. The direct increase of ΔΔ*G* in reaction is an effective approach [37, 38], but integrating kinetic regulation into a single-step hybridization event to simultaneously enhance sensitivity and specificity remains an ongoing challenge.

To address the aforementioned limitations, we have introduced a strategy to improve the specificity of strand displacement reaction (SDR) from a kinetic perspective. This strategy leverages complementary binding to close mismatched bases, thereby establishing a selective energy barrier. As a result, the SDR of the mismatched target is kinetically hindered by the transition state energy barrier, while the perfectly matched target (PM) proceeds along the conventional energy pathway. This approach not only enhances the discrimination between intended and unintended sequences, but also maintains high reaction yields, thus overcoming the inherent trade-offs in conventional approaches. The concept is illustrated through the application of theoretical modelling and experimental validation. Furthermore, the specificity of the reaction can be programmably adjusted by fine-tuning the design of helper strand and the intergrating with reverse toeholds. The reaction exhibits robustness towards variations in temperature, reaction buffer composition, and the presence of interfering strands. The mismatch closure-mediated strand displacement reaction (mcSDR) enabled the recognition of 12 types of mutations and the identification of 28 SNVs within mimic target. Subsequently, we integrated the methodology with PCR and extended its application to plasmid samples for the identification of SNVs in the KRAS, IDH1, and SARS-CoV-2 genes. The results demonstrated the successful achievement of detection down to an abundance as low as 0.1% and the successful construction of a triple-mcSDR. The practical utility was demonstrated by testing 95 glioma samples and 93 colorectal cancer samples. The successful implementation of this approach is anticipated to offer novel insights into highly specific and sensitive nucleic acid hybridization, particularly applicable to the analysis of SNV. The proposed strategy shows significant potential for application in the field of molecular diagnostics.

## Materials and methods

### Ethical statement

This study was approved by the Ethics Committee of the First Affiliated Hospital of Chongqing Medical University (no. 2023-406 and no. 2023-423). All research was performed under relevant guidelines and regulations.

### Materials and reagents

The DNA oligonucleotides were provided by Sangon Biotech Inc. (Shanghai, China). The 2 × SuperReal PreMix Color SYBR Green qPCR Mix (TG PCR Mix) was provided by TIANGEN (Beijing, China). The 2 × ChamQ Universal SYBR qPCR Master Mix (Vazyme PCR Mix) was provided by Vazyme (Nanjing, China). The 2 × Taq PCR Mix (Diomand PCR Mix) was provided by Diamond (Shanghai, China). The 2 × TaqMan Fast qPCR Master Mix (SG-TaqMan PCR Mix), SYBR Green, 8-strip real-time PCR tubes, and magnesium chloride hexahydrate (MgCl_2_·6H_2_O) were purchased from Sangon Biotech Inc. (Shanghai, China). N,N,N′,N′-tetramethylethylenediamine and 30% acrylamide/bis solution were provided by Sigma–Aldrich (St. Louis, USA). DNA loading buffer (6×) and Gel Red nucleic acid dye were ordered from TaKaRa Biotech (Dalian, China). The Lambda Exonuclease and Lambda Exonuclease Reaction Buffer (67 mM Glycine-KOH, 2.5 mM MgCl_2_, 50 μg/ml BSA, pH 9.4) were ordered from New England Biolabs (New England, USA). Nucleic acid extraction reagents were purchased from AmoyDx Biotech Inc. (Xiamen, China). All chemical reagents were of analytical grade, and RNase-free water was used throughout this study.

### Probe design

Most of the sequences were designed by NUPACK and Primer-BLAST, and some of them were subsequently modified by hand. Detailed sequences were shown in Supplementary Tables S1.

### Buffer conditions

All DNA oligonucleotides were redissolved in TE (pH 8), consisting of Tris–HCl (10 mM) and ethylenediaminetetraacetic acid (EDTA) (1 mM). The buffer used for all hybridization reactions was TE/Mg^2+^ buffer (pH 8), consisting of Tris–HCl (10 mM), EDTA (1 mM), and MgCl_2_ (12.5 mM), except for PCR amplification.

### Assembly procedure

All DNA complexes were assembled by mixing the corresponding single strands with equal molar concentrations in TE/ Mg^2+^ buffer (TEM). All samples were annealed in a PCR thermal cycler. The temperature was set at 95°C for 5 min initially, then decreased to 4°C by 0.1°C/s.

### Preparation of 12% polyacrylamide gel electrophoresis gel

Twenty-five milliliters of 12% polyacrylamide gels were prepared before. Five microliters of sample solution was mixed with 1 μl of 6× loading buffers and then loaded onto the 12% native polyacrylamide gel electrophoresis (PAGE) gels. Electrophoresis was performed in 1× TBE buffer (2 mM EDTA and 89 mM Tris-boric acid, pH 8.3) at a 100 V constant voltage for 60 min before staining with 4S GelRed. Finally, the gel was visualized under UV light by Bio-Rad ChemiDoc XRS imaging system (Bio-Rad Laboratories, American). The concentration of all nucleic acid strands was 250 nM, and the reaction was incubated at 37°C for 30 min before PAGE experiments.

### The mcSDR

Unless otherwise stated (Figs 2–4), the reaction volume was 20 μl, containing 250 nM target, 250 nM probe, and 250 nM helper at 37°C for 1 h.

### PCR protocol, enzyme digestion, and mcSDR

In a typical PCR protocol, 1 μl of DNA template, 10 μl of PCR mix, proper concentration (typically 250 nM) of forward and reverse primers, and 1 μl 20× SYBR Green of were mixed to a final volume of 20 μl. Thermal cycling started with a 3 min incubation step at 95°C for polymerase activation, followed by proper repeated cycles of 10 s at 95°C for DNA denaturing and 30 s at 60°C for annealing and extension. The product was further processed by λ exonuclease. As follows, proper concentration of enzyme (typically 1 μl) and 1 μl of enzyme buffer were added to 10 μl of PCR product, and the entire mixture was then gently mixed via pipetting. After incubation at 37°C for proper min (typically 30 min), the mixture was heated to 85°C for 10 min to deactivate the enzyme and then cooled back to room temperature. Finally, proper concentration of probe (typically 125 nM), proper concentration of Opener (typically 125 nM), proper concentration of Helper (typically 125 nM), and proper concentration of MgCl_2_ (typically 20 mM) were added to the digested product, which was supplemented to 20 μl with TEM for fluorescence monitoring.

The reactions (Figs 5 and 6) were performed at 37°C with a strand concentration of 125 nM unless otherwise stated. And they were all performed under optimized reaction conditions.

### Tissue DNA was extracted according to the manufacturer’s instructions

Pathological samples were obtained from the First Affiliated Hospital of Chongqing Medical University. After confirmation by HE staining, DNA extraction kit was used for extraction on a fully automatic tissue DNA extraction instrument. Finally, the total DNA concentration was determined by NanoDrop 1000 (Thermo Scientific) and stored at −80°C for future use. The A260/A280 value was between 1.8 and 2.0, indicating that the extracted DNA had high purity.

### Melt curve analysis

Melt curve analysis was performed using BioRad CFX96 Real Time System, C1000 Thermal Cycler (Hercules, CA, USA). SYBR dye needs to be added to the product to a concentration of 1× before melting curve analysis. A melt curve analysis was then performed with the reaction conditions of 65°C for 5 min followed by a continuous temperature ramp between 65°C and 95°C increasing at 0.5°C/s.

### Time-based fluorescence acquisition

Time-based fluorescence data were acquired using Rotor-Gene 6000 (Corbett Research, Mortlake, Australia). The fluorescent signals were monitored under the orange channel (585 nm/610 nm). For multiplex detection, the fluorescent signals were monitored under the yellow channel (530 nm/555 nm), orange channel (585 nm/610 nm), and red channel (625 nm/660 nm).

### Calculation of the discrimination factors

DF = (*F*_wt_− *F*_p_)/(*F*_mt_− *F*_p_), *F*_wt_ is the fluorescence signal generated by the perfectly matched target, *F*_mt_ is the fluorescence signal generated by the target containing the mutation site, and *F*_p_ is the fluorescence signal generated by the buffer only containing the probe.

### Fluorescence normalization

Typically, the original fluorescence intensities of the different states in the same set of probes are normalized via dividing the average of the highest value. Subsequently, the mean values of the normalized data under different states were calculated.

### Hybridization yield inference

The fluorescence values were converted to hybridization yield by the formula yield = (*F*_x_− *F*_b_)/(*F*_m_ − *F*_b_). where *F*_x_ is the observed fluorescence, *F*_b_ is the background fluorescence observed with the addition of buffer only, and *F*_m_ is the saturating fluorescence observed after adding a 40-fold excess of the correctly matched target.

### Kinetic fitting

The rate constant k is obtained by fitting the experimental data. Assuming displacement complexes quickly resolve into products or reactants, and therefore that all reactions can be fitted by instantaneous, second-order processes, we have


\begin{eqnarray*}
{\mathrm{A\;}} + {\mathrm{\;B}}\mathop \to \limits^{k1} {\mathrm{C\;}} + {\mathrm{\;D}},
\end{eqnarray*}

where A is the target complex, B is the probe, C is the product, and D is the output. The ordinary differential equation (ODE) equation of the reaction is


\begin{eqnarray*}
{\mathrm{d}}\left[ {\mathrm{A}} \right]/{\mathrm{dt}} = - {\mathrm{k}}1\left[ {\mathrm{A}} \right]\left[ {\mathrm{B}} \right],
\end{eqnarray*}

\begin{eqnarray*}
{\mathrm{d}}\left[ {\mathrm{B}} \right]/{\mathrm{dt}} = - {\mathrm{k}}1\left[ {\mathrm{A}} \right]\left[ {\mathrm{B}} \right],
\end{eqnarray*}

\begin{eqnarray*}
{\mathrm{d}}\left[ {\mathrm{C}} \right]/{\mathrm{dt}} = {\mathrm{k}}1\left[ {\mathrm{A}} \right]\left[ {\mathrm{B}} \right],
\end{eqnarray*}

\begin{eqnarray*}
{\mathrm{d}}\left[ {\mathrm{D}} \right]/{\mathrm{dt}} = {\mathrm{k}}1\left[ {\mathrm{A}} \right]\left[ {\mathrm{B}} \right],
\end{eqnarray*}

where k1 is the second-order rate constants of the SDR.

The time-dependent values of [D], the concentration of free output strand, can be inferred directly from the fluorimetry data: the intensity of reporter fluorescence is linearly dependent on [D] (as shown below). After converting fluorescence intensity to concentration via a linear relationship, we fitted the data obtained from the reporter system to estimate the rate constant (k) associated with the reaction contributing to the observed behavior. An objective function was defined to calculate the goodness of fit for a given set of rate constants. The function computes the difference between the normalized signal and the numerical solution obtained by solving the ODEs using the MATLAB function ode15s, from t_0_ to t_end_. An excessively restrictive additional penalty is imposed in the event that any of the parameters are negative. The objective function is minimized with MATLAB function *fminsearch*.

## Results and discussion

### Working principle of mcSDR

**Figure 1.**
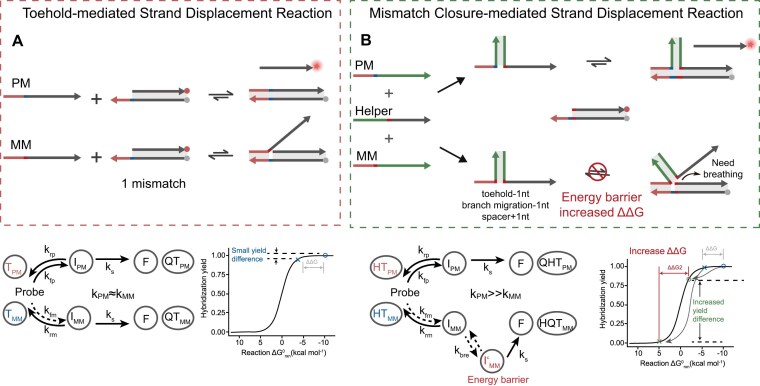
(**A**) Schematic diagram of a traditional strand displacement reaction (tSDR). (**B**) Schematic diagram of a mcSDR.

The SDRs have been widely used as a fundamental element in the field of DNA nanotechnology. As shown in Fig. 1A, the toehold-mediated strand displacement between a single-stranded input and a double-stranded substrate begins with a short complementary single-stranded domain on the substrate (referred to as toeholds) and proceeds by a process of branch migration similar to that of stochastic wandering [2, 39, 40]. The formation of additional base pairs enhances the thermodynamic favorability of the reaction. The kinetics of strand displacement is predominantly influenced by the length and sequence of the toehold domain when the toeholds are short. Depending on the binding strength (length and GC content) of the toehold, the rate constant can vary between 1 and 6 × 10^6^ M^-1^ s^-1^ [2, 41]. It has been observed that mutations occurring in or proximal to the toehold domain exert the most pronounced kinetic impact on strand displacement processes. However, when attempting to leverage these kinetic differences to discriminate between the intended target (referred to as PM) and the unintended target (referred to as MM), the achievable specificity remains constrained by the limited kinetic differences. In addition, the binding of either PM (circles) or MM (forks) to the probe is typically thermodynamically favorable and virtually indistinguishable at room temperature. Experimentally, we specifically designated the last base of the toehold as the mutation site and employed a traditional toehold-mediated strand displacement probe (TD probe) to distinguish PM and MM. The results showed that the discrimination factors (DFs) of PM and MM could decrease to 1 as the toehold length increases. Decreasing the toehold length leads to an increase in DF, but at the expense of yield. This situation holds regardless of whether C is mutated to A/T/G (Supplementary Fig. S2). The DF is significantly increased for single-base deletions, and this increase becomes more pronounced in the case of two-base deletions (Supplementary Figs S3 and S4). This is attributed to the fact that the deletion results in a ΔΔ*G* value that surpasses that of the mutation.

Inspired by this observation, we hypothesized that converting the mutation into a deletion-like one could potentially enhance the detection of SNVs. As shown in Fig. 1B, introducing a helper strand in the SDR closes the mutation site of the mismatched target (MM), consequently resulting in the shortening of both the toehold and branch migration domains by one nucleotide and the extension of the spacer region by one nucleotide. The intermediate with the three-way junction structure formed upon the toehold binding can only proceed after the initial base of the branch migration domain undergoes breathing. However, due to the low probability of base breathing, this would lead to an increase in the transition state energy barrier and a significant inhibition of the reaction rate. In contrast, the perfect match target completes the SDR along the normal reaction path. This design enables dual thermodynamic and kinetic selection: thermodynamically, the reaction exhibits a significant increase in ΔΔ*G*, while kinetically, the energy barrier–gated dynamic selectivity results in pronounced differences in reaction rates.

### Thermodynamics and kinetics of mcSDR

**Figure 2.**
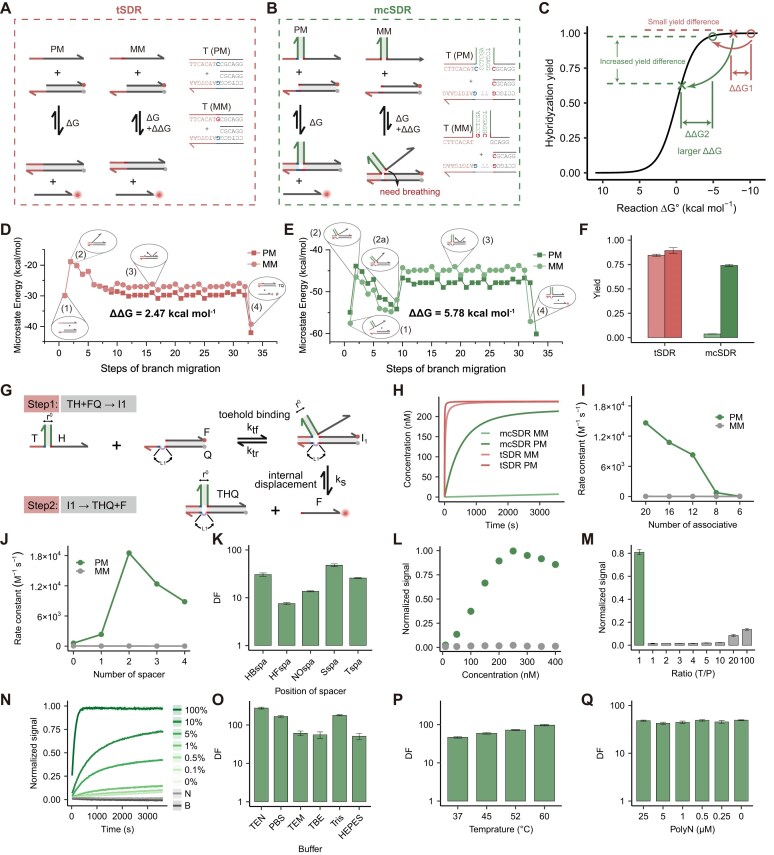
(**A**) Schematic illustration of the reaction between the target and the tTD probe. (**B**) Schematic illustration of the reaction between the target and the MC probe. (**C**) Graph of yield change with Δ*G*. Compared to the tSDR, the yield and ΔΔ*G* of mcSDR are observed to be greater. Perfect matches are indicated by circles, and mismatches are indicated by forks. (**D**) The energy landscape of tSDR. The tSDR process involves (i) the initial state of the reaction, (ii) toehold binding, (iii) branch migration, and (iv) incumbent separation. The mismatch between target and probe introduces an energetic penalty, which mainly increases the rate of back-off of the toehold binding step at the mismatch location. The presence of a mismatch leads to an overall elevated energy. (**E**) The energy landscape of mcSDR. The mcSDR process involves (i) the initial state of the reaction, (ii) toehold binding, (iia) base breathing, (iii) branch migration, and (iv) incumbent separation. The mismatch between the target and the probe reduces the number of toehold binding steps and branch migration steps by one step, respectively. (**F**) Yields of tSDR and mcSDR. (**G**) Reaction paths of PM targets. (**H**) Change in product concentration over time calculated using kinetic modeling. The dependence of the *k* value on (**I**) the length of the association domain and (**J**) the number of spacers. This was obtained by fitting the kinetics through the ordinary differential equation (ODE). (**K**) DF of spacer at different position. (**L**) mcSDR detection of different concentration ranges of targets. (**M**) Fluorescence signals generated by 250 nM PM and different concentrations of MM. T is the target concentration, P is the probe concentration, and the fixed probe concentration is 250 nM. (**N**) Fluorescence signals generated by different abundance targets. The probe worked robustly in (**O**) different temperatures, (**P**) different buffers, and (**Q**) different concentrations of interfering strands (PolyN) to distinguish mutations.

The SNV probes exemplify a challenge in balancing yield and selectivity, as the ΔΔG caused by single nucleotide mutation is minimal. In tSDR (Fig. 2A), the thermodynamic favorability of both the PM and MM renders them nearly indistinguishable (Fig. 2C). Mismatch closure-mediated strand displacement probes (MC probe) thermodynamically increase ΔΔ*G*. In addition, it kinetically raises the energy barrier for the MM target. Consequently, this enables the distinction between PM and MM (Fig. 2B and C).

The application of thermodynamic analyses facilitates the elucidation of the underlying mechanism of the reaction. First, we calculated the changes in free energy across various reaction states utilizing Multistrand based on the model proposed by Winfree’s group [33, 42]. And the energy landscape results are depicted in Fig. 2D and E. The process and sequence of the tSDR are shown in Supplementary Figs S5 and S6. The results showed an increase in the free energy of the single base mismatch sequence (MM, light line) compared to the perfect match sequence (PM, dark line) (Fig. 2D). Nevertheless, the increased energy penalty can be readily overcome by the correctly paired bases. The process and sequence of the mcSDR are illustrated in Supplementary Figs S7 and S8. In comparison to tSDR, the MM target lacks a free base that binds to the final base of the toehold in mcSDR. Furthermore, only when the first pair of bases in the branch migration domain undergoes breathing. Thus, this leads to a higher activation energy barrier for MM (Fig. 2E). The capacity of the two methods to distinguish between single base mismatches was assessed based on the difference in free energy change (ΔΔ*G*) [ΔΔ*G* = (ΔG__Initial_MM_ − ΔG__Final_MM_) − (ΔG__Initial_PM_ − ΔG__Final_PM_)]. In this design, the ΔΔ *G* was calculated to be 2.47 kcal/mol for the tSDR, whereas the mcSDR exhibited a higher ΔΔ*G* of 5.78 kcal/mol (Supplementary Fig. S9). Experimentally, the fluorescent group and quenching group are respectively modified at the ends of the F strand and Q strand. When the target complex and the probe for reporting reacted, the F strand was displaced and fluorescence was restored. As shown in Fig. 2F, the yield of PM in MC probe (dark red) was much higher than that of MM (light red). In contrast, the yield of PM in TD probe (dark green) was almost indistinguishable from that of MM (light green). While TE probe enhanced selectivity, this improvement was accompanied by a reduction in yield (Supplementary Figs S10 and S11). Overall, the results demonstrate that mcSDR can effectively enhance the selectivity of nucleic acid hybridization, thereby facilitating the detection of SNVs.

Subsequently, we constructed a mathematical model to characterize the kinetics of the reaction. The reaction mechanism of the mcSDR is similar to the model proposed by Andrew J. Turberfield for the remote toehold [43]. The main reaction pathway is as follows (Fig. 2G and Supplementary Fig. S12): Initially, the target (T) engages with the substrate (FQ) through the toehold domain; the branch migration domain then proceeds to explore the surrounding volume at the docking site, which represents an internal diffusion step (Supplementary Fig. S13). This step is crucial for aligning the displacement domains and initiating the branch migration reaction, as it leads to displacement of the F strand from the substrate. When the target is MM, base breath after toehold binding is necessary for subsequent reactions. This step is the primary rate-limiting step of the reaction, resulting in an increase in the transition state energy barrier. The results of the simulation demonstrated a rate of 1.2 × 10^4^ M^−1^ s^−1^ for PM and 37 M^−1^ s^−1^ for MM (Fig. 2H). Although the overall rate is slightly lower in comparison to tSDR, the difference in rate between PM and MM can vary by three orders of magnitude. The above results demonstrate that the specificity of the SDR can be significantly enhanced through the energy barrier-gated dynamic selectivity.

### Characterization and performance of mcSDR

Firstly, PAGE was employed to analyze mcSDR (Supplementary Fig. S14). PAGE analysis revealed that only PM yielded discernible product bands, whereas MM did not, thereby confirming the assertion that mcSDR is markedly susceptible to mismatches. Next, we systematically investigated two pivotal parameters affecting the reaction: the length of the association domain (depicted in green) and the number of spacers (depicted in purple) (Fig. 2G). To assess the binding affinity of association domains, we designed domains with varying lengths. The free energy values obtained from NUPACK predictions ranged from −9.64 to −31.85 kcal/mol (Supplementary Fig. S15). The rate constant of the reaction was calculated by real-time monitoring the fluorescence (Fig. 2I). The results demonstrate a positive correlation between the length of the associated domain and the stability of the combination, leading to an accelerated reaction rate. However, it should be noted that there is also a slight decrease in DF (Supplementary Fig. S15). To ensure both reaction speed and specificity, it is recommended that the length of the association domain should preferably be greater than 12 nt.

Subsequently, we investigated the effect of the number of spacers on the kinetic performance of the reaction. A series of bottom strands (Q strands) was designed with spacer lengths ranging from 0 to 4 nt, and the reaction was monitored by measuring fluorescence intensity (Supplementary Fig. S16). As shown in Fig. 2J, the presence of spacer domains accelerates the reaction considerably. In the bottom strand (Q strand) with a spacer domain length of 2 nt, the maximum reaction rate was observed, and the rate decreased with the elongation or shortening of the spacer domain. In contrast, the maximum DF was achieved in the bottom strand (Q strand) of 4 nt (Supplementary Fig. S16). This is because an increase in spacer length results in a reduction in the probability of alignment within the internal diffusion of the reaction, particularly for MM targets. This ultimately leads to an elevated DF value. We then explored the effect of different spacer positions on the reaction performance. The reaction was monitored by real-time fluorescence curves, and the DF was subsequently calculated (Supplementary Figs S17–S19). The results presented in Fig. 2K demonstrate that, for a given toehold length, the spacer on the bottom strand yielded the highest DF. It is plausible that the spacer on the helper strand or target strand exerts an influence on base closure. Furthermore, the signal and DF can be enhanced when the concentration of the probe approaches closer to the target concentration. (Supplementary Fig. S20).

We aimed to investigate the potential integration of our method with existing approaches in order to enhance the accuracy of mutation identification. By integrating the toehold exchange strategy in mcSDR, real-time fluorescence curves were recorded to reflect the reaction progress and the DF was calculated (Supplementary Fig. S21–S23). The results indicated that a gradual elongation in the length of the reverse toehold under various forward toehold conditions can enhance the DF. However, as with conventional toehold exchange reactions, the longer reverse toehold results in a gradual decline in yield. The above results suggest that the mcSDR strategy can be combined with the toehold exchange strategy that regulates the reaction Δ*G*, thereby further fine-tuning the specificity of the reaction.

To further quantify the performance of mcSDR over a wide range of concentrations, fluorescence signals generated by targets from 10 nM to 400 nM were collected. The presence of PM resulted in a significant increase in fluorescence, and the generated normalized signal showed a positive correlation with target levels within a certain range (10–250 nM) (Supplementary Fig. S24). In contrast, MM hardly generates any fluorescence signal, demonstrating the high specificity of the reaction. It is noteworthy that the high specificity was maintained even at high concentrations (Fig. 2L). This prompted us to investigate the performance of mcSDR at higher stoichiometric ratios of targets and probes (T/P) ratios. Supplementary Fig. S25 illustrated the fluorescence response of the probe to high concentrations of PM and MM. mcSDR showed significantly reduced yields even when confronted with a 100-fold excess of MM targets in comparison to the probe (Fig. 2M). When T/P is within 10, the MM yield approaches zero, while DF is between 35 and 56. (Supplementary Fig. S25). Subsequently, the specified mixtures of PM and MM (total concentration of 250 nM) were employed to assess the detection limit of the strategy. Even if the abundance of PM is only 0.1%, statistically differences in fluorescence signals can still be observed compared to 0% PM (Fig. 2N and Supplementary Fig. S26).

The capacity to directly detect nucleic acids under diverse conditions with exceptional performance significantly streamlines the process of molecular diagnostics. This enables the direct analysis of biological samples or amplification products without the need for separate purification and/or buffer change procedures [44]. The robustness of the mcSDR was further validated. Experimentally, the mcSDR exhibited excellent discriminatory ability across a diverse array of biological buffers (1 × TEN, 1 × PBS, 1 × TEM, 1 × TBE, 1 × Tris, and 1 × HEPES) (Supplementary Fig. S27) and at various temperatures (37–60°C) (Supplementary Figs S28–S31) (Fig. 2O–P). As illustrated in Fig. 2Q and Supplementary Fig. S32, a mixture of 50-nt sequences at concentrations up to 25 μM exhibited robust discrimination. Next, we examined how quickly our probes could distinguish between PM and MM. To this end, we calculated the DF over time curve (Supplementary Fig. S33). The results show that the DF of mcSDR can reach around 60 within 1000 s, and remains a relatively stable level as time progresses. In contrast, PM and MM almost rapidly and nearly completely bind to the probe in the tSDR. This limited differentiation in the initial phase of the reaction (before 500s) increases the probability of a false-positive result.

### Increase the number of closures

**Figure 3.**
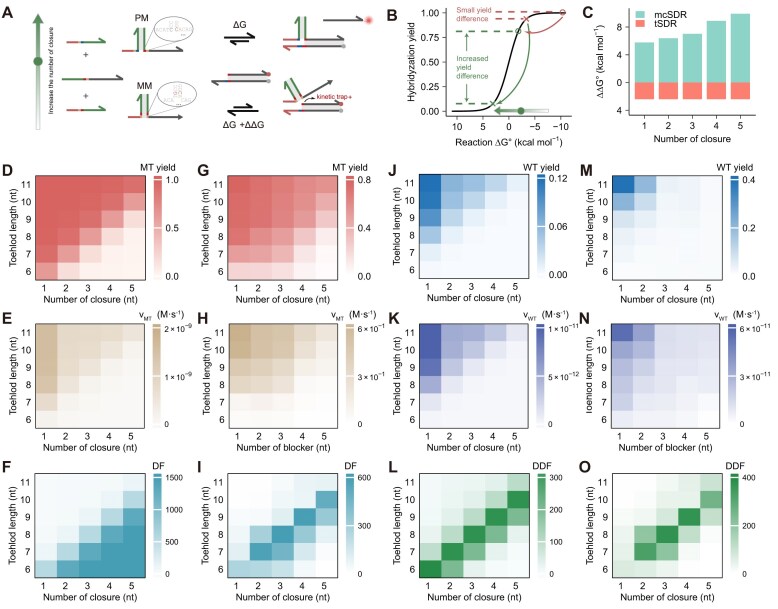
(**A**) Schematic diagram of mcSDR with increasing number of closures. (**B**) Graph of yield change with Δ*G*. (**C**) Comparison of ΔΔ*G* with tSDR for mcSDR with varying number of closures. Simulation of the yields of (**D**) PM target and (**J**) MM target with different toehold lengths under different number of closures. Simulate the apparent rates of (**E**) PM target and (**K**) MM target with different toehold lengths under different number of closures. Simulation of (**F**) DF and (**L**) DDF (Yield _(PM)_^2^/Yield_(MM)_) with different toehold lengths under different number of closures. Experimental validation of the yields of (**G**) PM target and (**M**) MM target at different number of closures with different toehold lengths. Experimental validation of the apparent rates of (**H**) PM target and (**N**) MM target at different number of closures with different toehold length. Experimental validation of (**I**) DF and (**O**) DDF at different number of closures with different toehold length.

Subsequently, we conducted a more detailed investigation into the impact of increasing the number of closures on the reaction, with a focus on elucidating the underlying mechanisms and identifying potential optimization strategies (Fig. 3A). When the target is PM, the mismatch between the helper strand and the target leads to an unstable closed domain, resulting in rapid completion of the SDR. Whereas when the target is MM, stable binding between the helper strand and target results in a reduction of available toehold length. The thermodynamic increase of the paired base with a short toehold may not cover the energy penalty caused by the mismatched base, thus causing the free energy of the reaction to move to the positive region (Fig. 3B). Thermodynamically, we compared the ΔΔ*G* of tSDR with that of mcSDR with 1–5 enclosed bases. Figure 3C shown a positive correlation between the number of closures and an increase in ΔΔ*G*. The DF versus Δ*G* plots of tSDR and mcSDR with different number of closures were calculated (Supplementary Fig. S34). The results indicated that an increase in the number of closures is associated with a corresponding rise in the DF value. Kinetically, a three-step model was devised for mcSDRs with multiple closures (Supplementary Fig. S35), in contrast to the aforementioned two-step model established for αmcSDRs. The first step is toehold binding (light red domains), followed by branch migration on the toehold domains (yellow domains), and finally an internal diffusion step.

Compared to PM, the reaction of MM with a probe necessitates additional breathing steps. By modeling the kinetics, we calculated the MT yield (corresponding to PM), WT yield (corresponding to MM), MT rate, WT rate, DF, and DDF for varying number of closures and different toehold lengths (Fig. 3D, J, E, and K). DF is used to reflect the specificity of the assay. Although an increased number of closures and a shorter toehold length would result in a greater DF but could compromise sensitivity in practical detection. Thus, the parameter DDF is defined to comprehensively capture the sensitivity and specificity. The simulations revealed a significant difference of approximately three orders of magnitude in the reaction rates between PM and MM as the toehold length was increased from 6 to 11 and the number of closures was increased from 1 to 5. The DF value can be up to 1588 (Fig. 3F). The DDF results show that different number of closures possess the respective optimal length of toehold (Fig. 3L).

Experimentally, we conducted a series of fluorescence kinetic experiments on targets with varying toehold lengths at different numbers of closures (Supplementary Figs S36 and S37). Similarly, the MT yield (corresponding to PM), WT yield (corresponding to MM), MT rate, WT rate, DF, and DDF of the reaction were calculated (Fig. 3G, M, H, and N). The experimental results demonstrate a consistent trend with the simulation results, thereby substantiating the successful construction of the kinetic model. The DF values yield disparate results when in cases of excessive closures or insufficient toehold length (Fig. 3I). This discrepancy arises from the challenge of detecting lower yields accurately within the experimental assay, thus leading to a low calculated DF. Figure 3O shows that DDF effective depicts a compromise between sensitivity and specificity.

### Detection of different mutations

**Figure 4.**
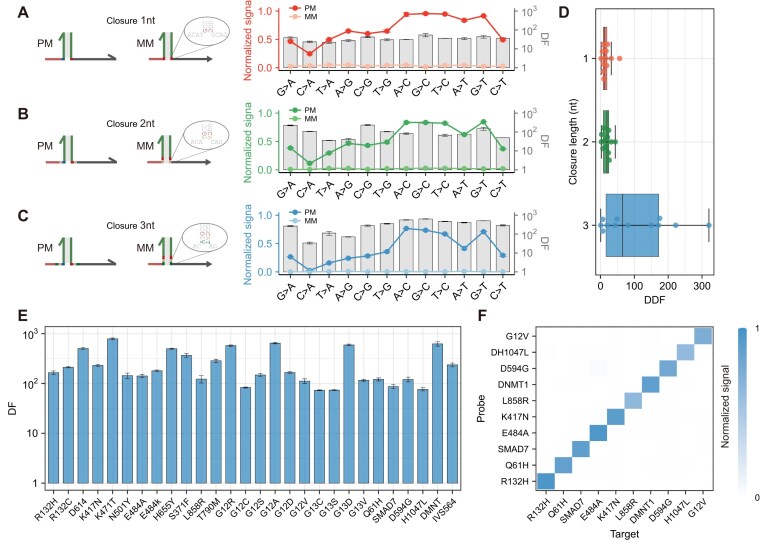
Normalized signal values and DFs of intended targets and SNVs observed for different mutation types when mcSDR was closed: (**A**) 1 nt, (**B**) 2 nt, (**C**) 3 nt. (**D**) Comparison of DDF produced by targets with different mutation types at different closure numbers. (**E**) Detection of 28 clinically or biologically important SNV mutant sequences and their corresponding WTs by experimentally calculated DF. (**F**) Validation of orthogonality analysis of 10 randomly selected targets by mcSDR.

Given the extensive diversity of SNVs encountered in the clinical settings, we proceeded to investigate the potential of mcSDR for effective discrimination across a range of other mutation types. We have designed all types of mutations and detect both 100% variant allelic frequency (VAF) and 0% VAF. The results demonstrated that each type of mutation could be effectively identified (Fig. 4A and Supplementary Fig. S38) and that the median DF value was 35.7 (Supplementary Fig. S41). Following an increase in the number of closures, we further explored the ability of this strategy to recognize different types of mutations. As illustrated in Fig 4B and C, the fluorescence intensities generated by both PM and MM exhibited a slight decline with the increase in the number of closures (Supplementary Figs S39–S41). However, the median DFs were elevated to 93.6 (γmcSDR) and 323 (ϵmcSDR), respectively, with the highest DF reaching up to 633 (Supplementary Fig. S41). By calculating DDF, we found that ϵmcSDR showed the best performance (Fig. 4D), so it was used in subsequent experiments. Among the observed outcomes, the conversion of C to A mutation exhibits a relatively low yield, which may be attributed to sequence variability or secondary structure. In general, mcSDR is highly selective for the majority of mutation types, which is seldom achieved by existing methods.

To further validate their specificity in complex sequence environments, we also designed helper strands and reporter probes for 28 biologically important SNVs in 9 genes, including a variety of clinically relevant tumor-associated SNVs and SARS-CoV-2 subtype classification genes, among others. Many conventional methods are hard to distinguish certain challenging mutations at single-base resolution, such as EGFR_L858R, which possesses a complex GC content [19]. The SNV and WT fluorescence values of 28 targets were detected by ϵmcSDR (Supplementary Figs S42 and S43), and the calculated DFs ranged from 73 to 790 (mean = 268, median = 166) (Fig. 4E). In the majority of cases, the alteration of the sequence at the 5′ end of the helper strand is sufficient for target detection. In a few cases, optimal discrimination can be achieved by fine-tuning the number of branch migration domains or toeholds of the probe (Supplementary Fig. S44). The preincubation of targets and helper strands was observed to accelerate the reaction rate of specific targets with strong secondary structure (Supplementary Fig. S45). Furthermore, the low cross-reactivity of mcSDR was demonstrated by randomly selecting 10 targets out of the 28 mentioned above (Fig. 4F and Supplementary Fig. S46). The potential for conducting multiple tests on mcSDR was also explored. The results presented in Supplementary Figs S47–S50 demonstrate that mcSDR effectively differentiated all SNV combinations in triple testing. Overall, these results demonstrate that mcSDR exhibits excellent selectivity for multiple types of SNVs.

### Detection of SNV in plasmid samples in combination with PCR

**Figure 5.**
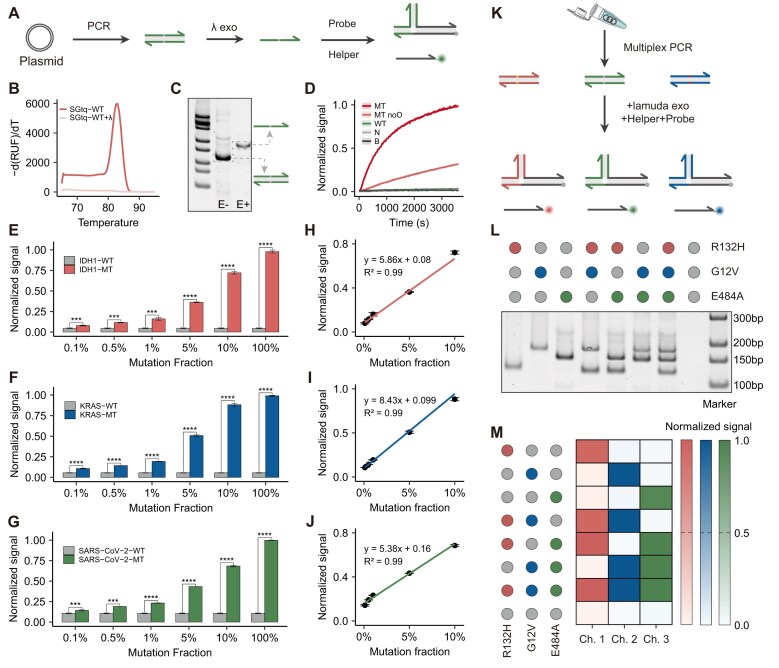
(**A**) Workflow for analyzing SNV in plasmid samples using PCR and MC probes. (**B**) Comparison of Tm before and after treatment with λ exonuclease. (**C**) PAGE analysis of PCR products before and after λ exonuclease treatment. (**D**) Fluorescence curve of mcSDR to analyze PCR products of SNV targets. Evaluating the sensitivity of mcSDR for analyzing (**E**) IDH1, (**F**) KRAS, and (**G**) SARS-CoV-2 in plasmid samples with varying mutation abundance from 0.1% to 100%. Unpaired two-tailed *t*-tests were used to evaluate statistical differences between samples. ***P < .01, ****P < .0001. Normalized signals of (**H**) IDH1, (**I**) KRAS, and (**J**) SARS-CoV-2 were linearly related to mutation abundance from 0.1% to 10%. (**K**) Multiplex mcSDR assay. The schematic of the workflow of 3-plex mcSDR for simultaneous analysis of IDH1, KRAS, and SARS-CoV-2 mutations in plasmid samples. (**L**) PAGE analysis of the products of 3-plex PCR. (**M**) Assessment of plasmid sample combinations and their compatibility with MC probe mixtures used for the analysis IDH1, KRAS, and SARS-CoV-2. Technical replicates were performed for each sample (*n* = 3).

The robustness of mcSDR to solution and temperature is expected to be ideally adapted to a variety of amplification methods for enhancing sensitivity. Accordingly, we aimed to investigate the effective coupling of mcSDR with amplification techniques. The PCR was employed in this study due to its exceptional sensitivity and excellent reproducibility. A typical workflow for analyzing SNVs is illustrated in Fig. 5A. This process involves the amplification of the target by PCR, followed by digestion with λ exonuclease and direct monitoring of the fluorescence signal using probes. As a proof of concept, three distinct systems were designed for IDH1 R132H, KRAS G12V, and SARS-CoV-2 E484A.

It was demonstrated by real-time fluorescence curves that phosphate-modified primer-mediated PCR could effectively detect targets at 10 fM in different PCR mix (Supplementary Fig. S51). The efficacy of λ exonuclease (λ-exo) acquisition in generating single-stranded products was evaluated through melting curves and PAGE experiments. The melting curve analysis demonstrated that the PCR product exhibited a distinctive melting peak, with a Tm value of 83°C. After λ-exo digestion, the melting peak decreased sharply (Fig. 5B and Supplementary Fig. S52). The PAGE results demonstrated that following digestion by λ-exo, the original product bands were no longer visible while new bands emerged (Fig. 5C). These results proved that λ-exo successfully digested the 5′ phosphorylated one of the duplex. We further performed real-time fluorescence monitoring of the reactions. As illustrated in Fig. 5D and Supplementary Fig. S53, the increased fluorescence of the WT target was barely visible, while the fluorescence of the MT target increased significantly, despite its slower kinetics. It was hypothesized that this slow kinetics may be due to the fact that long single-stranded DNA (ssDNA) is prone to forming complex secondary structures (Supplementary Fig. S54). The introduction of an opener was implemented to mitigate the impact of ssDNA secondary structure on the reaction. The results demonstrated that the incorporation of the opener strand led to a notable enhancement in fluorescence intensity and an accelerated rate (Fig. 5D). PAGE experiments were also employed to verify the successful completion of the reaction (Supplementary Fig. S55). The similar performance was reached in the KRAS and SARS-CoV-2 assays (Supplementary Figs S56–S65).

In practice, mutant DNA is usually present in populations containing a substantial quantity of wild-type DNA. It was therefore essential to assess the efficacy of the probes in the context of wild-type interference. To achieve optimal performance, the Mg concentration, the enzyme dosage, the enzyme digestion time, and the probe concentration were optimized (see Supplementary Figs S66–S68). Under the optimized conditions, we used the indicated mixtures to evaluate the detection limits of the strategy. As shown in Fig. 5E–G, the fluorescence intensity was gradually enhanced with increasing abundance of MT targets. mcSDR demonstrated high selectivity for single-base mutations across all three targets. Even when only 0.1% of the intended target was present in the initial template mixture, statistically significant differences in fluorescence signals were still observed. The proposed method demonstrated satisfactory detection limits in comparison to existing methods (Supplementary Table S2). Moreover, a linear correlation was identified between the normalized fluorescence and the mutation fraction within the range of 0.1%–10% (Fig. 3H–J and Supplementary Figs S69 and S71). Thus, we demonstrate that mcSDR can be integrated with PCR amplification and easily adapted to a diverse array of mutant targets through simple sequence design.

We then aimed to ascertain whether mcSDR could simultaneously detect a multitude of genetic variants while maintaining high selectivity. The multiplexed detection of nucleic acids allows for inferring more comprehensive genomic information and improving genotyping throughput [45]. As a proof of concept, the three mcSDR panels described above were designed and hybridized for the simultaneous detection of IDH1 R132H, KRAS G12V, and SARS-CoV-2 E484A (Fig. 5K). Each reporter probe was labeled with a distinct fluorophore, and no overlapping spectra were observed between fluorophores (Supplementary Fig. S72). The PAGE results showed that the three SNVs could be efficiently amplified simultaneously in different groups (Fig. 5L). Despite the incorporating of six helper strands and three reporter probes, mcSDR achieved effective discrimination of all SNVs in different combinations of the three targets (Fig. 5M and Supplementary Figs S73–S75). Consequently, the mcSDR remained highly selective in multiple assays.

### Testing of clinical samples

After demonstrating the ultra-high selectivity of mcSDR for analyzing plasmid samples, mcSDR was next used to challenge the detection of cancer tissue samples in a real clinical setting. Two clinically significant SNVs, IDH1 R132H, and KRAS G12V, were selected as targets for further investigation. The R132H mutation is the most prevalent glioma-associated isocitrate dehydrogenase (IDH) mutation and is included in the National Comprehensive Cancer Network (NCCN) guidelines [46, 47]. Patients with glioma exhibit a more favorable prognosis following an IDH1 mutation. The G12V on codon 12 of KRAS is considered one of the most significant mutations associated with CRC and is regarded as a pivotal target for tumor therapy [48, 49].

**Figure 6.**
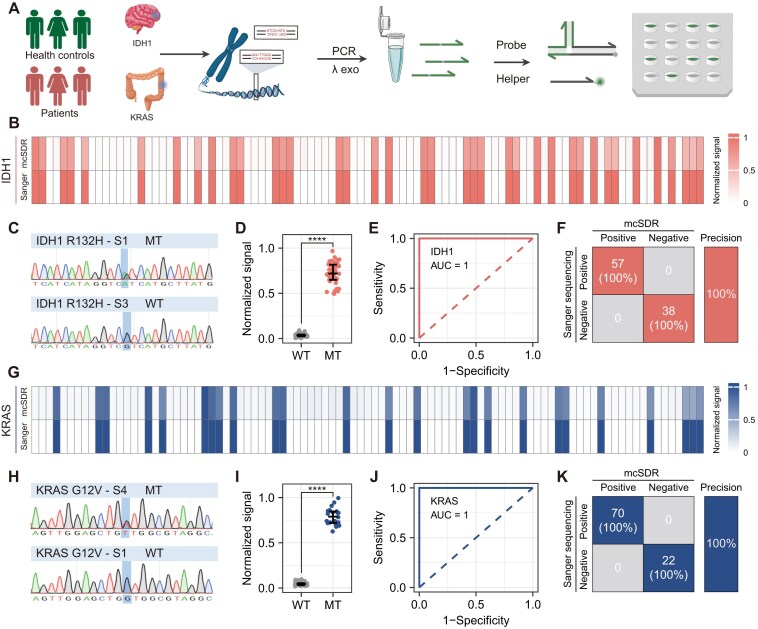
(**A**) Schematic of the workflow for analyzing clinical glioma and colorectal cancer tissues with MC probes after PCR enrichment. (**B**) Heatmap of results on 95 glioma tissue samples using MC probes and Sanger sequencing. (**C**) Partial Sanger sequencing results of IDH1 mutation sites. (**D**) Normalized signals in glioma samples from two clinical cohorts, including 38 positive and 57 negative samples. The normalized signals in the positive cohort were significantly higher than those in the negative cohort (*P* < .0001). (**E**) ROC curve used to determine the diagnostic accuracy and threshold of the proposed strategy in glioma samples. (**F**) Sensitivity and specificity of MC probes compared to Sanger sequencing in glioma samples was assessed using a confusion matrix (*n* = 95). (**G**) Heatmap of the results of 95 colorectal cancer tissue samples using MC probes and Sanger sequencing. (**H**) Partial sanger sequencing results of KRAS mutation sites. (**I**) Normalized signals in colorectal cancer samples from two clinical cohorts, including 22 positive and 71 negative samples. The normalized signals in the positive cohort were significantly higher than those in the negative cohort (*P* < .0001). (**J**) ROC curve used to determine the diagnostic accuracy and threshold of the proposed strategy in colorectal cancer samples. (**K**) Sensitivity and specificity of MC probes compared to Sanger sequencing in colorectal cancer samples was assessed using a confusion matrix (*n* = 93). Statistical differences between cohorts were assessed using unpaired two-tailed *t*-tests. All experiments were repeated three times.

Tissue samples were collected from patients who underwent surgery for brain glioma (*n* = 95) and colorectal cancer (*n* = 93) at the First Affiliated Hospital of Chongqing Medical University (Supplementary Tables S3 and S4). Genomic DNA (gDNA) is extracted from clinical tissue samples using a commercial gDNA extraction kit. Considering the potential problems of DNA degradation, storage conditions, and time, we initially quantified the extracted DNA concentration by NanoDrop. The measured gDNA concentration was approximately 100–400 ng/μl, which was suitable for subsequent PCR. Following the PCR enrichment process, the product was cleaved by λ exonuclease to produce ssDNA. This was followed by the addition of the helper strand, opener, and probe to analyze the reaction product (Fig. 6A). By monitoring real-time fluorescence curves of clinical samples, the mcSDR method identified 38 positive and 57 negative samples in glioma samples (Fig. 6B and Supplementary Figs S76–S79) and 22 positive and 71 negative samples in colorectal cancer samples (Fig. 6G and Supplementary Figs S80–S83). All samples were subjected to simultaneous validation through standard Sanger sequencing (Fig. 6C and H, and Supplementary Figs S84–S91). The normalized fluorescence values of the mutant targets were significantly higher than those of the wild-type targets (Fig. 6D and I). The area under the curve of the receiver operating characteristic (ROC) was 1 (Fig. 6E and J). The sensitivity and specificity were 100% and 100%, respectively, compared with Sanger sequencing (Fig. 6F and K). The above results emphasize the potential applicability and feasibility of our method in practical clinical scenarios.

## Conclusion

The capacity to identify SNVs is crucial for the diagnosis, treatment, and prognosis of diseases and cancers. This is because the majority of genetic predispositions originate from single-point mutations in narrow regions within a few genes. In conventional hybridization reactions, sensitivity and specificity are negatively correlated, implying that high specificity is often accompanied by a low yield. Compared with methods designed only from a thermodynamic [25, 37] or kinetic perspective [50, 51], combining kinetic and thermodynamic design is a more useful strategy to improve detection performance, such as sink probes [20, 35, 52], etc. In contrast to the sink strategy, which splits the kinetic pathways of the correct and mutant targets into two parts, we developed a promising technology for the effective detection of SNVs by introducing energy barrier-gated dynamic selectivity. The mcSDR strategy was demonstrated to achieve decoupling of sensitivity and specificity through simulations and experiments. This is because mcSDR is capable of utilizing base pair breathing to restrict individual hybridization processes within spatially separated regions. By controlling the length of the association domain, the number and position of spacers, and the number of closures, the specificity of the reaction can be more precisely tuned. Furthermore, mcSDR can be combined with methods typically used to increase binding specificity, such as toehold exchange strategies. The mcSDR displayed strong robustness in response to alterations in temperature, buffer conditions, and in the presence of high levels of non-homologous DNA interference. Our strategy showed exceptional reliability in detecting diverse mutation types with a median DF = 323 (ϵmcSDR). The identification of 28 different SNVs substantiates the versatility of mcSDR. The high specificity of mcSDR stems from a combination of two key factors:

- Increased ΔΔG: The MM targets in TD probe remain thermodynamically favorable, which are limited to distinguishing nucleic acids at the single base level based on initial kinetics rather than thermodynamic equilibrium. With our rationally designed probe, the toehold domain is reduced by 1 nt and the branching migration domain is also reduced by 1 nt in the SDR when the target was MM. In contrast, the PM target is unaffected, thereby causing a significant difference of the free energy change between PM and MM.
- Energy barrier-gated dynamic selectivity: Given that the bases used for branch migration in MM targets are initially enclosed, it is imperative that breathing of the first base of the branch migration domain occur prior to the subsequent initiation of branch migration. The probability of spontaneous base-pair breathing is relatively low in comparison to base-pair substitutions, which can result in the establishment of persistent kinetic limitations of MM targets. tSDRs can only rely on initial kinetics to distinguish nucleic acids at the single-base level during the initial minutes. However, mcSDR is able to identify the mutant targets within the initial 1000s of minutes after the start of the reaction, with the fluorescent signal retaining its discriminatory ability in subsequent assays.

Combining amplification reactions can be effective in increasing the sensitivity of the reaction. To this end, we employed PCR to pre-amplify plasmid samples and converting them to long ssDNA with a toehold through an enzymatic processing step. Three probes were designed for the detection of glioma-associated IDH1, colorectal cancer-associated KRAS, and SARS-CoV-2-associated E484A. The results reveal that mcSDR is an effective tool for distinguishing single base changes and can detect mutations down to 0.1% abundance. Furthermore, mcSDR allows for the simultaneous detection of multiple mutations in multiple genes, where three different fluorophore-labeled probe sets simultaneously function in the same detection system. In terms of clinical applications, mcSDR has been employed for the analysis of brain glioma and colorectal cancer tissue samples. Our probes demonstrate excellent performance, making them a powerful tool for developing reliable SNV identification.

In summary, the main advantages of mcSDR over tSDRs are as follows: (i) It is capable of achieving high single-base resolution at specific sites of the target, thus enabling the detection of low-abundance samples. (ii) The robustness of mcSDR permits its operation under a range of reaction conditions with minimal need for complex adjustment of experimental conditions. (iii) mcSDR achieves consistent specificity and sensitivity with thermodynamic and kinetic advantages, thereby reducing the necessity for experimental optimization and, consequently, simplifying probe design and application costs. (iv) The mcSDR is compatible with any upstream amplification technology, such as PCR and recombinase polymerase amplification (RPA) [53]. Moreover, the highly programmable nature of the SDR allows for the expectation that the mcSDR may be integrated with multiple downstream DNA amplifiers, including but not limited to catalytic hairpin assembly [54] and hybridization chain reaction [55, 56]. Thus, mcSDR is expected to be deployable as a POC test for analyzing cancer-associated SNVs or drug-resistant mutations in infectious diseases under resource-limited conditions. The applications of mcSDR are not only in biotechnological approaches that rely on highly specific nucleic acid hybridization, but are also expected to find further applications in areas such as DNA circuits, *in vivo* imaging, and drug delivery. Consequently, mcSDR is a promising approach to further advance biomedical sensing and diagnostics.

## Supplementary Material

gkaf660_Supplemental_File

## Data Availability

All data supporting the findings of this study are available within the article and its supplementary information or will be made available from the authors upon request.
