# Supplementary material for: Design of mismatch closure for enhanced specificity in DNA strand displacement reactions
Source: Nucleic Acids Res. 2025 Jul 12;53(13):gkaf660. doi: 10.1093/nar/gkaf660 (PMC12255293; doi:10.1093/nar/gkaf660)
Supplement: gkaf660_Supplemental_File [file gkaf660_supplemental_file.pdf]

Supporting Information

# Design of Mismatch Closure for Enhanced Specificity in DNA Strand Displacement Reactions

Hongyan Yu[1,#], Xiaole Han[1,#], Li Zhang [1,#],Na Yin[1,#], Li Wang[2], Ke Lv[4],Yongchang Wu[1], Dan Bai[1], Weitao Wang[†], Ying Huang[2], Xingping Hu[2], Zhi Weng[3], Chenlu Zhang[1], Gang Yang[*,4], Tingmei Chen[*,1],and Guoming Xie[*,1]

[1]Key Laboratory of Clinical Laboratory Diagnostics (Chinese Ministry of Education), College of Laboratory Medicine, Chongqing Medical University, Chongqing, 400016, PR China.

[2]The Center for Clinical Molecular Medical Detection, Biobank Center, The First Affiliated Hospital of Chongqing Medical University, Chongqing, 400016, PR China.

[3]State Key Laboratory of Oncogenes and Related Genes, School of Biomedical Engineering, Shanghai Jiao Tong University, Shanghai, 200030, PR China

[4]Department of Neurosurgery, The First Affiliated Hospital of Chongqing Medical University, Chongqing 400016, PR China

[*] To whom correspondence should be addressed. Tel: +86 23 68485240; Fax: +86 23 68485239; Email: guomingxie@cqmu.edu.cn

Correspondence may also be addressed to Gang Yang. Email: gangyang@hospital.cqmu.edu.cn and Tingmei Chen Email: tingmeichen@cqmu.edu.cn

[#]The authors wish it to be known that, in their opinion, the first and second authors should be regarded as Joint First Authors.

## S1 Worm-like chain model of the internal displacement rate

In the following section, we applied the wormlike chain model to explain the effect of spacer length on displacement speed. (1,2)

We first develop a probability distribution for the end-to-end distance of the DNA strand that follows the wormlike chain model from the work of Thirumalai and Ha.(3)

The following figure illustrates the notation utilized for the worm-like chain model. The purple domain represents a spacer, the pale red domain denotes a toehold domain, and the gray domain signifies a displacement domain. The red box indicates a reactive volume where branch migration and strand displacement can be initiated.

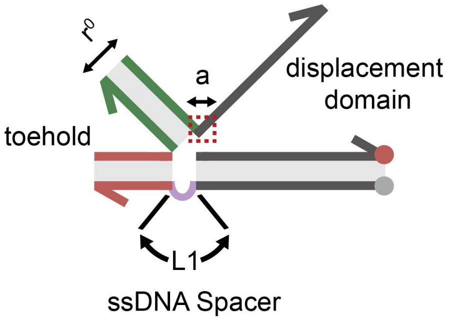

For a blunt-end displacement process, the internal displacement rate constant $k_s$ is simply the rate of the blunt-end reaction multiplied by the effective concentration of displacement domains inside the reaction volume.

$$k_s = k_b c^* \qquad (1)$$

In this equation, $k_b$ represents the branch migration rate constant (0.8 $M^{-1}\cdot s^{-1}$) (4) and $c^*$ is the effective concentration in the red box.

In order to calculate the effective concentration $c^*$, it is necessary to determine the probability that the purple strand is extended to an end-to-end distance $R$ in the range $dR$. The probability distribution $p(R)$ depends on the model used to interpret the DNA biophysics.

Assuming that $p(R)$ is nearly constant within the reaction volume, that is, $a$ is much smaller than the persistence length $s$, the probability $P$ of the chain end being in the reaction volume is:

$$P = p(r_0)a\frac{V_a}{V_s} \qquad (2)$$

Where $V_a = a^3$ is the volume of the red box, $V_s = 4\pi r_0^2 a$ is the volume of the shell of radius $r_0$ and thickness $a$, and $p(r_0)$ is the probability distribution in r0. Considering $a < r_0$, this simplifies to:

$$P = p(r_0)\frac{a^3}{4\pi r_0^2} \qquad (3)$$

Finally, we obtain the effective concentration $c^*$ in $particles/m^3$ by dividing $P$ by the reaction volume $a^3$. To convert the result to $mole/L$, we divide by $1000N_a$, where $N_a$ is Avogadro's number. The effective concentration $c^*$ is given by:

$$c^* = \frac{p(r_0)}{4\pi r_0^2} \times \frac{1}{1000N_a} \qquad (4)$$

We then assume that the reaction rate is as if the duplex containing the target strand saw an invading strand free in solution at a concentration c* and containing only a displacement domain with no toehold.

We proceed to assume the reaction rate as if the complex containing the target chain were to encounter an invading chain that is freely dissolved in solution at a concentration $c^*$ and contained only displacement domains without a toehold.

We now only need the distribution $p(R)$. The DNA chain is modelled as a worm-like chain. The probability $p(R, s, L)dR$ that a worm-like chain of length $L$ and persistence length $s$, to be extended to length $R$ within $dR$ is [Theoretical and Mathematical Models in Polymer Research]:

$$p(R, s, L) = \frac{1}{L} \times \frac{4\pi A r_0^2}{(1-r^2)^{9/2}} exp\left(-\frac{3t}{4(1-r^2)}\right) \qquad (5)$$

$$A = \frac{4(3t/4)^{3/2} exp\,(3t/4)}{\pi^{3/2}\left(4+\frac{12}{3t/4}+\frac{15}{(3t/4)^2}\right)} \qquad (6)$$

where $t$ is the multiple of the contour length $L$ (ssDNA spacer, purple domain) and the persistence length $s$ (2 nm for ssDNA) (5), $t = L/s$; $r$ is the end-to-end distance $R$ normalized to the contour length, $r = R/L$.

The internal displacement rate, $k_s$, can now be calculated according to equation (1).

$$k_s = k_b c^* = k_b \frac{p(r_0)}{4\pi r_0^2} * \frac{1}{1000N_a} \quad (7)$$

Therefore, we can theoretically calculate the reaction rate considering the spacer length $L$ and the shell radius $r_0$.

S1.3 Kinetic modelling of toehold-mediated strand displacement.

The TMSD reaction models were constructed to explore the kinetics of strand displacement reaction via kinetic simulation. The reaction in the TMSD system is as follows:

$$TH + FQ \underset{k_{tr}}{\overset{k_{tf}}{\rightleftarrows}} I_1 \quad (8)$$

$$I_1 \overset{k_S}{\rightarrow} THQ + F \quad (9)$$

The *fourth-order Runge-Kutta* method(6) was employed to numerically solve the following differential equation. Since the differential equation is sensitive to the initial value, it is only necessary to set the corresponding differential terms of each reactant involved in the reaction and the values of the $k_{tf}$, $k_{tr}$, $k_{bf}$, $k_{br}$, $\mathrm{k}_s$and probability of base breathing ($\mathrm{P}_{bre}$ ) involved in the system. As shown in Figure 3, we simulated the overall reaction and analyzed the apparent rate, yield, specificity and analytical performance under different designs. The parameter DDF=Yield $_{(PM)}{}^2$/Yield$_{(MM)}$ is defined, which reflected the specificity and sensitivity of the detection. The heat maps were drawn to represent the different numbers of closures and the corresponding discrimination factors.

Differential equations:

$$\frac{d[TH]}{dt} =- k_{tf}[TH][FQ] + k_{tr}[I_1]$$

$$\frac{d[FQ]}{dt} = \frac{d[TH]}{dt}$$

$$\frac{d[I_1]}{dt} = k_{tf}[TH][FQ] - k_{tr}[I_1]$$

$$\frac{d[THQ]}{dt} = \mathrm{k}_s[I_1]$$

$$\frac{d[F]}{dt} = \frac{d[THQ]}{dt}$$

$$k_{tr} = k_{tf}e^{\frac{-\Delta G}{RT}} \quad (10)$$

In the case of mismatched targets, $\mathrm{k}_{s(MM)} = \mathrm{k}_s \times \mathrm{P}_{bre}$ (11)

$$TH + FQ \underset{k_{tr}}{\overset{k_{tf}}{\rightleftarrows}} I_1$$

(12)

$$\mathrm{I}_1 \underset{\mathrm{k}_{br}}{\overset{\mathrm{k}_{b\mathrm{f}}}{\rightleftarrows}} \mathrm{I}_2 \quad (13)$$

$$\mathrm{I}_2 \overset{\mathrm{k}_S}{\rightarrow} THQ + F$$

(14)

Differential equations:

$$\frac{d[TH]}{dt} =- k_{tf}[TH][FQ] + k_{tr}[I_1]$$

$$\frac{d[FQ]}{dt} = \frac{d[TH]}{dt}$$

$$\frac{d[I_1]}{dt} = k_{tf}[TH][FQ] - k_{tr}[I_1] - \mathrm{k}_{b\mathrm{f}}[I_1] + \mathrm{k}_{br}[I_2]$$

$$\frac{d[I_2]}{dt} = k_{bf}[I_1] - \mathrm{k}_{br}[I_2] - \mathrm{k}_s[I_1]$$

$$\frac{d[THQ]}{dt} = \mathrm{k}_s[I_2]$$

$$\frac{d[F]}{dt} = \frac{d[THQ]}{dt}$$

$$k_{tr} = k_{tf}e^{\frac{-\Delta G}{RT}} \quad (15)$$

$$k_{br} = k_{bf}e^{\frac{-\Delta G}{RT}} \quad (16)$$

In the case of mismatched targets, $\mathrm{k}_{s(MM)} = \mathrm{k}_s \times \mathrm{P}_{bre}$

(17)

## S2. Supplementary Figures

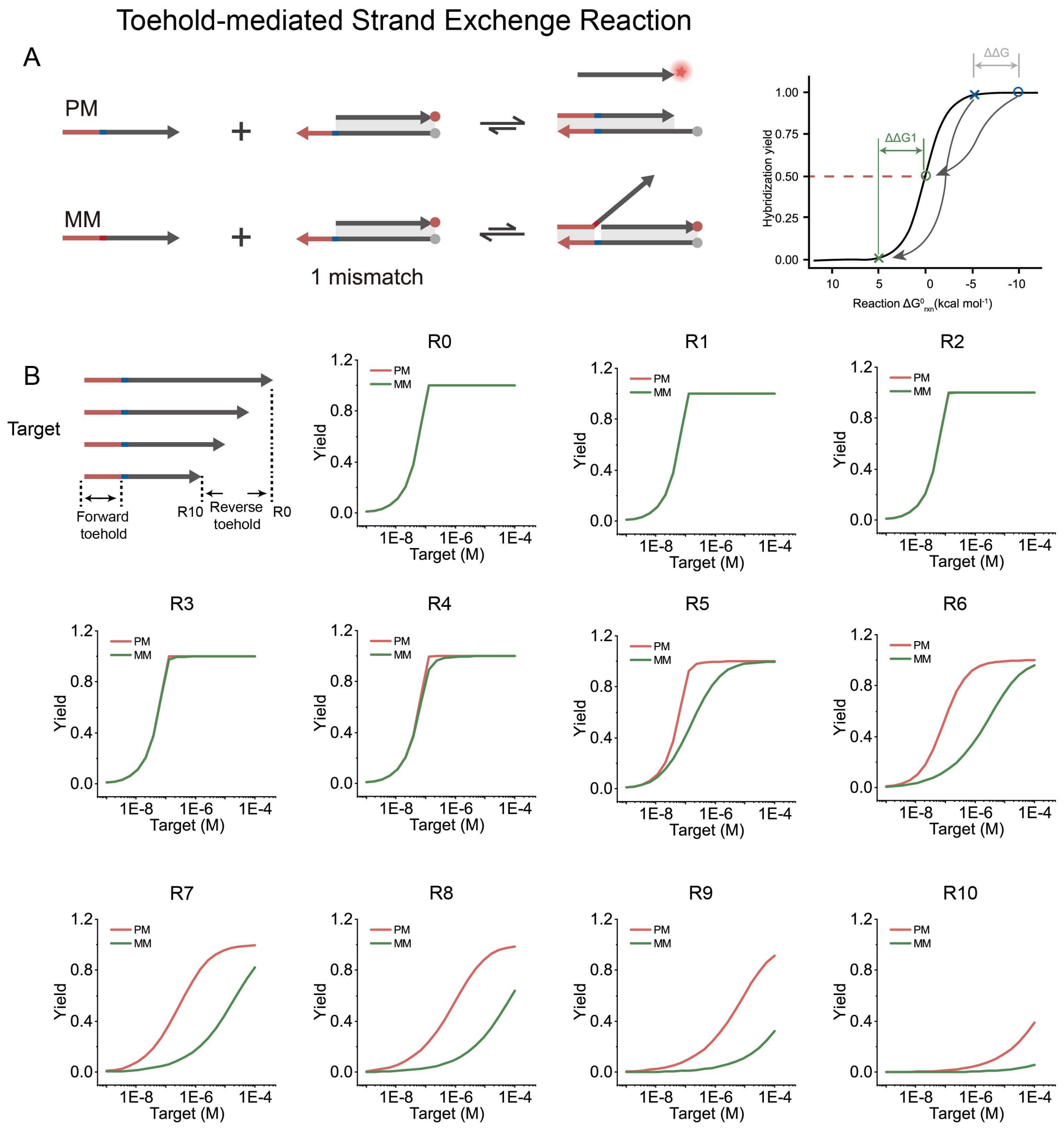


Figure S1. (A) Schematic of the toehold exchange probe for target detection. (B) Simulation of the change in yield with increasing target concentration as the reverse toehold from r = 0 nt to r = 8 nt. PM was the perfect matched target and MM was the mismatched target. The length of the forward toehold is 8 nt, ΔΔG = 2.47 kcal/mol.

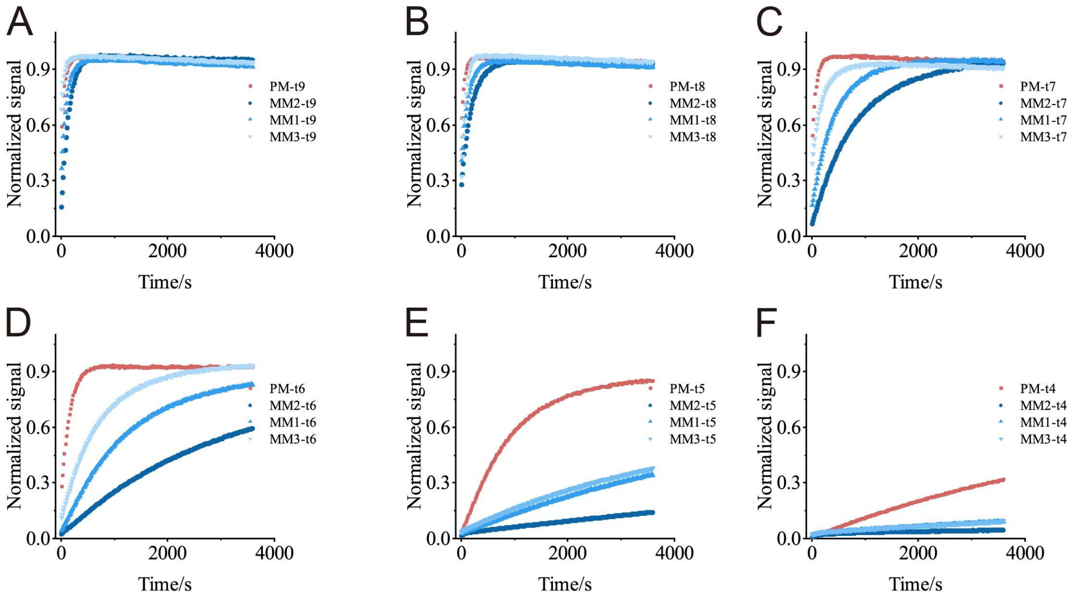


Figure S2. TD probes were used to detect SNVs with toehold lengths of (A) 9 nt, (B) 8 nt (C) 7 nt (D) 6 nt (E) 4 nt (F) 4 nt. MM1 is C mutated to A, MM2 is C mutated to G, and MM3 is C mutated to T.

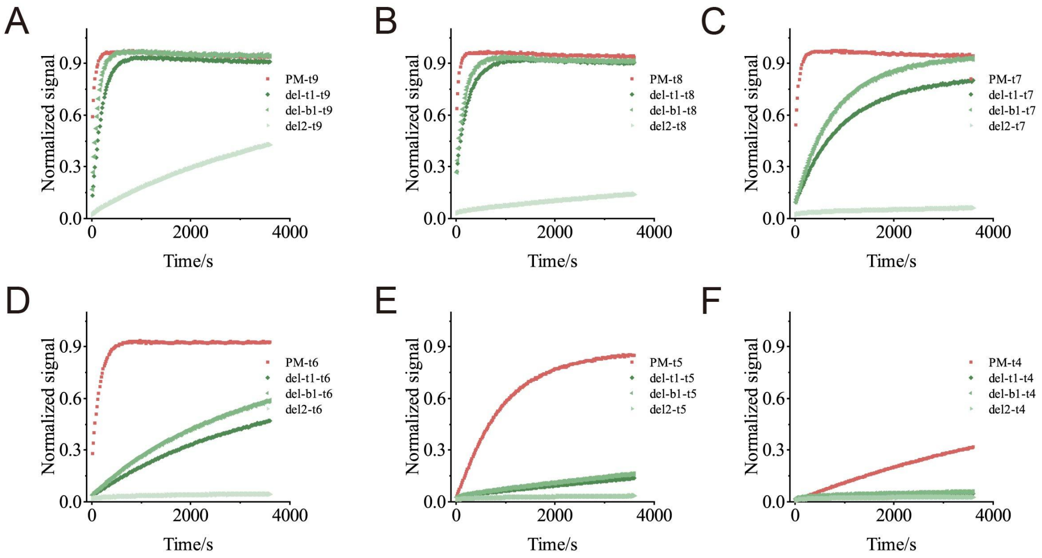


Figure S3. TD probes were used to detect mutant targets with deletions in toehold lengths of (A) 9nt, (B) 8nt (C) 7nt (D) 6nt (E) 4nt (F) 4nt. del-t1 is a deletion of 1nt on the toehold, del-b1 is a deletion of 1nt on the branching migration, and del2 is a deletion of 1nt on each of the toehold and the branch migration.

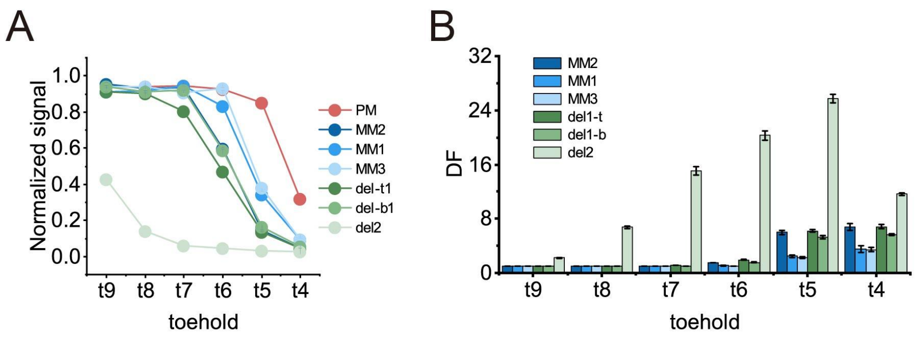


Figure S4. (A) Normalized signal and (B) DF of TD probe detecting mutant targets at different toehold lengths.

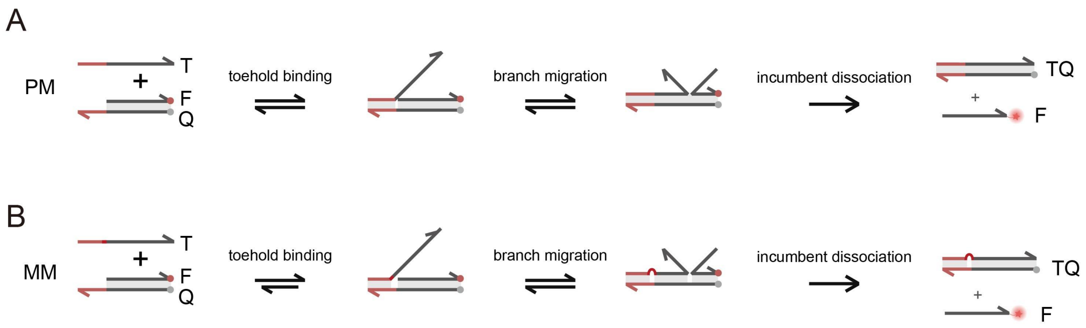


Figure S5. Reaction process of tSDR when (A) the target is PM and (B) the target is MM.

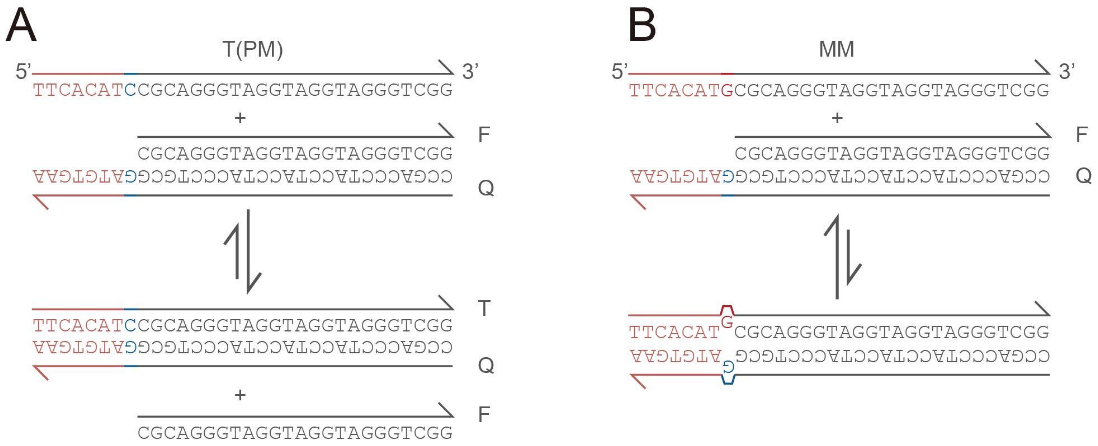


Figure S6. Schematic diagram of tSDR with detailed sequence.

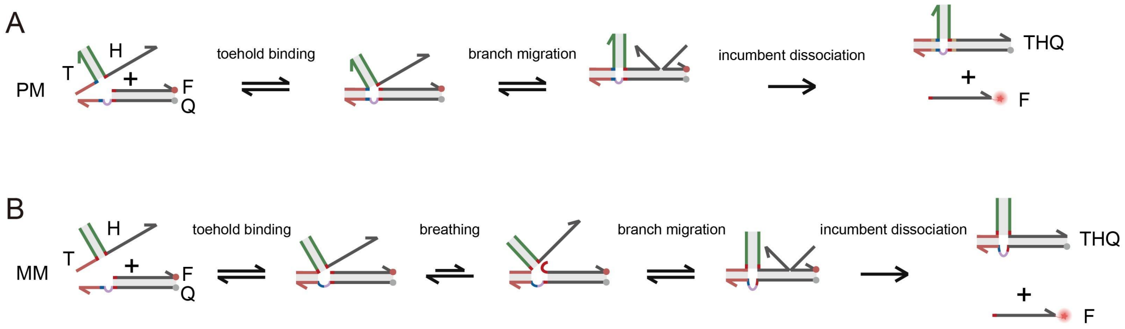


Figure S7. Reaction process of mcSDR when (A) the target is PM and (B) the target is MM.

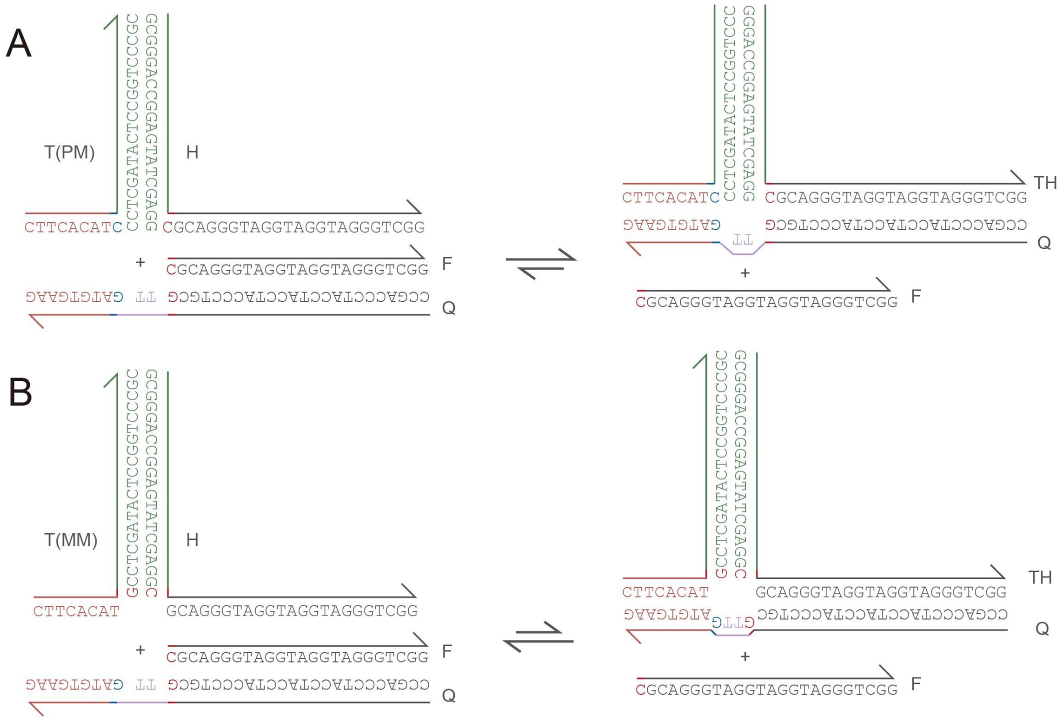


Figure S8. Schematic representation of the mcSDR with detailed sequence.

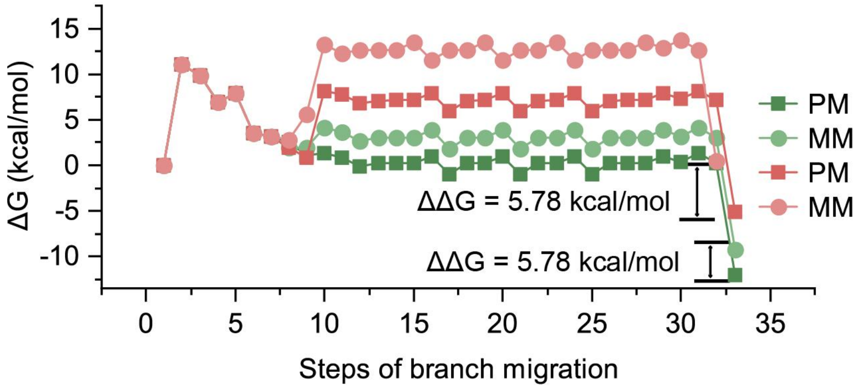


Figure S9. Comparison of energy landscapes. Green represents tSDR, red represents mcSDR.

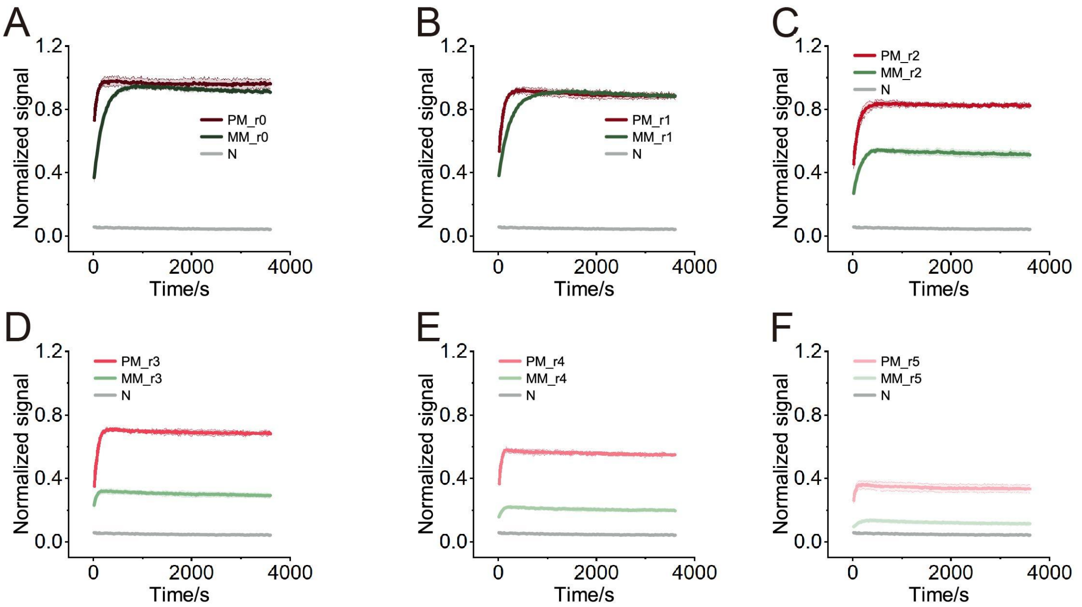


Figure S10. Fluorescence curves when the number of reverse toeholds is (A) 0 nt, (B) 1 nt, (C) 2 nt, (D) 3 nt, (E) 4 nt, and (F) 5 nt. The forward toehold is 8 nt.

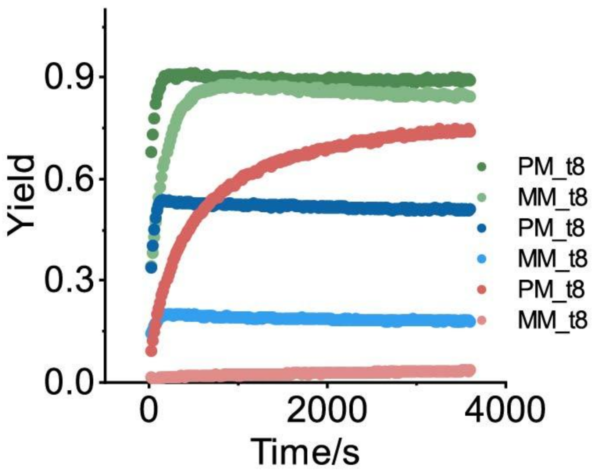

Figure S11. Comparison of the yields of TD (green), TE (blue), and mcSDR (red).

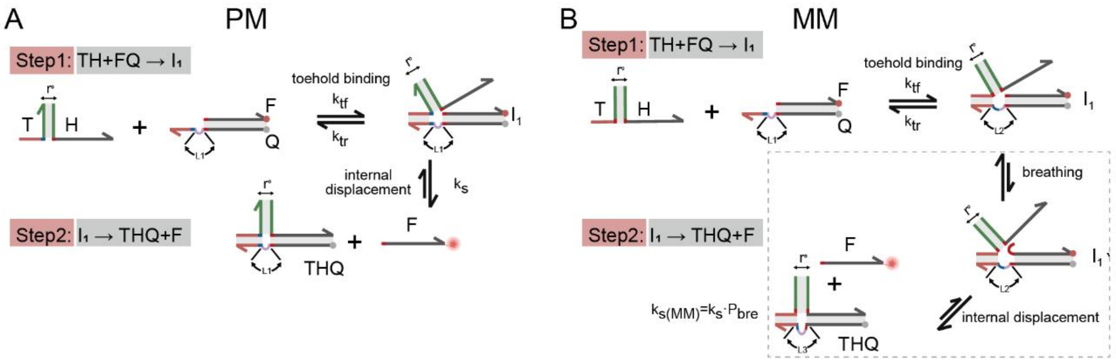


Figure S12. Reaction pathways of (A) PM and (B) MM.

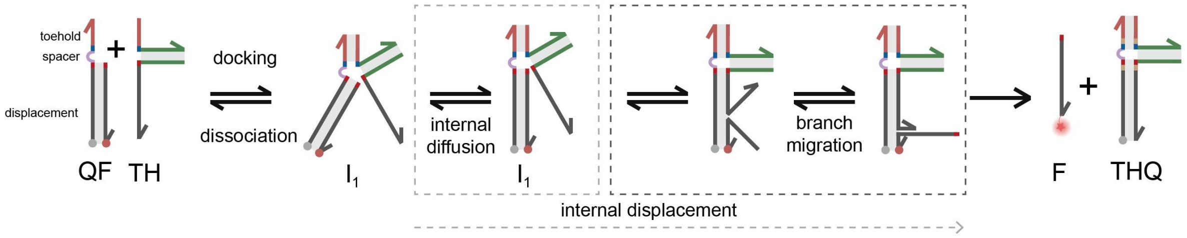


Figure S13. Internal diffusion reaction diagram for mcSDR. The F strand is displaced from the substrate complex (FQ) by the TH strand. After docking the substrate and target via hybridization of the toehold domains, internal diffusion is required to align the displacement domains and initiate the branch migration reaction. The target strand is then displaced from the substrate and the reporter strand regains fluorescence and reports the displacement reaction.

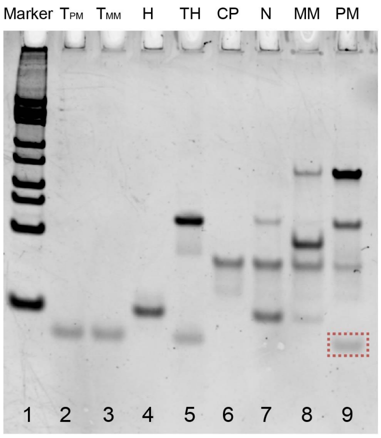


Figure S14. PAGE analysis of mcSDR. Lanes 2 and 3 are PM and MM, respectively. Lane 4 is the helper strand. Lane 5 is the complex of target and helper strand. Lanes 6 is the probe. Lane 7 is the mcSDR reaction without the target. Lane 8 is the MM-triggered mcSDR. Lane 9 is the PM-triggered mcSDR.

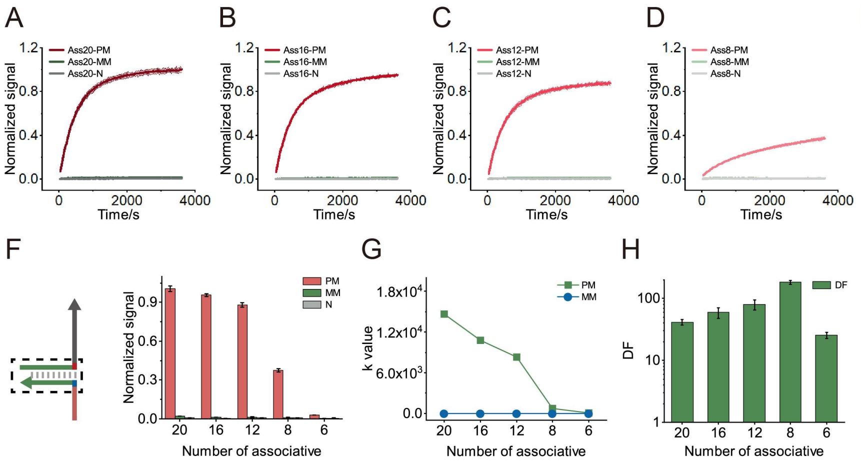


I

| Stem (nt) | k ($s^{-1}M^{-1}$) | -ΔG (kcal/mol) |
|---|---|---|
| 6 | 41.6 | -9.64 |
| 8 | 763.8 | -11.10 |
| 12 | 8277.9 | -17.16 |
| 16 | 10757.9 | -23.76 |
| 20 | 14630.8 | -31.85 |

Figure S15: Real-time fluorescence curves of PM and MM-triggered mcSDR with association domain lengths of (A) 20 nt, (B) 16 nt, (C) 12 nt, (D) 8 nt and (E) 6 nt. (F) Comparison of the normalized signals generated under different association domain lengths. (G) Rates constant of reactions at different association domain lengths. (H) DF of the reaction at different association domain lengths. (I) Predicted ΔG values and determined k values at different association domain lengths.

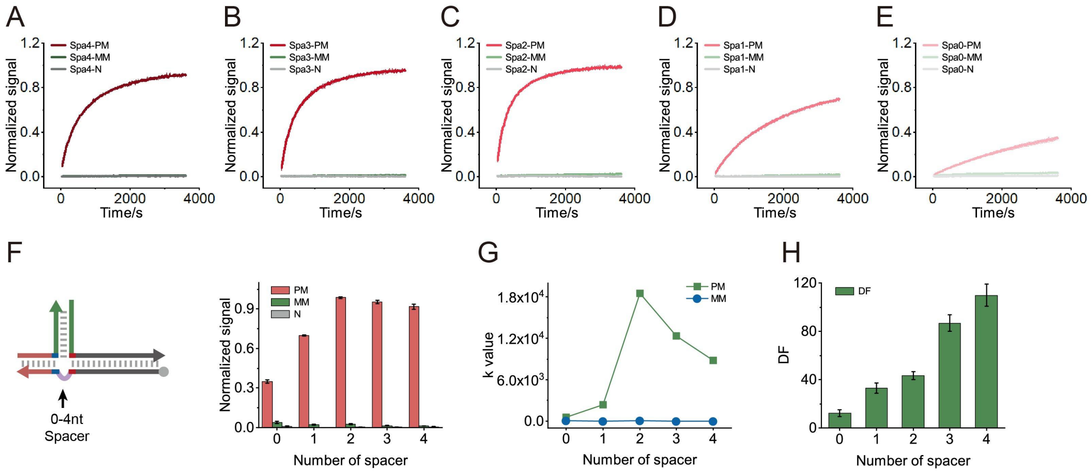


Figure S16. Real-time fluorescence curves of mcSDR triggered by PM and MM at (A) 4 nt, (B) 3 nt, (C) 2 nt, (D) 1 nt, and (E) 0 nt spacers. (F) Comparison of normalized signals with different numbers of spacers. (G) Rates constant of reactions with different numbers of spacers. (H) DF of the reaction with different numbers of spacers.

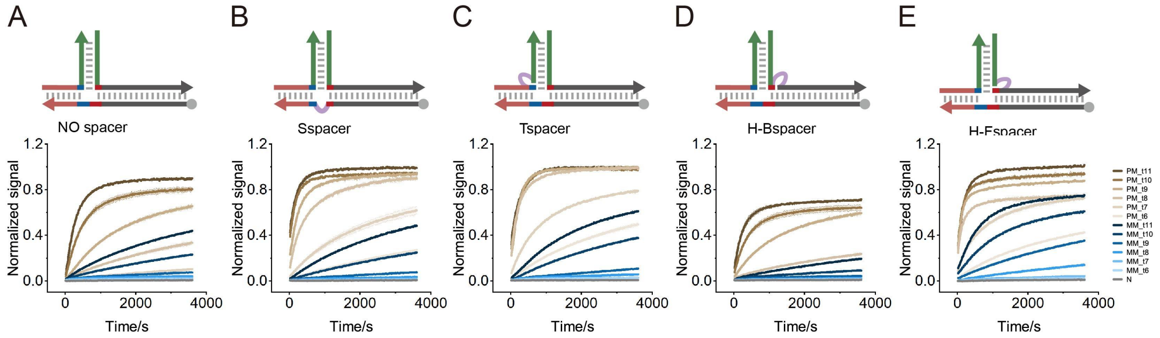


Figure S17. Real-time fluorescence curves of mcSDR triggering by PM and MM for (A) no spacer, spacer on (B) substrate, (C) target, (D) back end of closed base on helper strand, and (E) front end of closed base on helper strand.

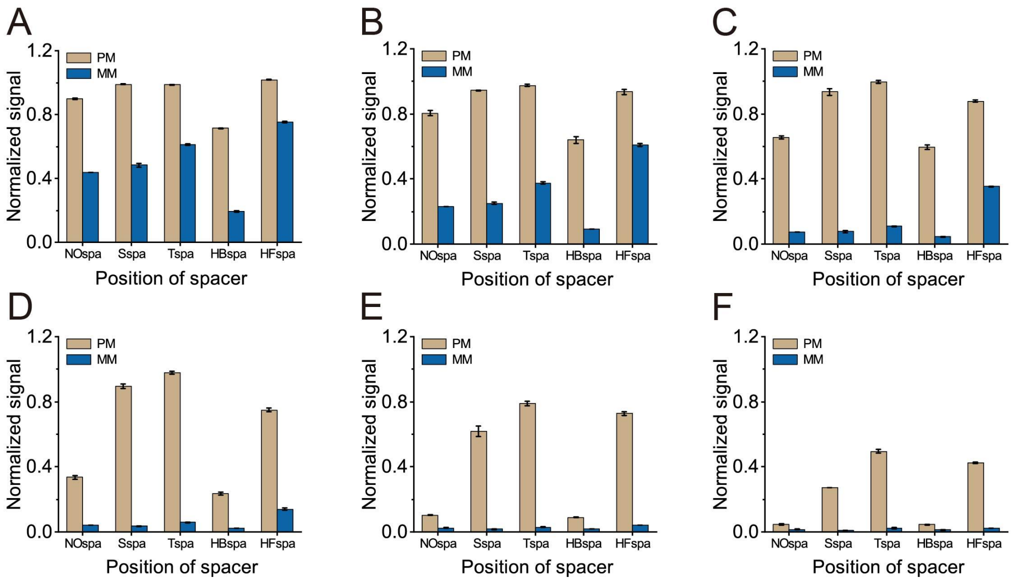


Figure S18. Comparison of normalized signals generated at different spacer positions for toehold lengths of (A) 11nt, (B) 10nt, (C) 9nt, (D) 8nt, (E) 7nt, and (F) 6nt.

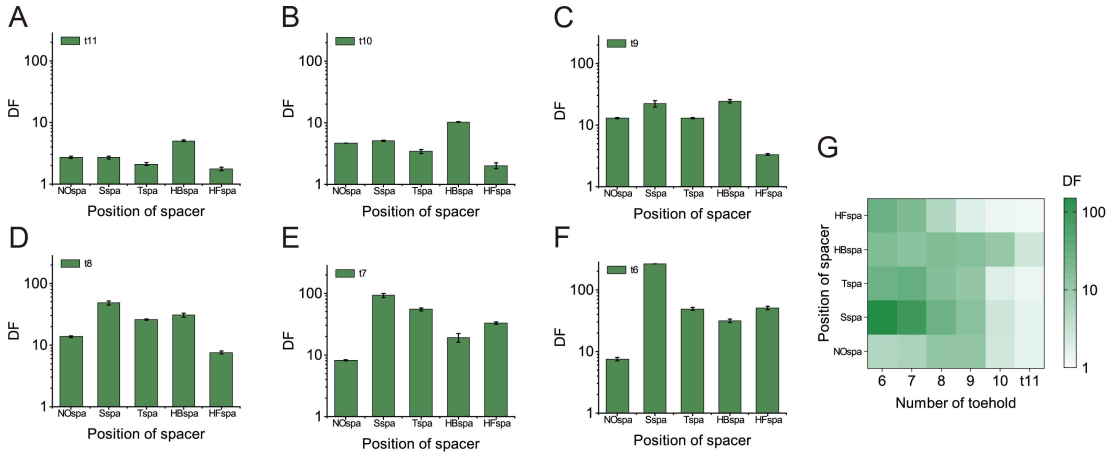


Figure S19. DF of the reaction at different spacer positions for toehold lengths of (A) 11 nt, (B) 10 nt, (C) 9 nt, (D) 8 nt, (E) 7 nt, and (F) 6 nt.(G) Heatmap demonstrating the DF at different toehold lengths and different spacer positions.

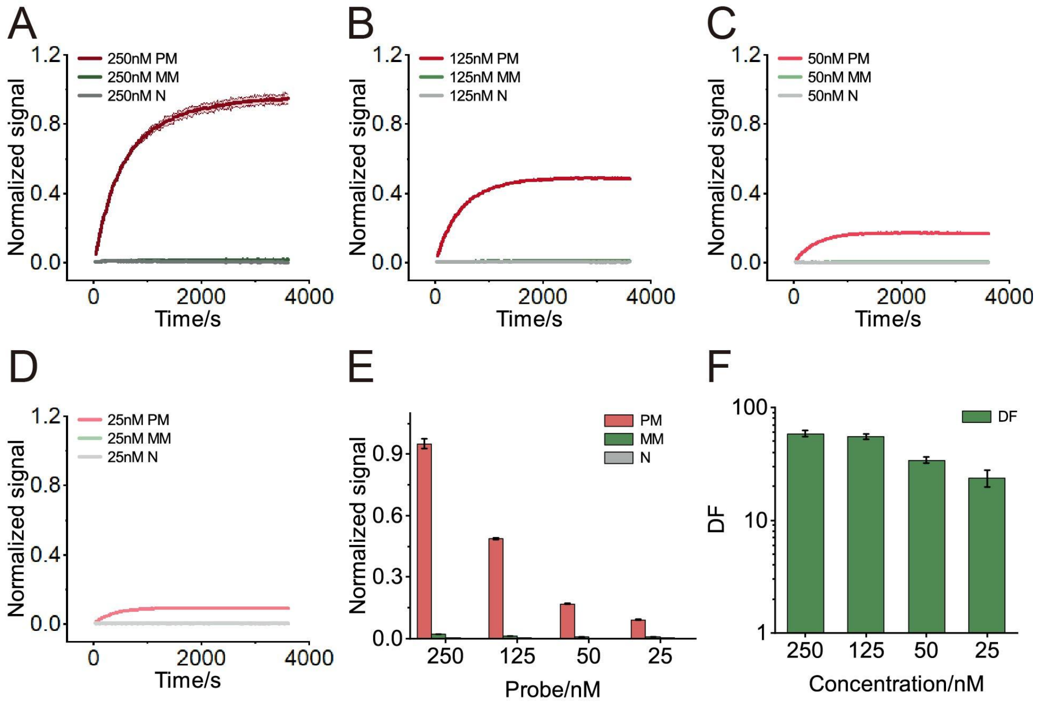


Figure S20. Real-time fluorescence curves of mcSDR triggered by PM and MM at probe concentrations of (A) 250 nM, (B) 125nM, (C) 50 nM and (D) 25 nM. (E) Comparison of normalized signals generated at different probe concentrations. (F) DF of the reaction under different lengths of association domains.

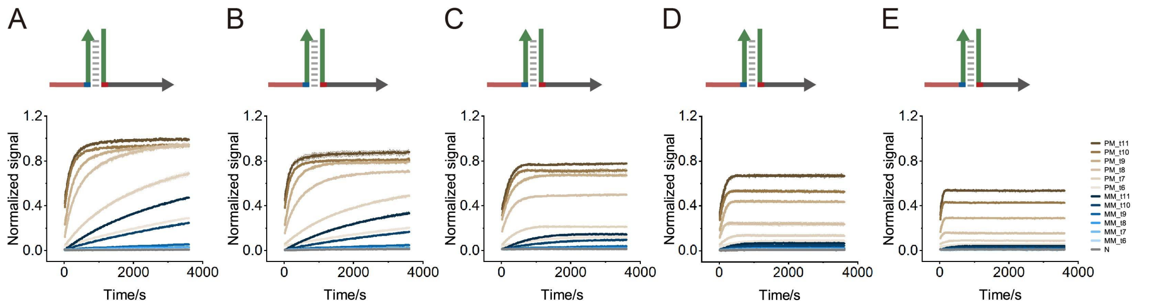


Figure S21. Real-time fluorescence curves of PM and MM-triggered mcSDR for reverse toehold lengths of (A) 0 nt, (B) 1 nt, (C) 2 nt, (D) 3 nt, and (E) 4 nt.

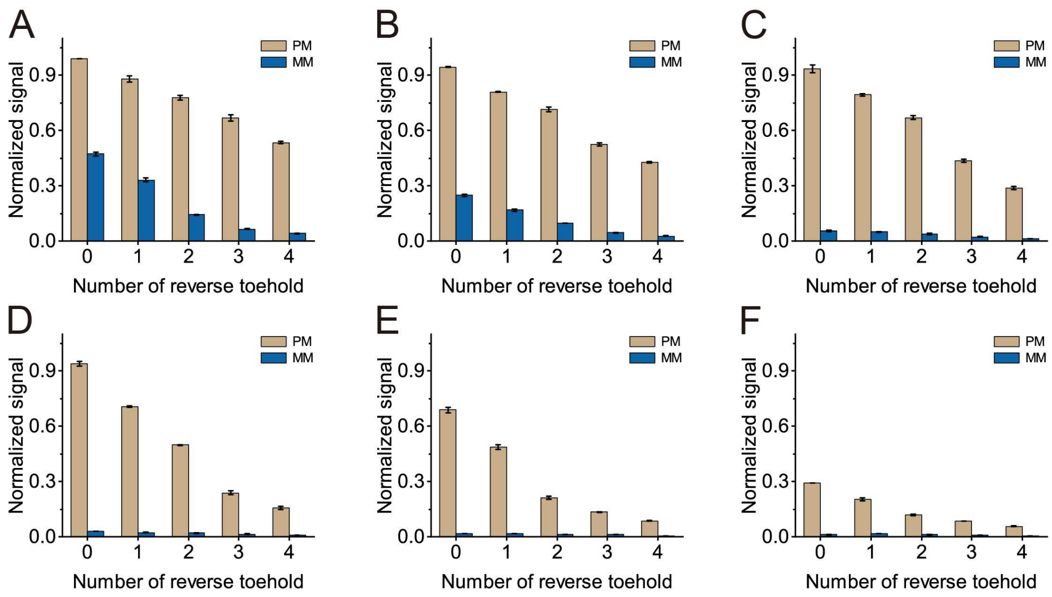

Figure S22. Comparison of normalized signals generated at different numbers of reverse toeholds when the forward toehold length is (A) 11 nt, (B) 10 nt, (C) 9 nt, (D) 8 nt, (E) 7 nt, and (F) 6 nt.

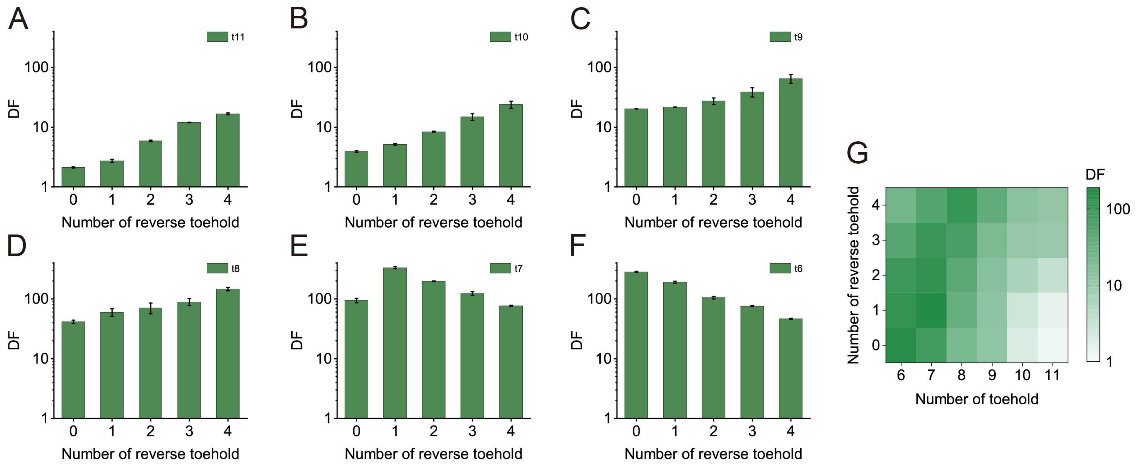


Figure S23. DF of the reaction with different numbers of reverse toeholds when the forward toehold length is (A) 11nt, (B) 10nt, (C) 9nt, (D) 8nt, (E) 7nt, and (F) 6nt. (G) Heat map showing the DF of the reaction with different lengths of forward toehold and different lengths of reverse toehold.

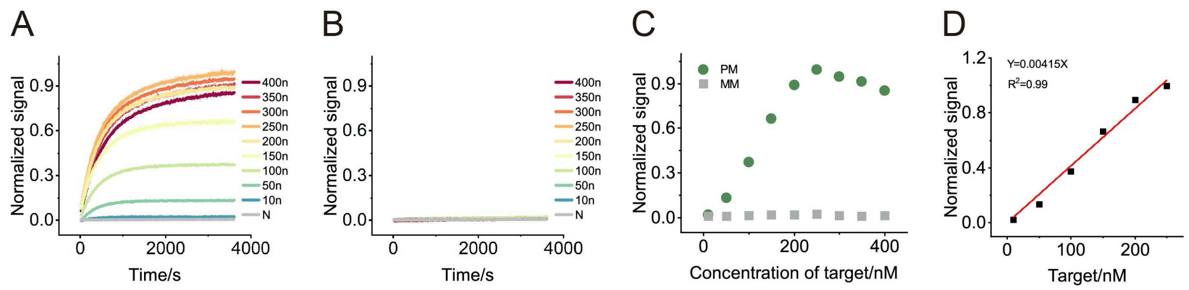


Figure S24. Real-time fluorescence curves of mcSDR triggered by different concentrations of (A) PM and (B) MM. (C) mcSDR detection of targets in different concentration ranges. (D) linear fitting of normalized signal signals and target concentrations in the concentration range of 10 nM-250 nM.

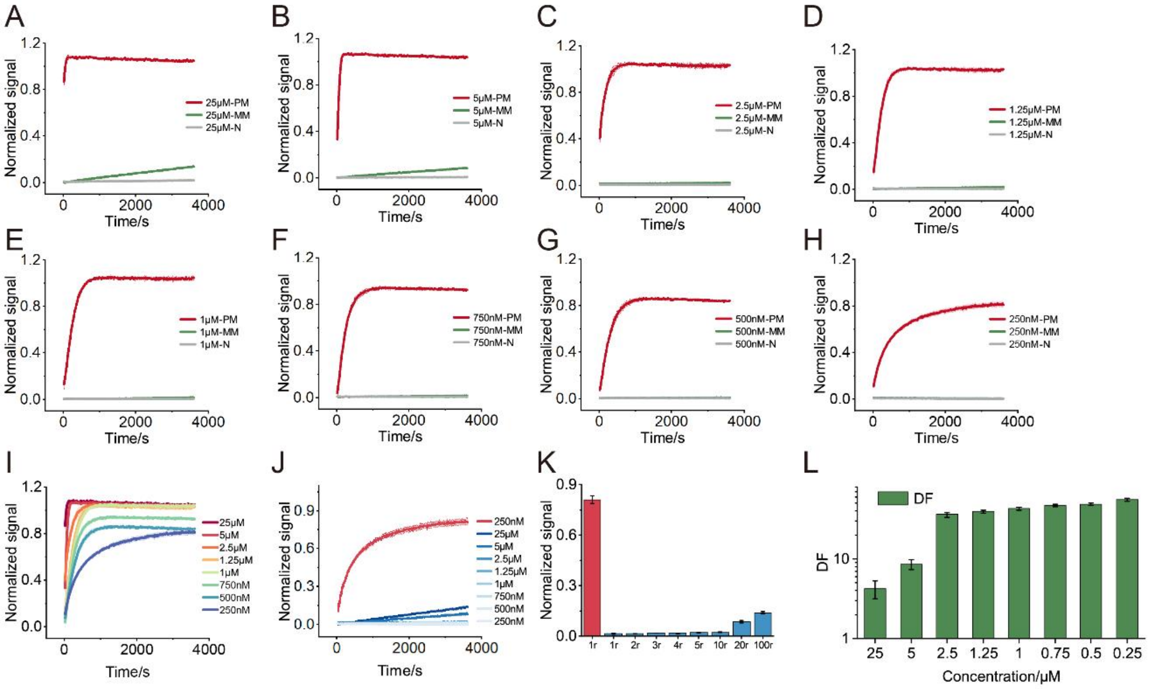

Figure S25. Real-time fluorescence curves of PM and MM-triggered mcSDR at target concentrations of (A) 25 μM, (B) 5 μM, (C) 2.5 μM, (D) 1.25 μM, (E) 1 μM, (F) 750 nM, (G) 500 nM, and (H) 250 nM. (I) Comparison of normalized signals generated by different concentrations of PM. (J) Fluorescence signals generated by 250nM PM and different concentrations of MM. Red is PM, blue is MM.(K) Comparison of normalized signals from the t = 3600s time point of Figure S25J. (L) DF of high concentration targets.

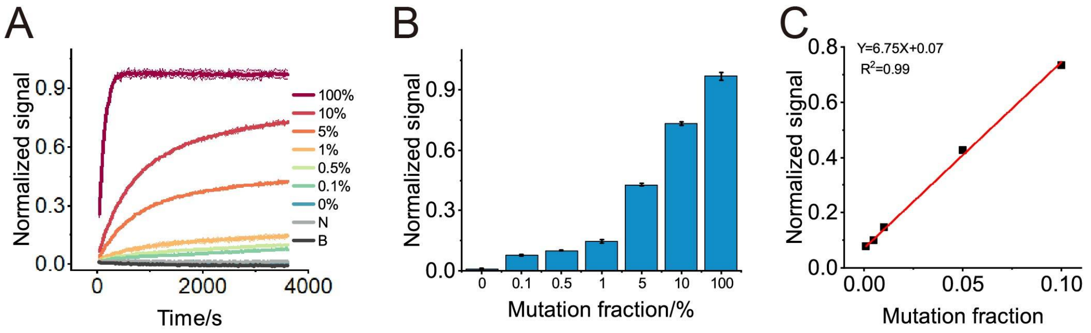

Figure S26. (A) Fluorescence normalized signals generated by different abundance targets. (B) Comparison of normalized signals from the t = 3600s time point of Figure S26A. (C) Linear fit of normalized signal fluorescence to target concentrations of different abundances.

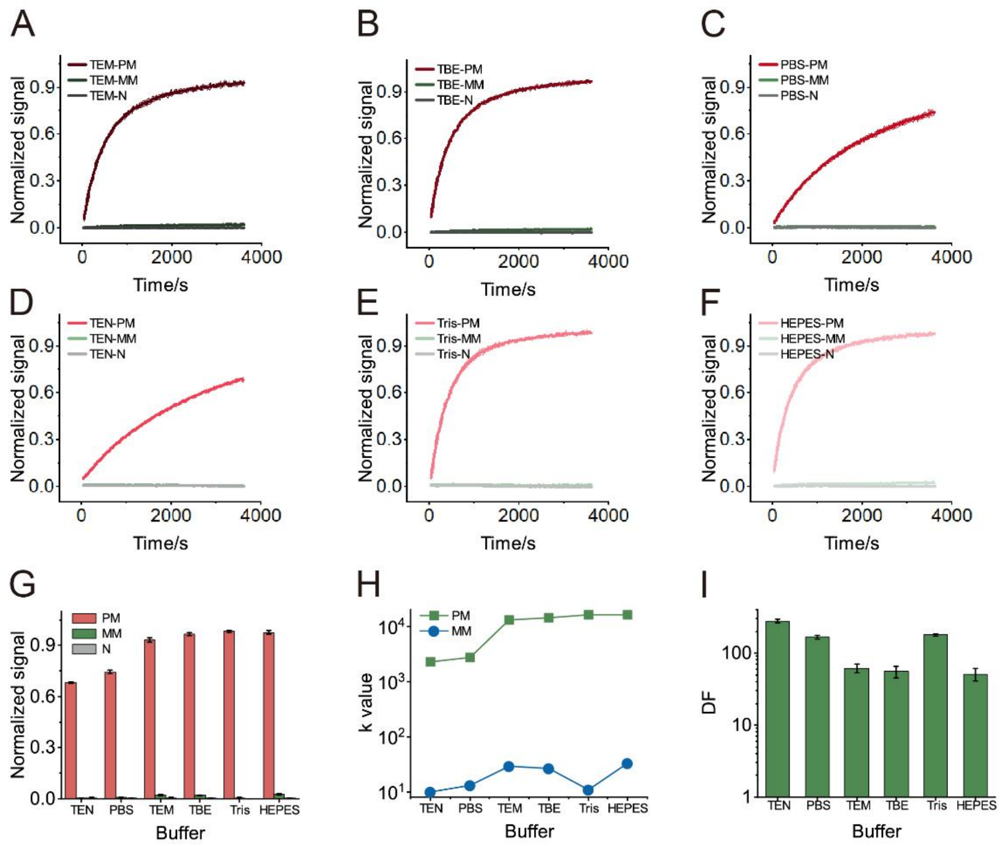


Figure S27. The real-time fluorescence curves of PM and MM triggered mcSDR when the reaction buffer was (A) TEM, (B) TBE, (C) PBS, (D) TEN, (E) Tris and (F) HEPES. (G) Comparison of normalized signals generated under different buffers. (H) The rates constant of the reaction under different buffers. (I) DF of the reaction under different buffers.

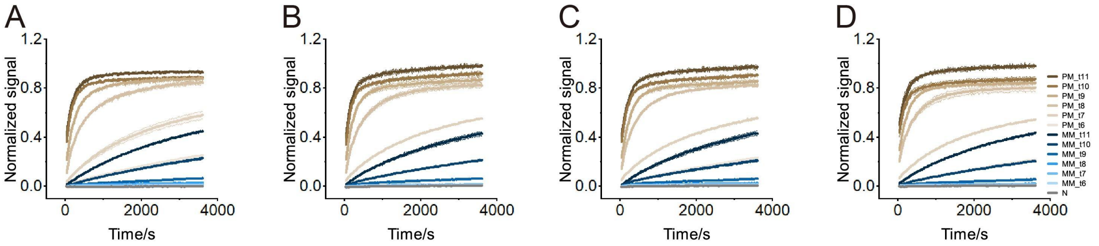


Figure S28: Real-time fluorescence curves of PM and MM triggered mcSDR at reaction temperatures of (A) 37°C, (B) 45°C, (C) 52°C, and (D) 60°C.

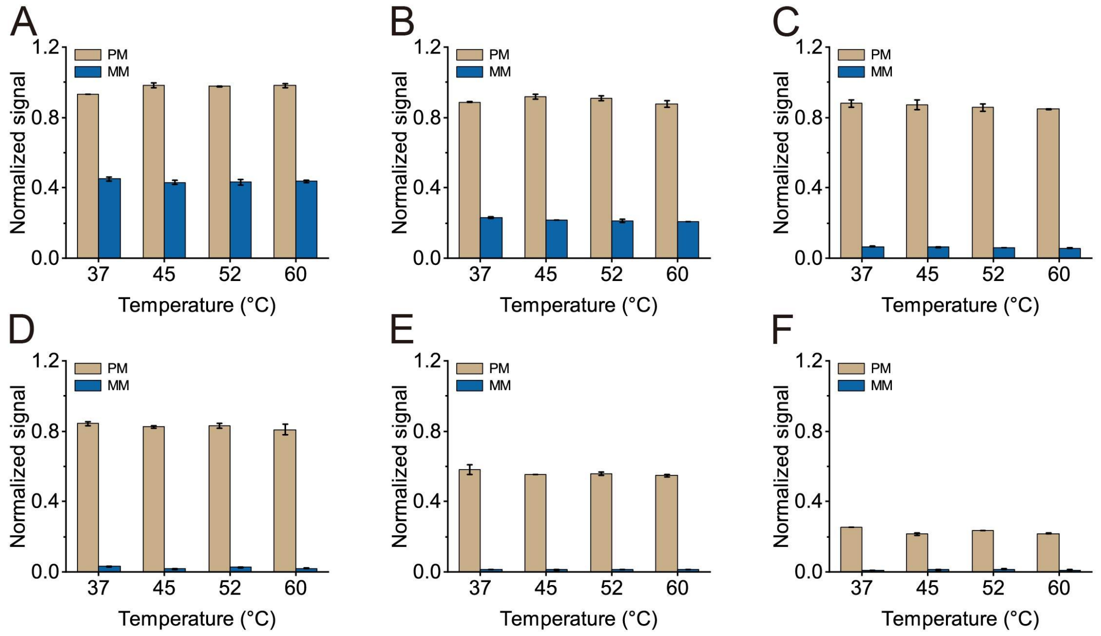


Figure S29. Comparison of normalized signals generated at different temperatures for toehold lengths of (A) 11nt, (B) 10nt, (C) 9nt, (D) 8nt, (E) 7nt, and (F) 6nt.

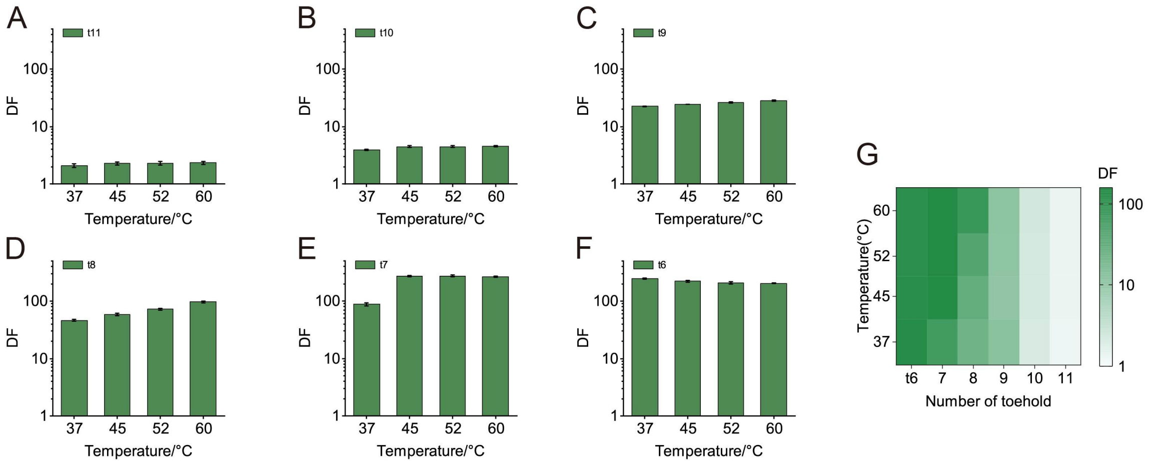


Figure S30. DF of the reaction at different temperatures for toehold lengths of (A) 11 nt, (B) 10 nt, (C) 9 nt, (D) 8 nt, (E) 7 nt, and (F) 6 nt. (G) Heatmap demonstrating the DF at different toehold lengths and different temperatures.

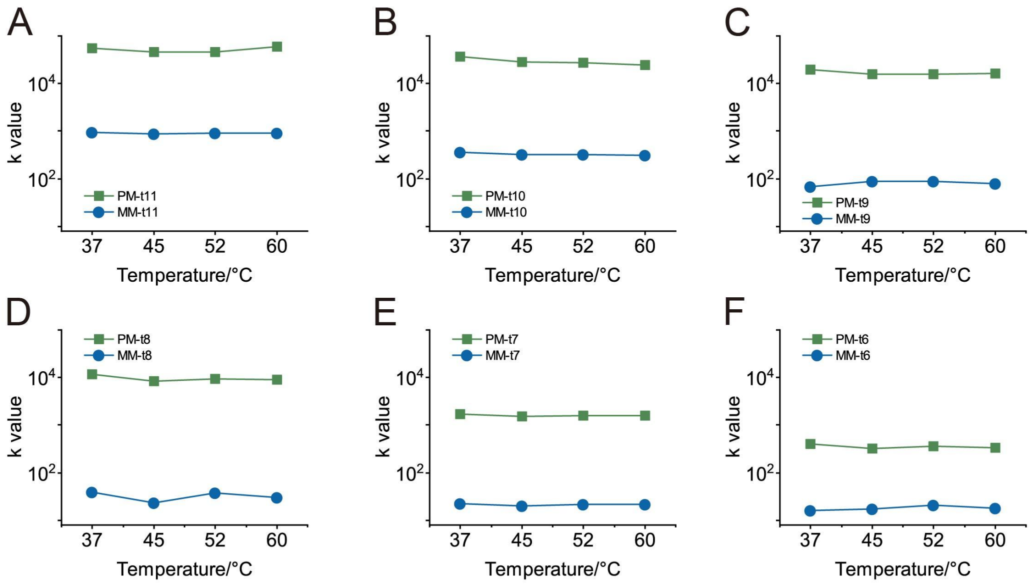


Figure S31. The rates constant of reactions at different temperatures for toehold lengths of (A) 11 nt, (B) 10 nt, (C) 9 nt, (D) 8 nt, (E) 7 nt, and (F) 6 nt.

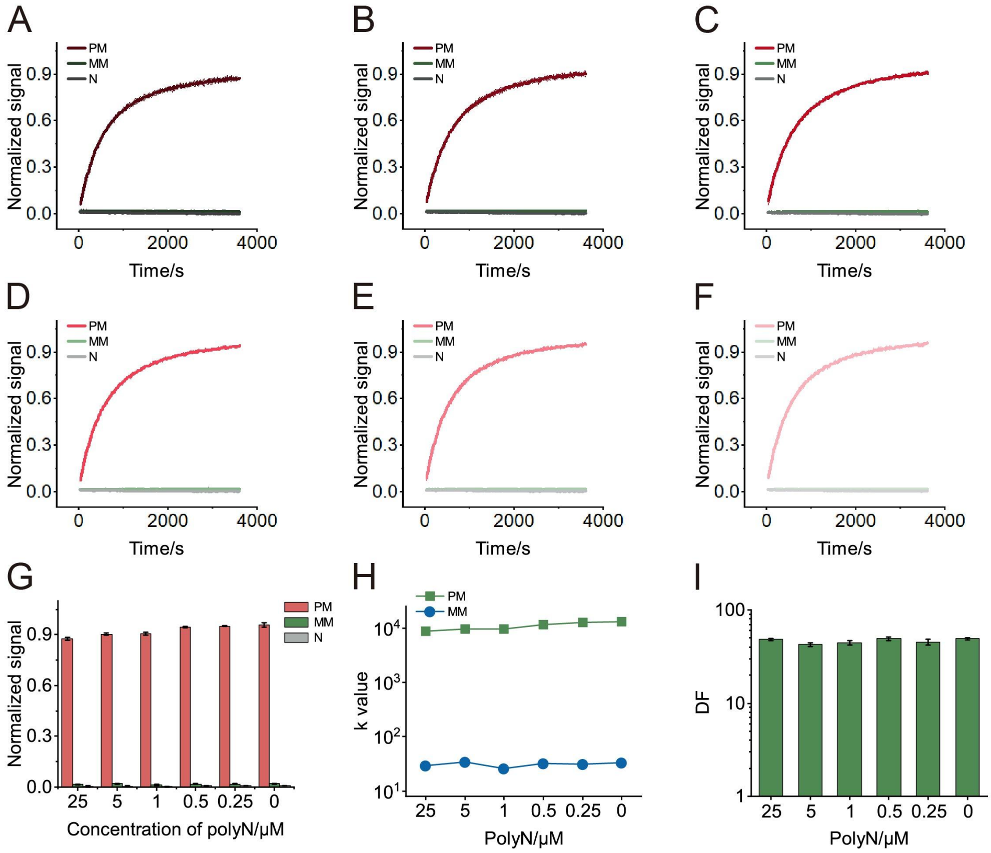


Figure S32. Real-time fluorescence curves of PM and MM triggered mcSDR at interference strand PolyN concentrations of (A) 25 μM, (B) 5 μM, (C) 1 μM, (D) 500 nM, (E) 250 nM and (F) 0 nM. (G) Comparison of normalized signals generated at different PolyN concentrations. (H) The rate constant of the reaction at different PolyN concentrations. (I) DF of the reaction at different PolyN concentrations.

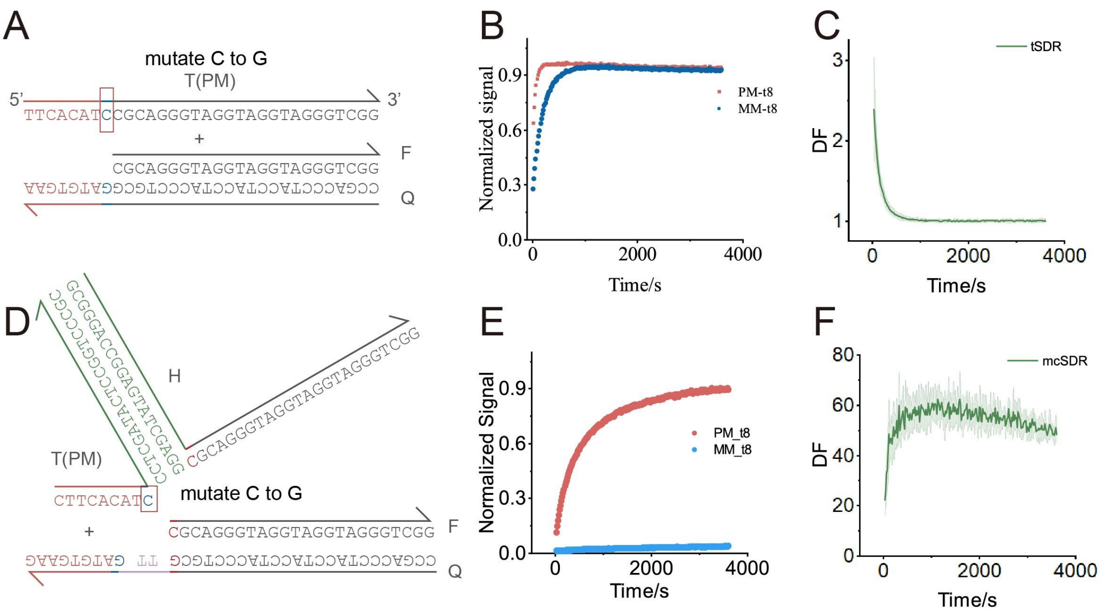


Figure S33. (A) Sequence and (B) real-time fluorescence curve of tSDR reactions. (C) tSDR after the reaction with a gradual decrease in DF. (D) Sequence and (E) real-time fluorescence curve of mcSDR reaction. (F) mcSDR after reaction, with a gradual increase in DF and reaching equilibrium within 1000s.

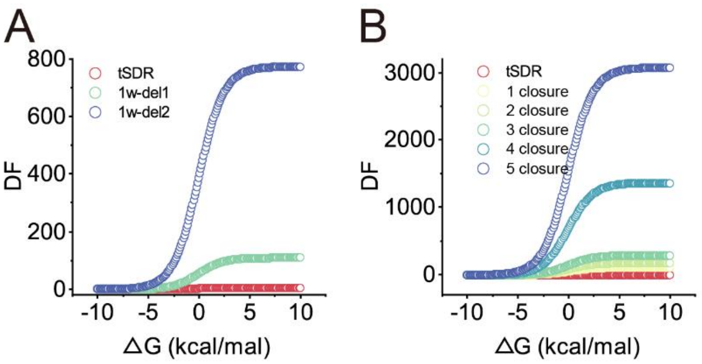


Figure S34. The curve of DF versus ΔG when(A) tSDR and (B) mcSDR with different numbers of closures is used for mutation detection.

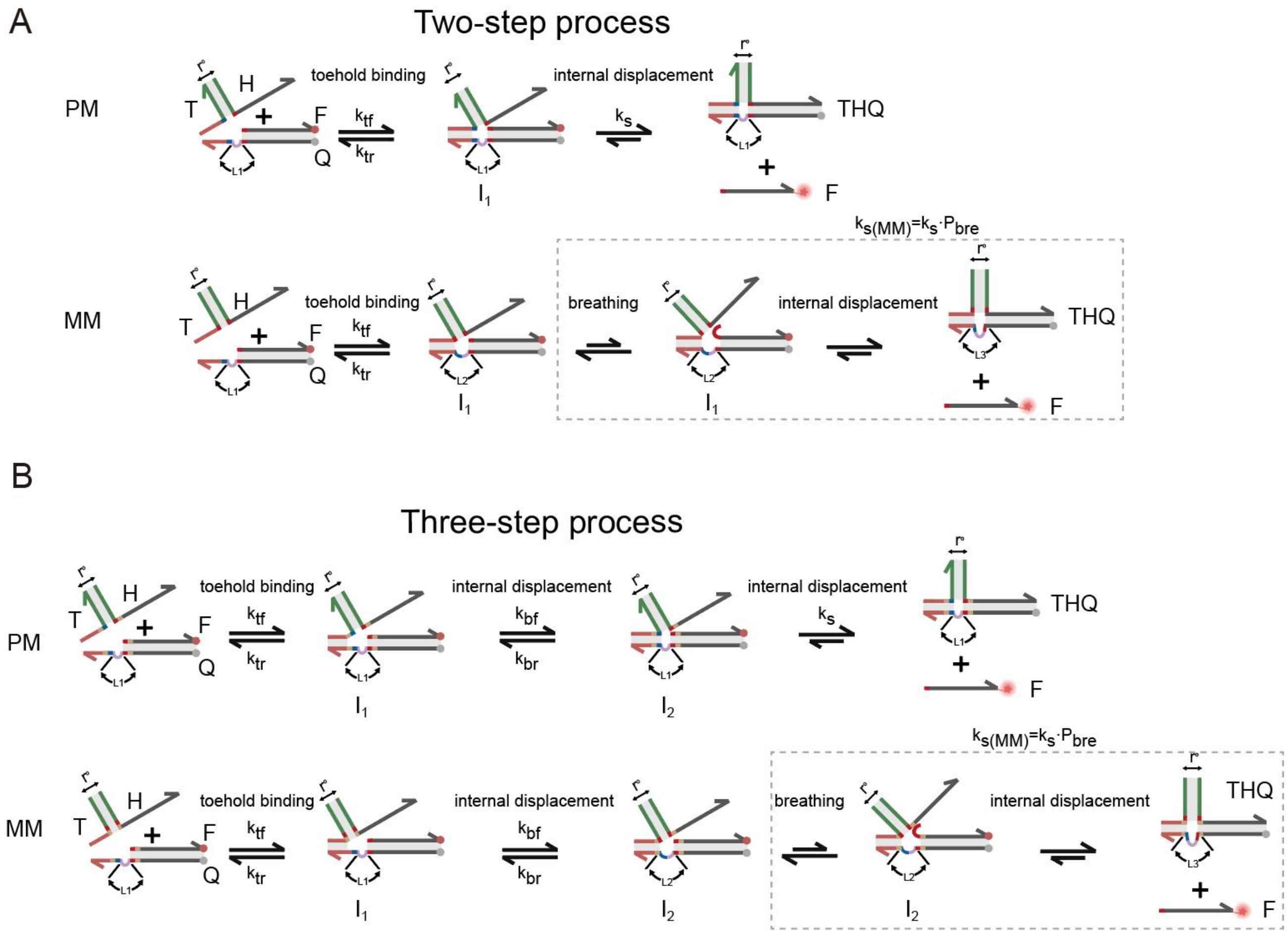


Figure S35. (A) Two-step method for analyzing the mcSDR when 1 base is enclosed. (B) Three-step method for analyzing the mcSDR when more than 2 bases are enclosed.

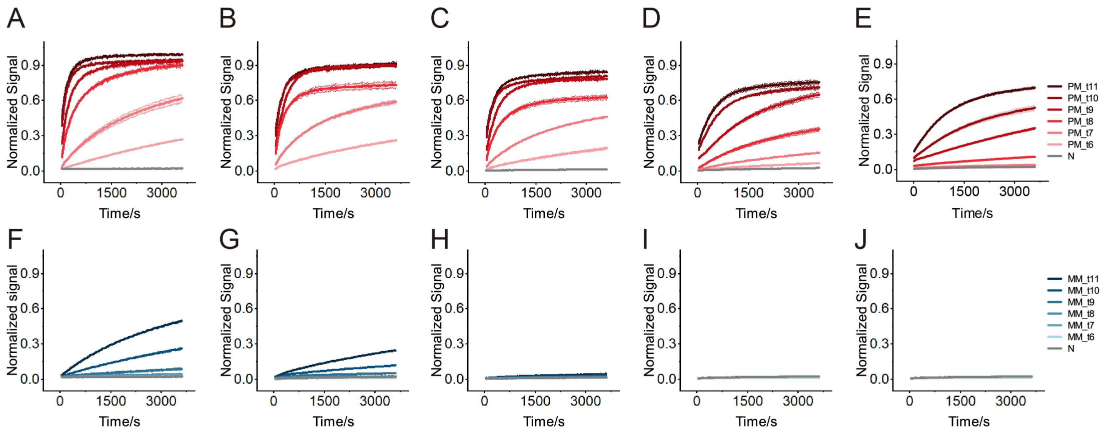


Figure S36. Real-time fluorescence curves when targets were (A-E) PM and (F-J) MM with 1-5 closed bases.

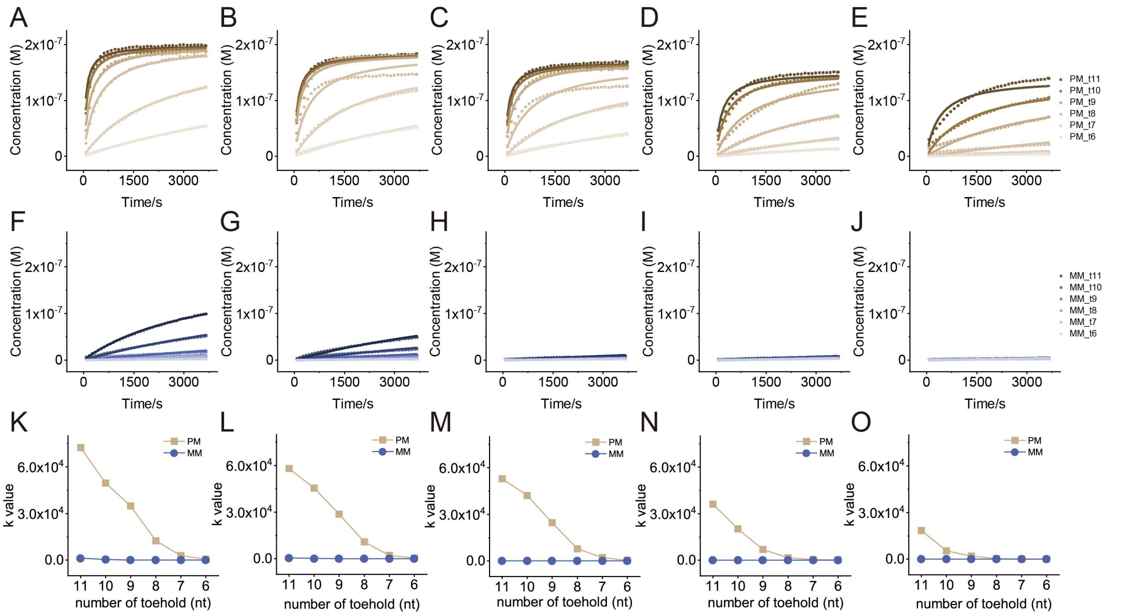


Figure S37. Rate constant when the targets are (A-E) PM and (F-J) MM were (A-E) PM and (F-J) MM with 1-5 closed bases. The solid line indicates the best fit to the kinetic model. (K-O) Rate constant comparison when the number of closed bases is 1-5. The error line indicates the standard deviation of the three measurements.

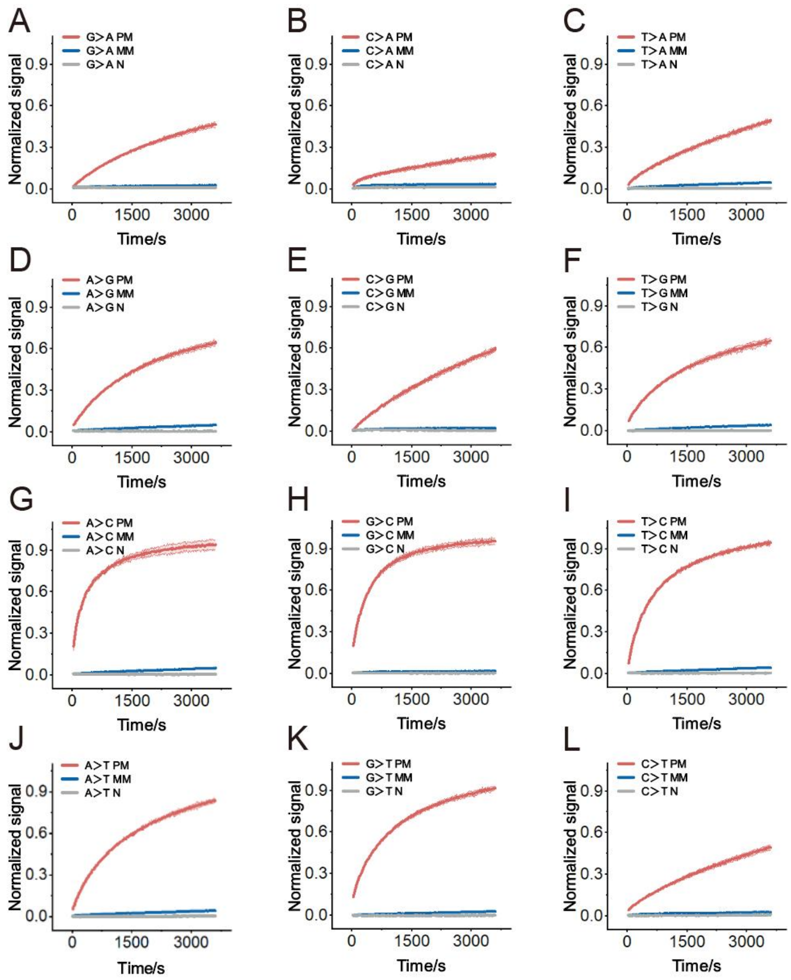


Figure S38. Real-time fluorescence curves when closing one base of mcSDR (αmcSDR) to detect different mutation types.

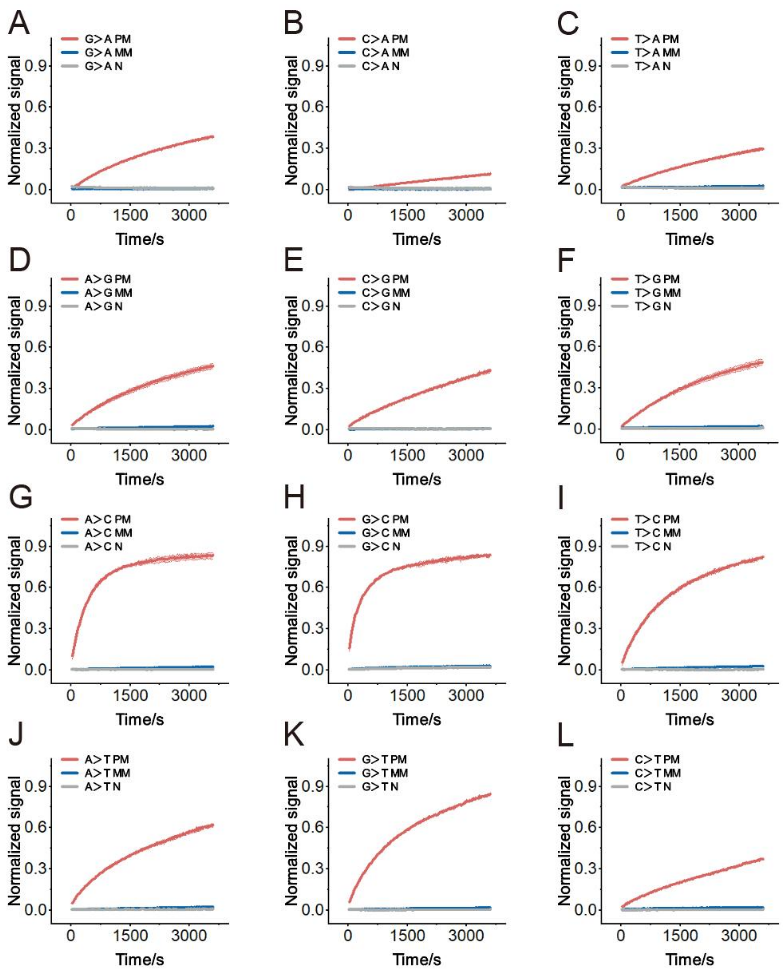


Figure S39. Real-time fluorescence curves when closing 2 bases of mcSDR (γmcSDR) to detect different mutation types.

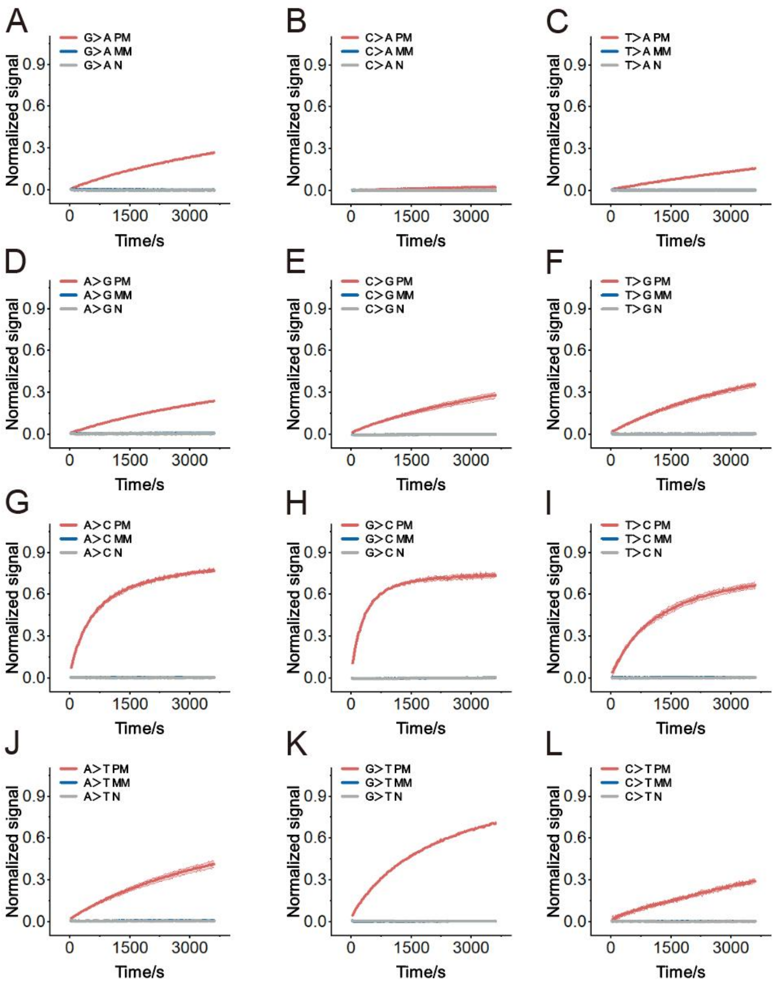


Figure S40. Real-time fluorescence curves when closing 3 bases of mcSDR (εmcSDR) to detect different mutation types.

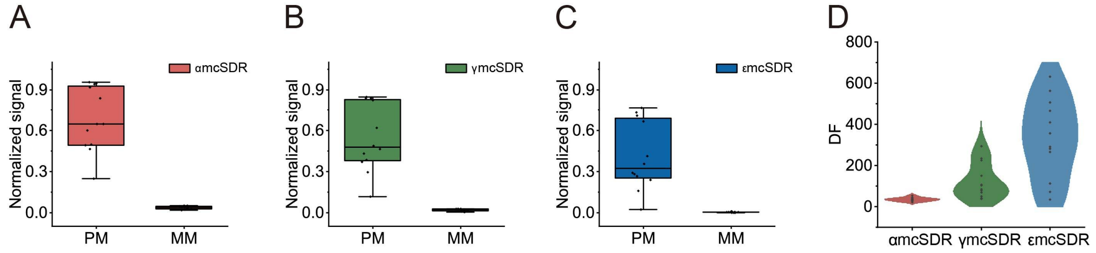


Figure S41. Comparison of the normalized signals of PM and MM when the mcSDR is closed for (A) 1 nt, (B) 2 nt, and (C) 3 nt. (D) Comparison of DF produced by targets with different mutation types at different closure numbers.

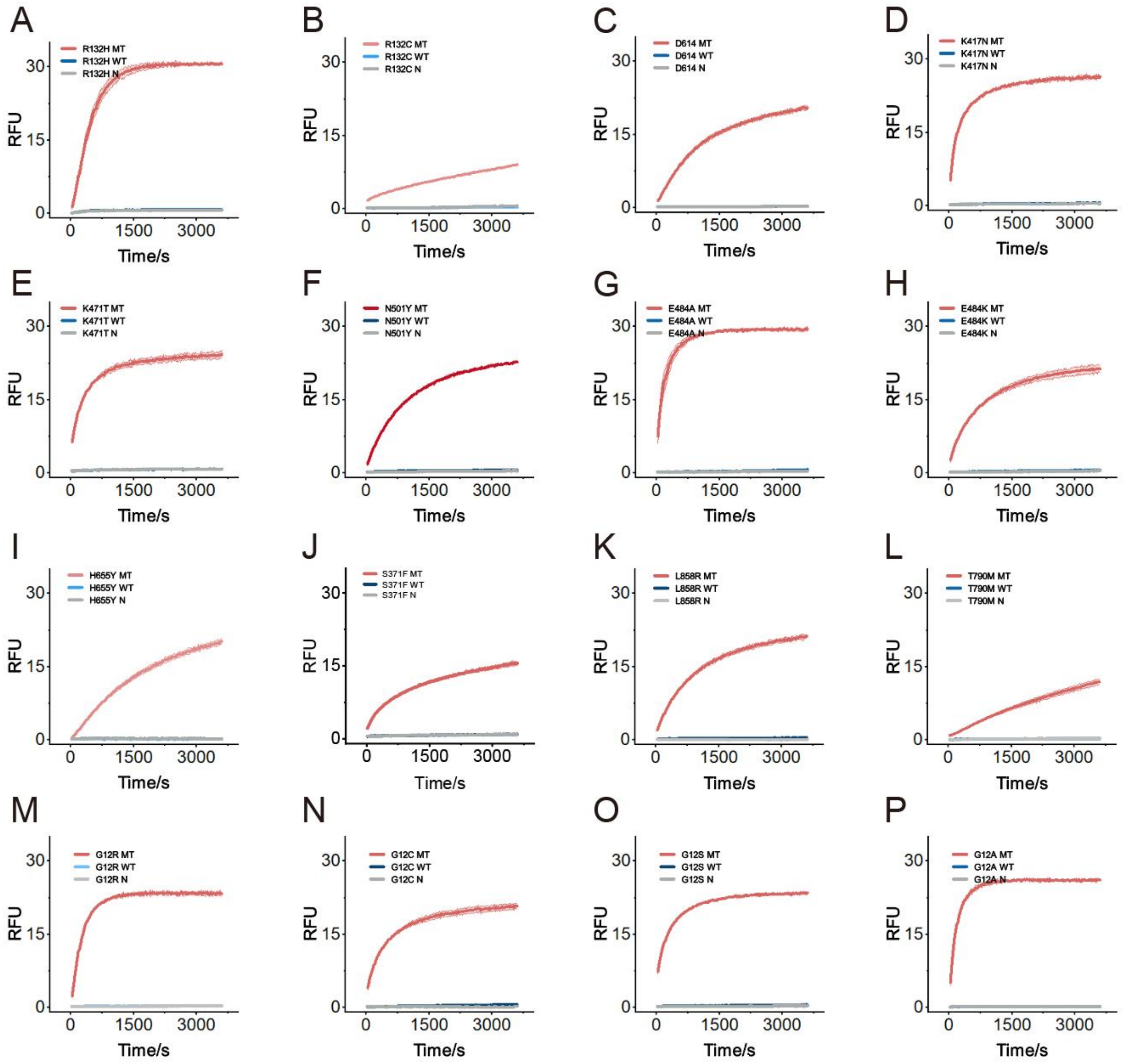


Figure S42. The part of the real-time fluorescence curve corresponding to Figure 4G.

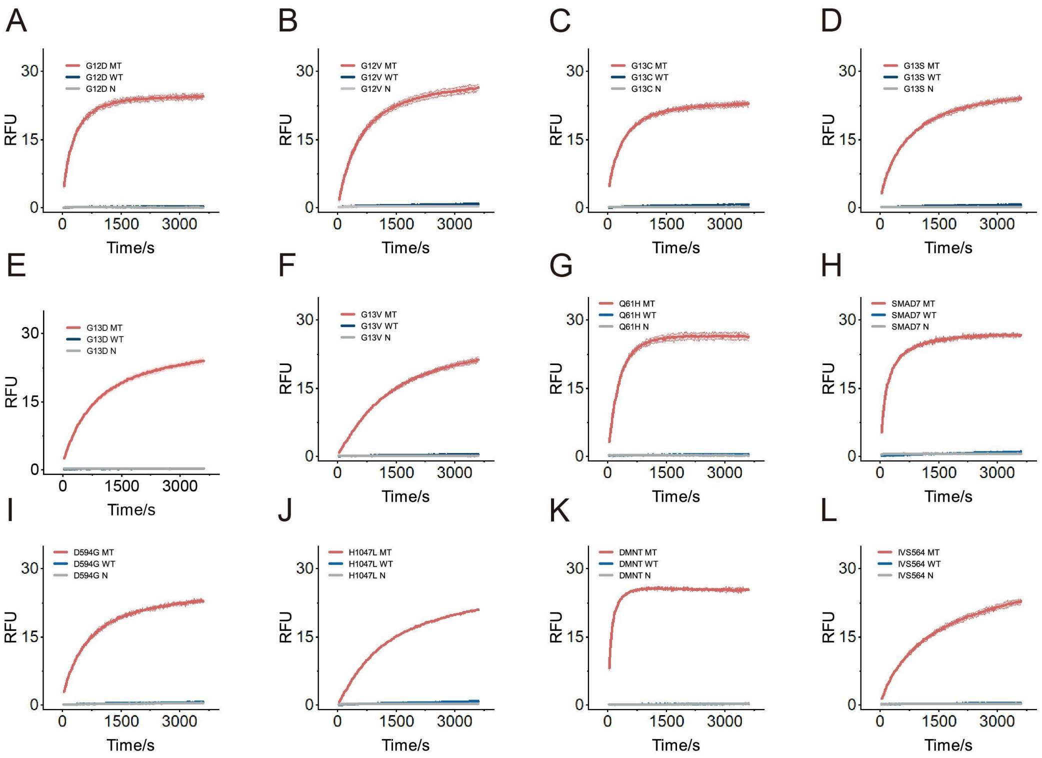


Figure S43. The part of the real-time fluorescence curve corresponding to Figure 4G.

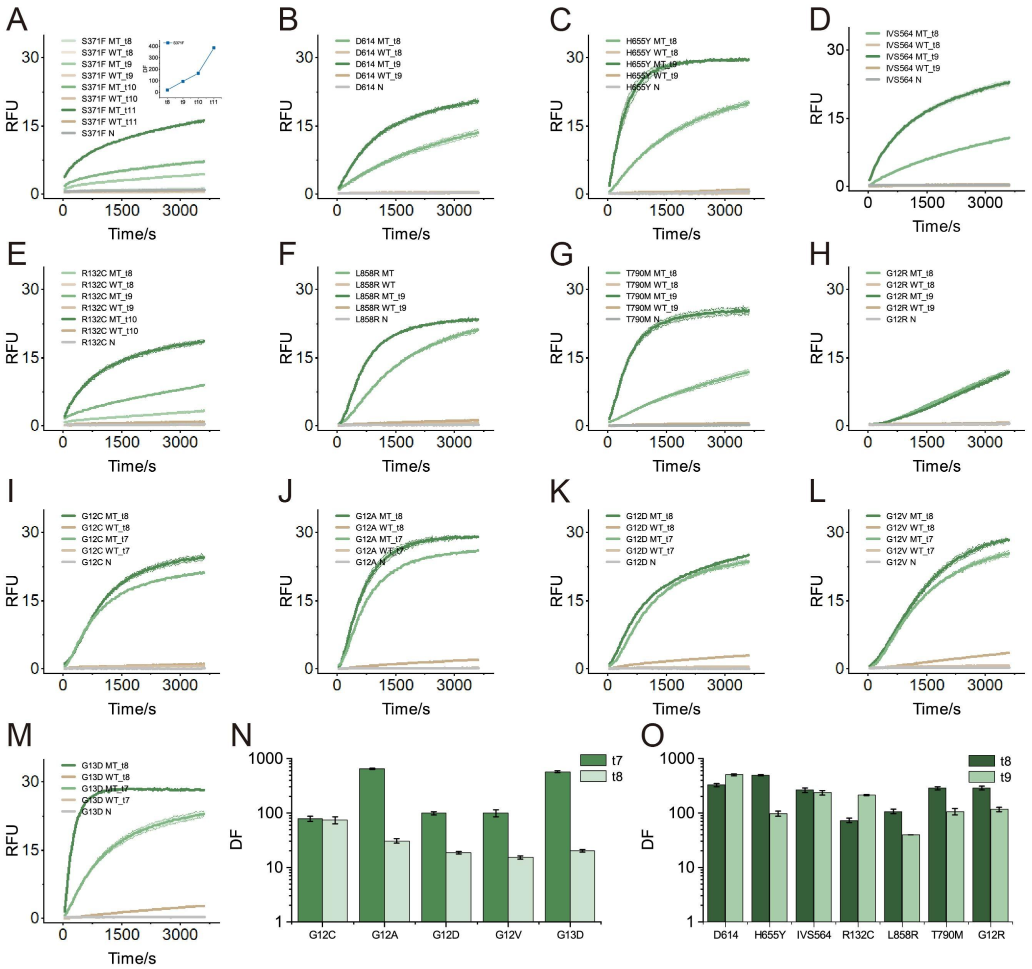

Figure S44. (A-M) Real-time fluorescence curves demonstrating the effect of changing toehold on some of the targets. (N-O) DF changes before and after changing the target toehold.

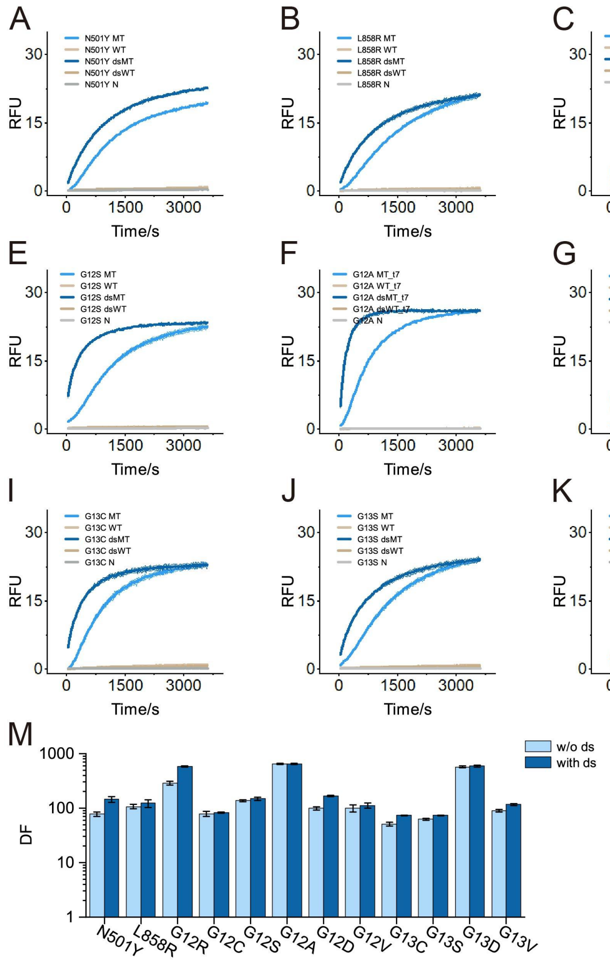


Figure S45. (A-L) Real-time fluorescence curves demonstrating the effect of pre-assembled targets and helper strands on some of the targets. (M) DF changes before and after preassembly. The ds is preassembled. Not stated is not pre-assembled. Default is toehold of 8 nt unless otherwise stated.

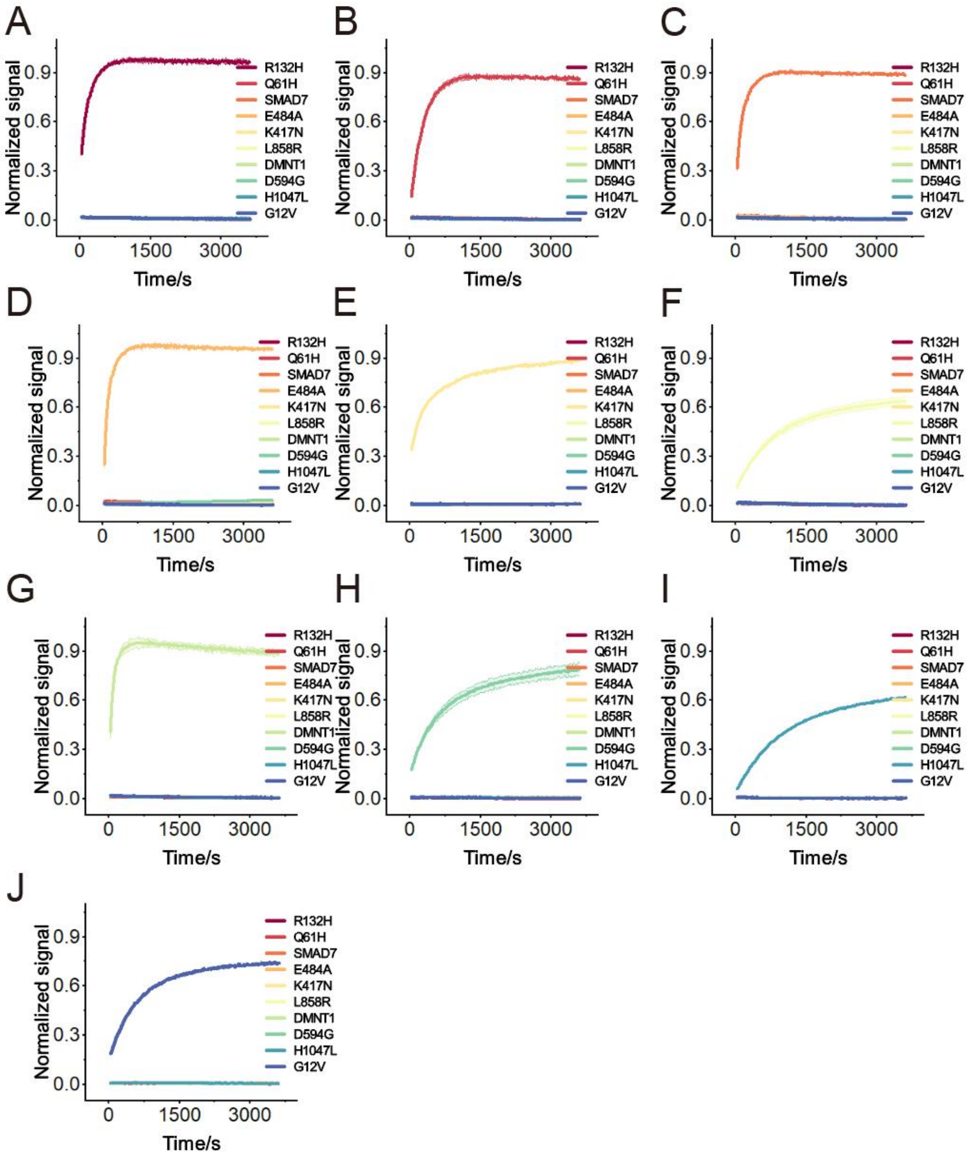


Figure S46. Fluorescence real-time curves for orthogonality analysis when the targets were (A) R132H, (B) Q61H, (C) SMAD7, (D) E484A, (E) K417N, (F) L858R, (G) DMNT1, (H) D594G, (I) H1047L, (J) G12V.

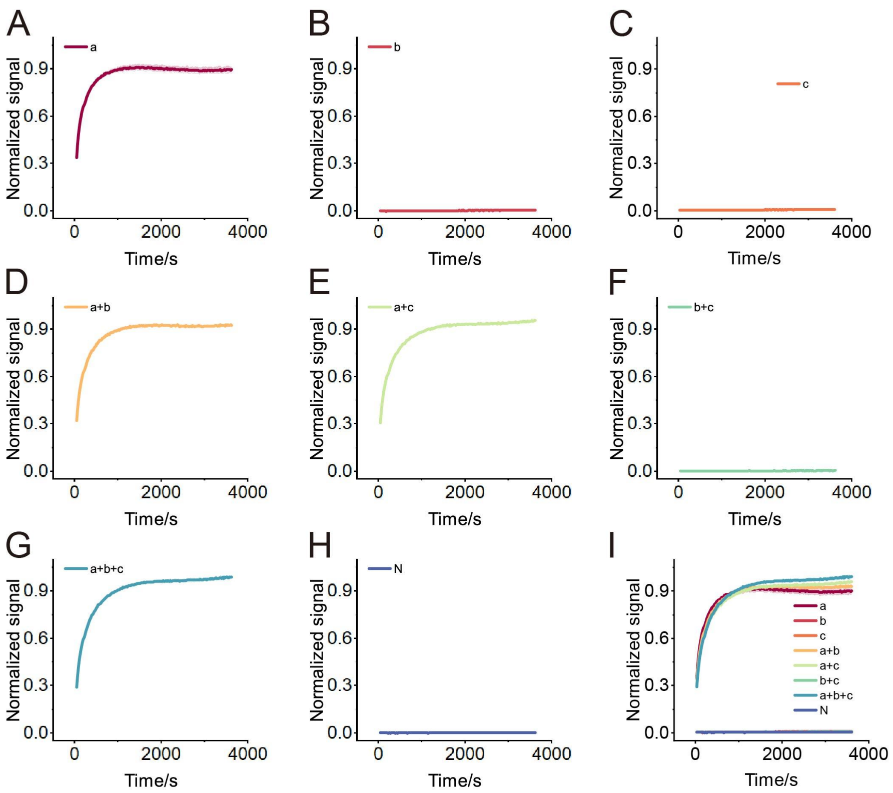


Figure S47. Detection of different plasmid combinations in the red channel. The a represents IDH1 R132H, b represents KRAS G12V, and c represents SARS-CoV-2 E484A.

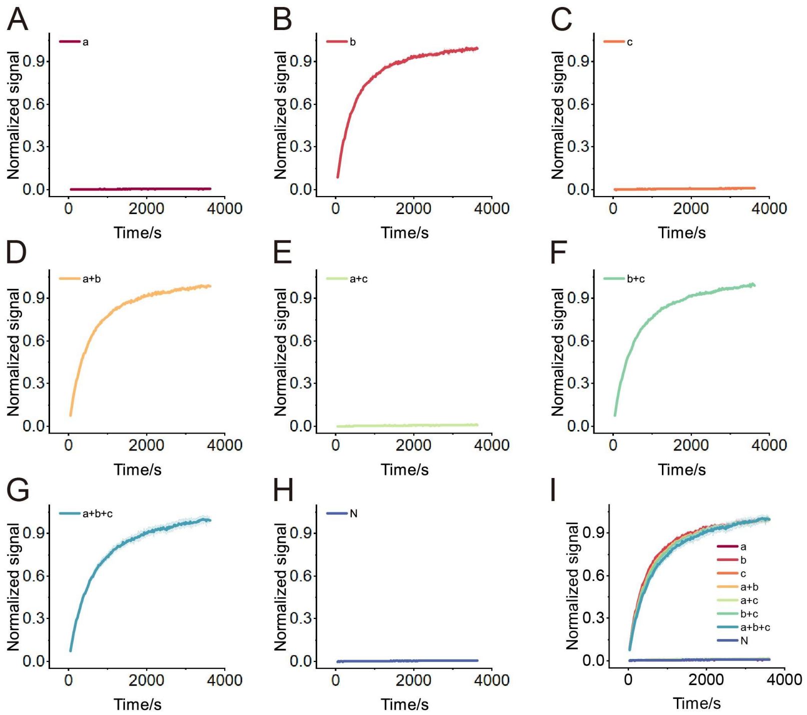


Figure S48. Detection of different plasmid combinations in the orange channel. The a represents IDH1 R132H, b represents KRAS G12V, and c represents SARS-CoV-2 E484A.

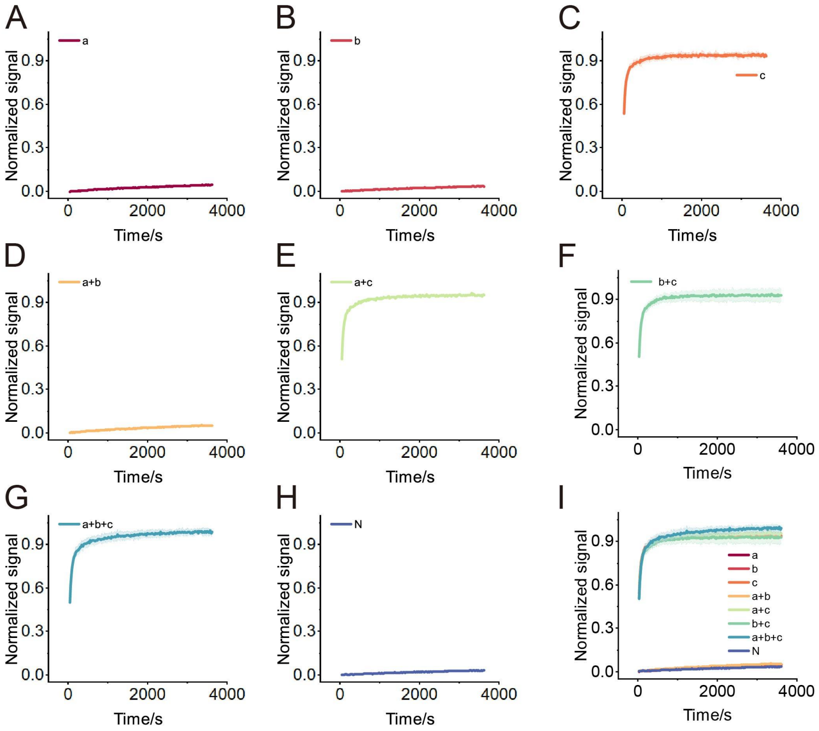


Figure S49. Detection of different plasmid combinations in the yellow channel. The a represents IDH1 R132H, b represents KRAS G12V, and c represents SARS-CoV-2 E484A.

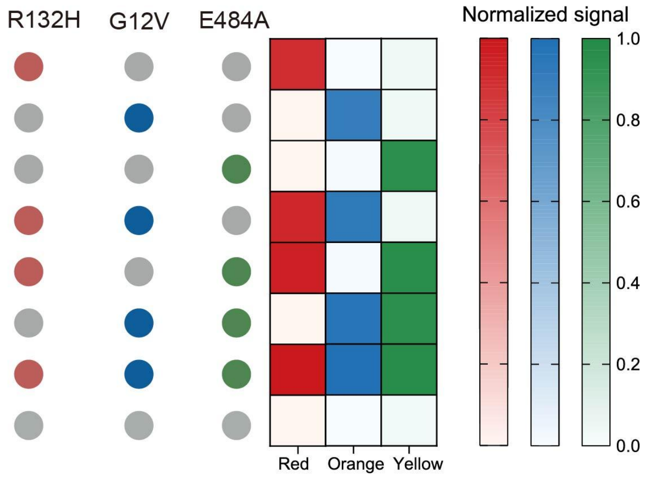


Figure S50. Evaluation of sample combinations and their compatibility with the mcSDR probe cocktail for analysis of IDH1, KRAS, and SARS-CoV-2.

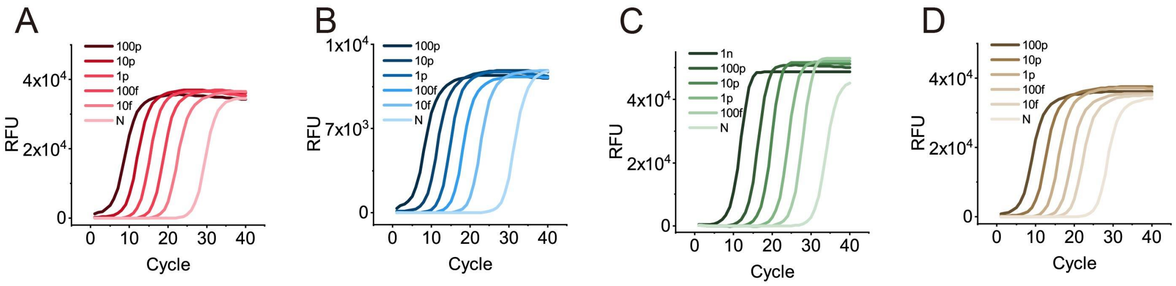


Figure S51. Real-time fluorescence curves of different concentrations of IDH1 detected in (A) SG-Taqman PCR Mix (B) Vazyme PCR Mix (C) Diomand PCR Mix (D) TG PCR Mix.

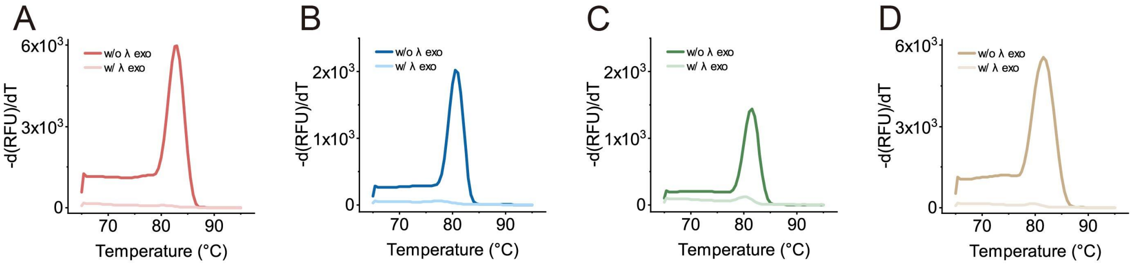

Figure S52. Melting curves of IDH1 PCR amplification products with and without λ exo in (A) SG-Taqman PCR Mix (B) Vazyme PCR Mix (C) Diomand PCR Mix (D) TG PCR Mix.

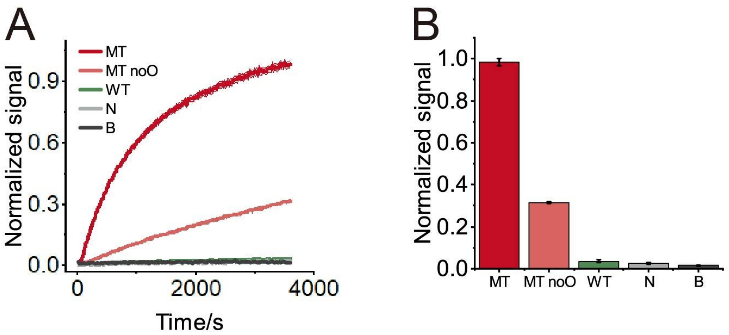


Figure 53. (A) fluorescence curve feasibility and (B) Comparison of normalized signal of IDH1 PCR products detected by mcSDR.

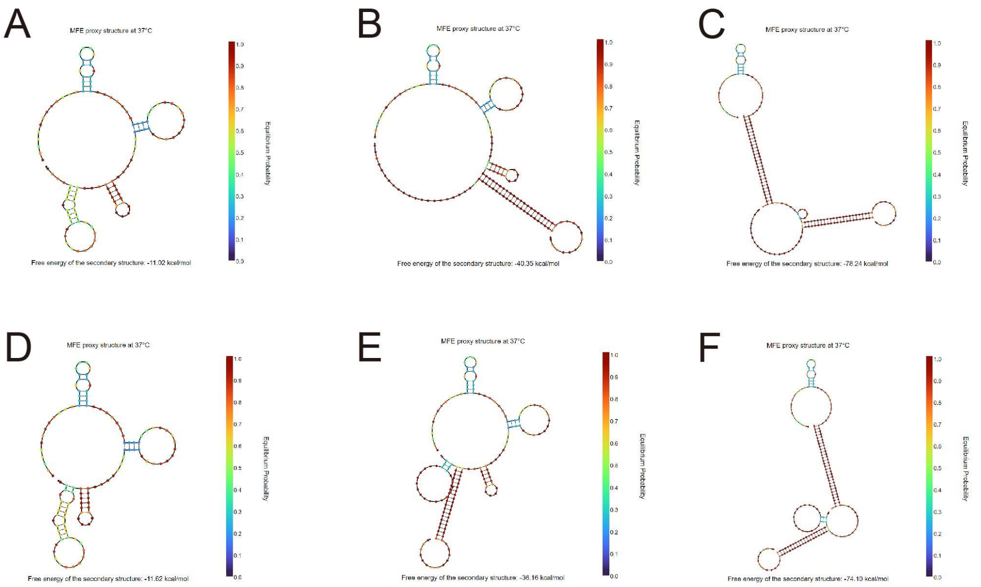


Figure 54. Nupack simulation of IDH1 wild-type targets with secondary structure of (A) the single- stranded product, (B) the complex of the single-stranded product and helper strand, and (C) the complex of the single-stranded product, helper strand, and opener. Nupack simulation of IDH1 mutant targets with secondary structure of (D) the single-stranded product, (E) the complex of the single-stranded product and helper strand, and (F) the complex of the single-stranded product, helper strand, and opener.

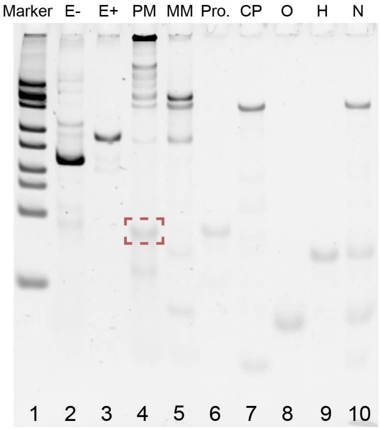


Figure S55. PAGE analysis of IDH1 target-triggered mcSDR. lane 1 is the 25 bp marker. lane 2 is the PCR product. Lane 3 is the enzyme digestion product. Lane 4 is the product of the PM target after amplification and digestion and reaction with the probe. Lane 5 is the product of the MM target after amplification and digestion and reaction with the probe. Lane 6 is the single-stranded product. Swim lane 7 is the probe. Lane 8 is the opener strand. Lane 9 is the helper strand. Lane 10 is the product of the reaction without the target.

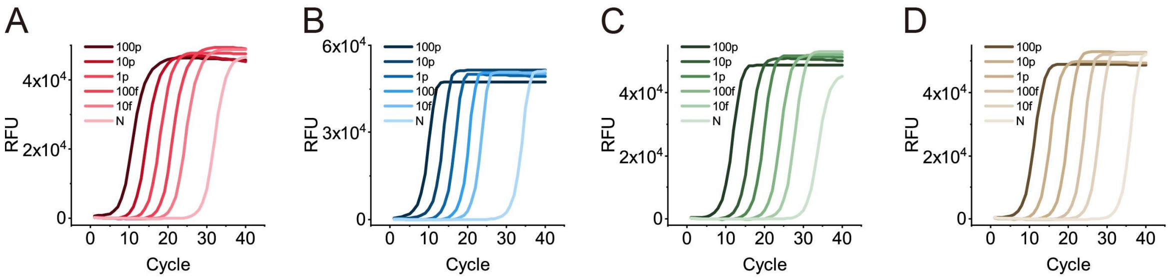


Figure S56. Real-time fluorescence curves of different concentrations of KRAS detected in (A) SG-Taqman PCR Mix (B) Vazyme PCR Mix (C) Diomand PCR Mix (D) TG PCR Mix.

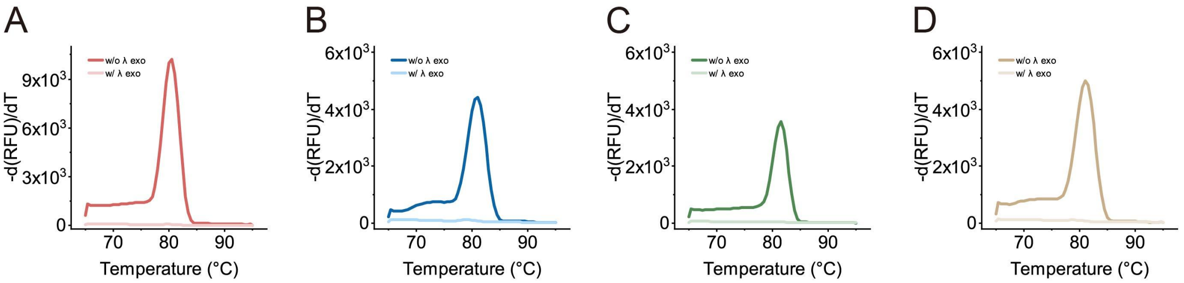


Figure S57. Melting curves of KRAS PCR amplification products with and without λ exo in (A) SG-Taqman PCR Mix (B) Vazyme PCR Mix (C) Diomand PCR Mix (D) TG PCR Mix.

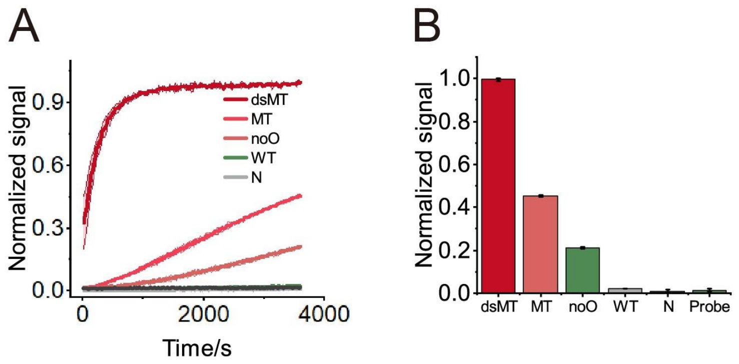


Figure 58. (A) fluorescence curve feasibility and (B) Comparison of normalized signal of KRAS PCR products detected by mcSDR.

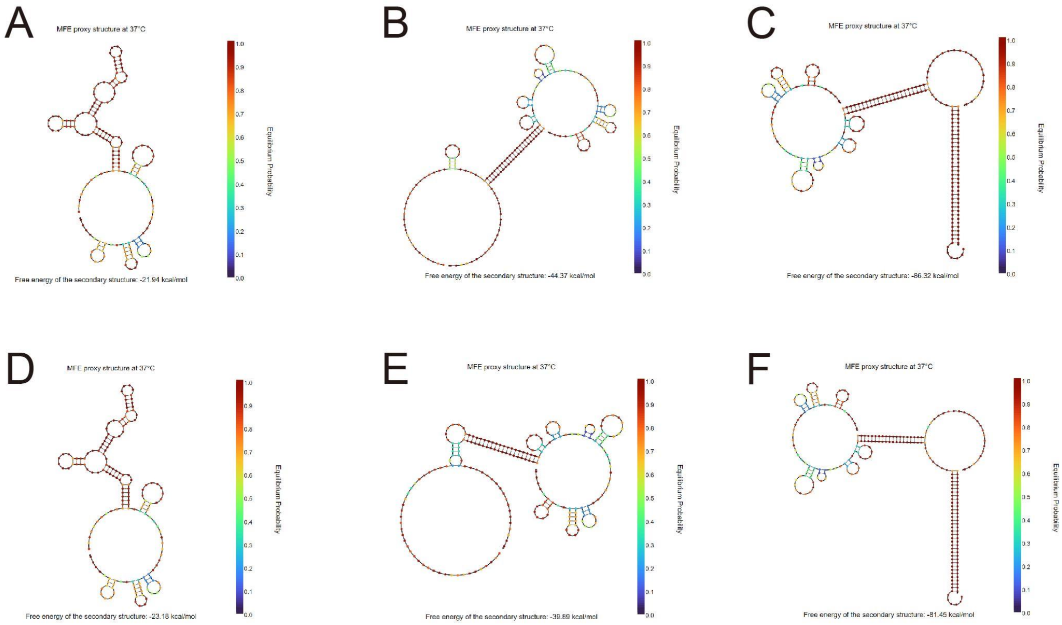


Figure 59. Nupack simulation of KRAS wild-type targets with secondary structure of (A) the single- stranded product, (B) the complex of the single-stranded product and helper strand, and (C) the complex of the single-stranded product, helper strand, and opener. Nupack simulation of KRAS mutant targets with secondary structure of (D) the single-stranded product, (E) the complex of the single-stranded product and helper strand, and (F) the complex of the single-stranded product, helper strand, and opener.

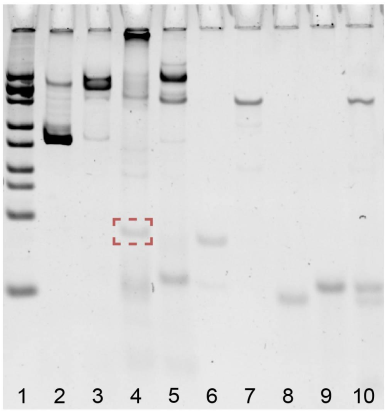


Figure S60. PAGE analysis of KRAS target-triggered mcSDR. lane 1 is the 25 bp marker. lane 2 is the PCR product. Lane 3 is the enzyme digestion product. Lane 4 is the product of the PM target after amplification and digestion and reaction with the probe. Lane 5 is the product of the MM target after amplification and digestion and reaction with the probe. Lane 6 is the single-stranded product. Swim lane 7 is the probe. Lane 8 is the opener strand. Lane 9 is the helper strand. Lane 10 is the product of the reaction without the target.

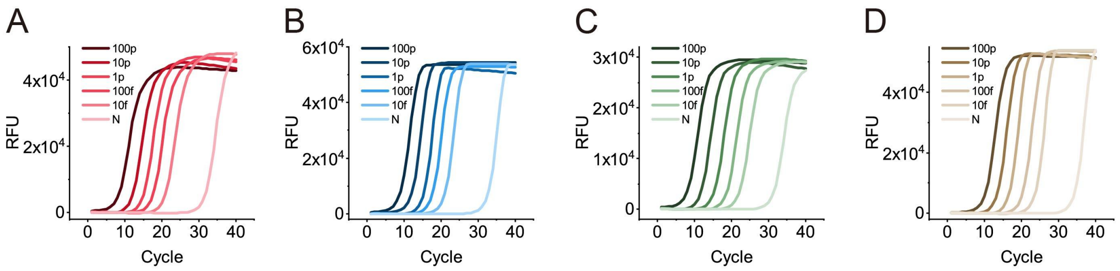


Figure S61. Real-time fluorescence curves of different concentrations of SARS-CoV-2 detected in (A) SG-Taqman PCR Mix (B) Vazyme PCR Mix (C) Diomand PCR Mix (D) TG PCR Mix.

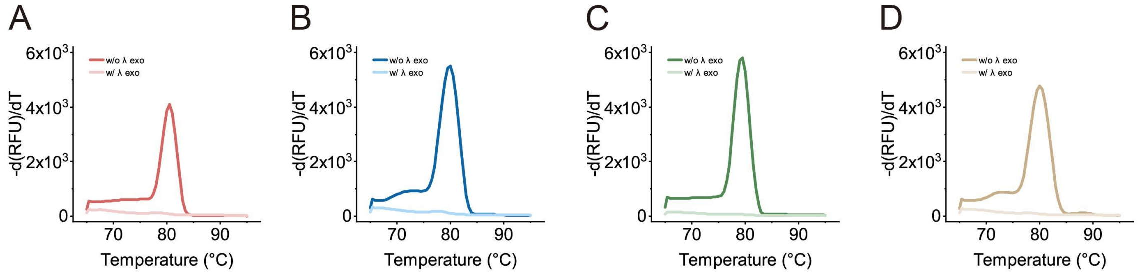


Figure S62. Melting curves of SARS-CoV-2 PCR amplification products with and without λ exo in (A) SG-Taqman PCR Mix (B) Vazyme PCR Mix (C) Diomand PCR Mix (D) TG PCR Mix.

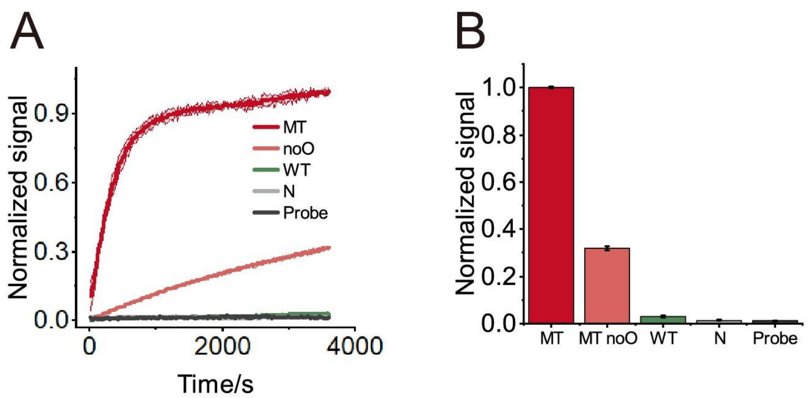


Figure 63. (A) fluorescence curve feasibility and (B) Comparison of normalized signal of SARS-CoV-2 PCR products detected by mcSDR.

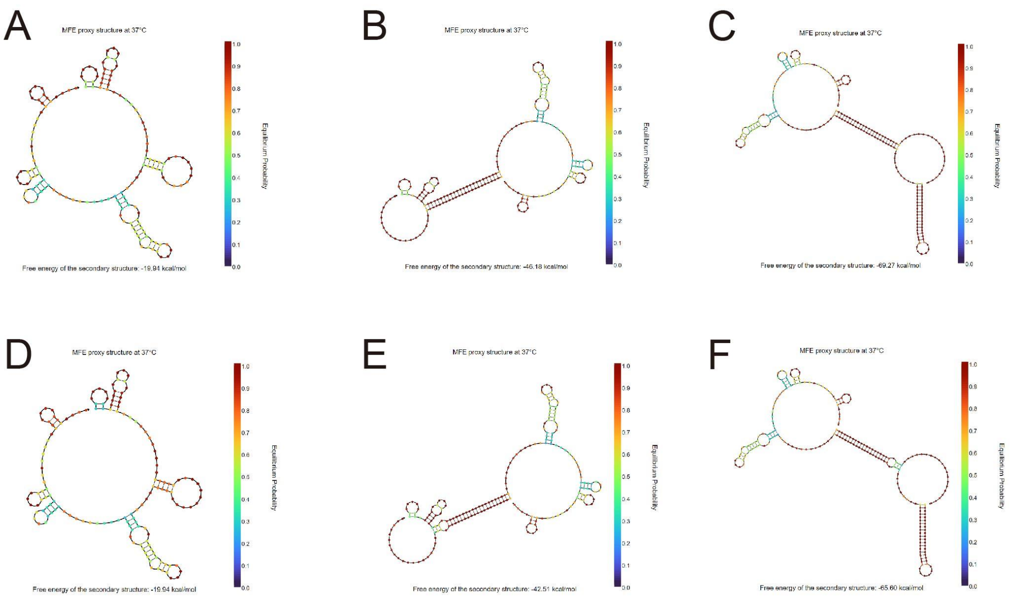


Figure 64. Nupack simulation of SARS-CoV-2 wild-type targets with secondary structure of (A) the single- stranded product, (B) the complex of the single-stranded product and helper strand, and (C) the complex of the single-stranded product, helper strand, and opener. Nupack simulation of SARS-CoV-2 mutant targets with secondary structure of (D) the single-stranded product, (E) the complex of the single-stranded product and helper strand, and (F) the complex of the single-stranded product, helper strand, and opener.

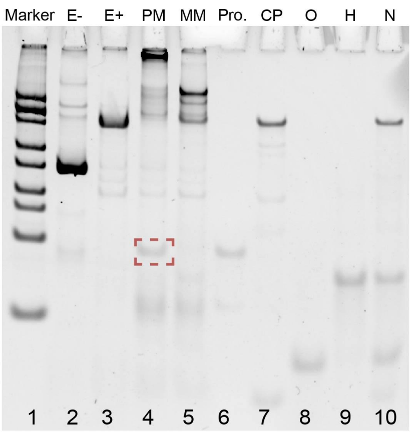


Figure S65. PAGE analysis of SARS-CoV-2 target-triggered mcSDR. lane 1 is the 25 bp marker. lane 2 is the PCR product. Lane 3 is the enzyme digestion product. Lane 4 is the product of the PM target after amplification and digestion and reaction with the probe. Lane 5 is the product of the MM target after amplification and digestion and reaction with the probe. Lane 6 is the single-stranded product. Swim lane 7 is the probe. Lane 8 is the opener strand. Lane 9 is the helper strand. Lane 10 is the product of the reaction without the target.

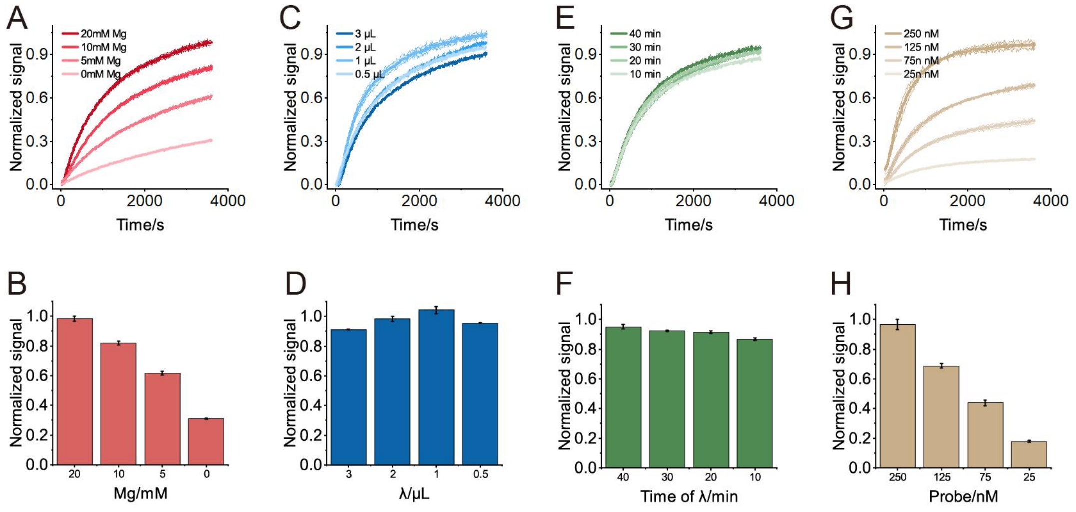


Figure S66. Optimization of PCR-mcSDR for the detection of IDH1 SNV including fluorescence curves and corresponding bar graphs (A-B) $Mg^{2+}$ concentration (optimized to 20 mM), (C-D) enzyme dosage (optimized to 1 μl), (E-F) enzyme digestion time (optimized to 30 min), and (G-H) probe concentration (optimized to 250 nM).

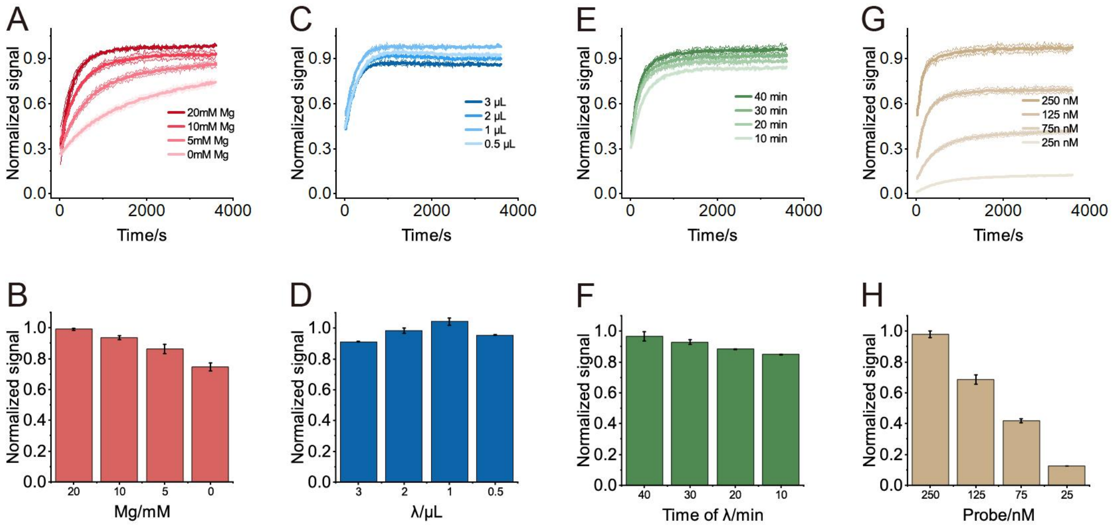


Figure S67. Optimization of PCR-mcSDR for the detection of KRAS SNV including fluorescence curves and corresponding bar graphs (A-B) $Mg^{2+}$ concentration (optimized to 20 mM), (C-D) enzyme dosage (optimized to 1 μl), (E-F) enzyme digestion time (optimized to 30 min), and (G-H) probe concentration (optimized to 250 nM).

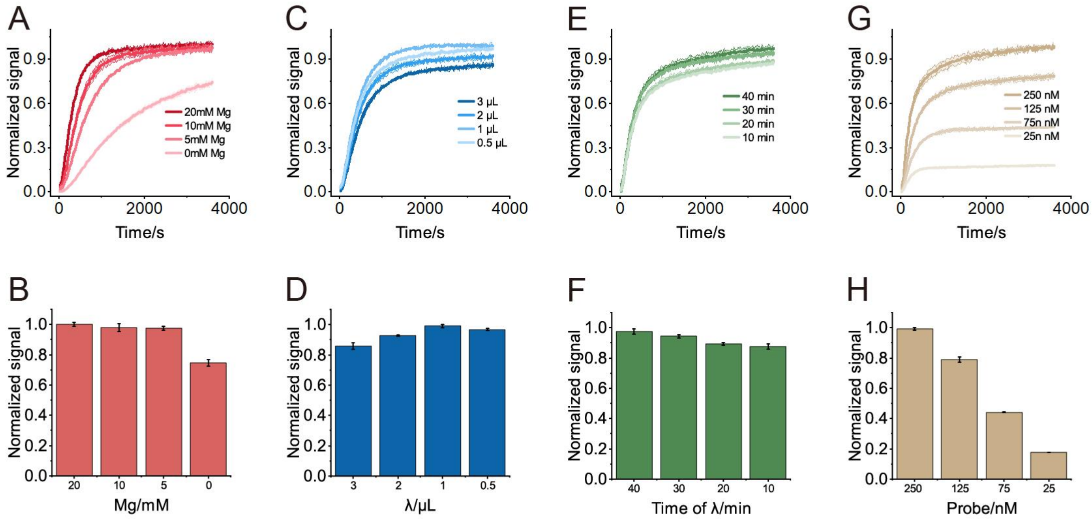


Figure S68. Optimization of PCR-mcSDR for the detection of SARS-CoV-2 SNV including fluorescence curves and corresponding bar graphs (A-B) $Mg^{2+}$ concentration (optimized to 20 mM), (C-D) enzyme dosage (optimized to 1 μl), (E-F) enzyme digestion time (optimized to 30 min), and (G-H) probe concentration (optimized to 250 nM).

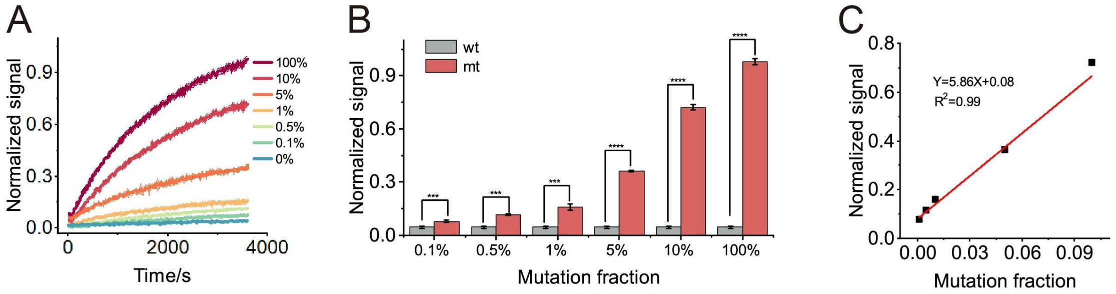


Figure S69. (A) Fluorescence real-time curve of PCR-mcSDR detection of different abundances of IDH1 SNV. (B) Detection limits for distinguishing different IDH1 SNV abundances ranged from 0.1 to 100%. (C) Normalized signals of IDH1 SNV were linearly related to mutation abundance from 0.1% to 10%.

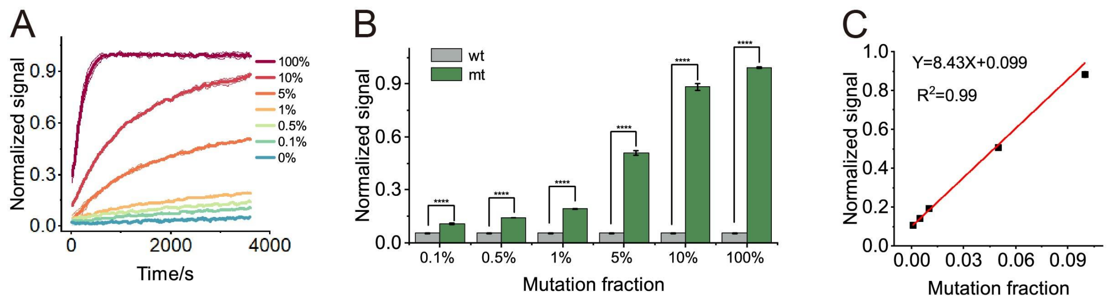


Figure S70. (A) Fluorescence real-time curves for PCR-mcSDR detection of different abundances of KRAS SNV. (B) Detection limits for distinguishing different KRAS SNV abundances ranged from 0.1 to 100%. (C) Linearity of the normalized signal of KRAS SNV with mutation abundance from 0.1% to 10%.

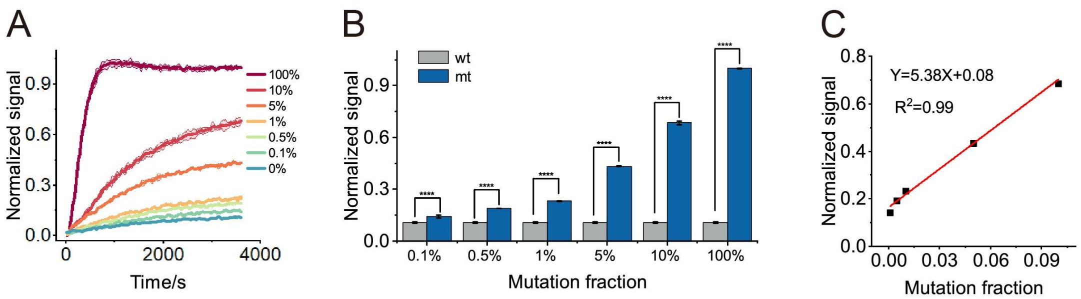


Figure S71. (A) Fluorescence real-time curve of PCR-mcSDR for the detection of different abundances of SARS-CoV-2 SNV. (B) Detection limits for distinguishing different SARS-CoV-2 SNV abundances ranged from 0.1 to 100%. (C) The linear relationship between the normalized signal of SARS-CoV-2 SNV and the mutation abundance from 0.1% to 10%.

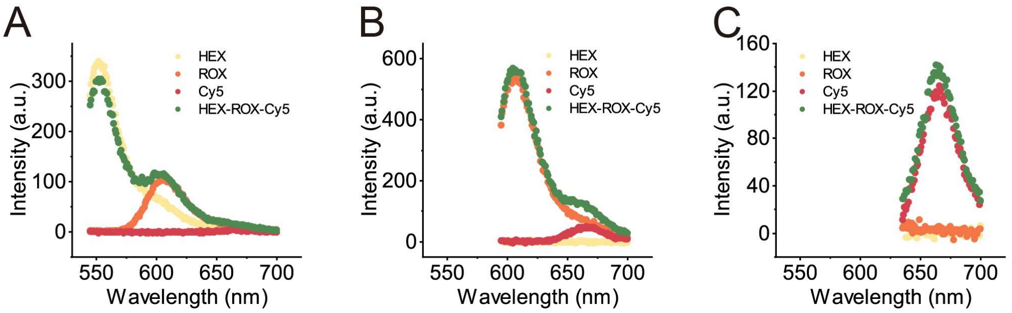


Figure S72. Emission spectra of three fluoropbores (HEX, ROX, and Cy5) at excitation wavelengths of 530 nm (A), 585 nm (B), and 625 nm (C), respectively. The emission spectra were collected with a scanning step of 5 nm.

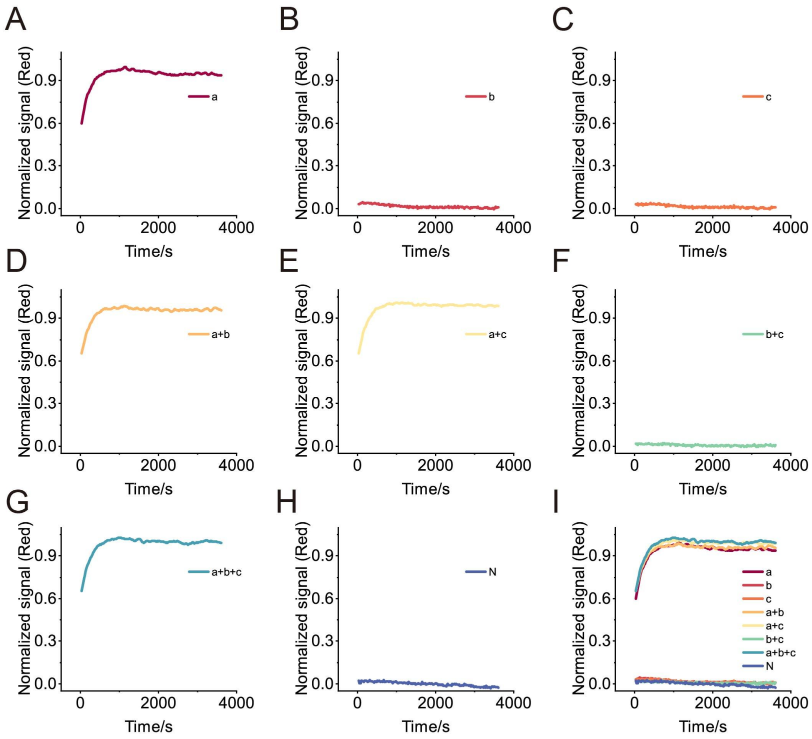


Figure S73. Detection of different plasmid combinations in the red channel. (A) IDH1 R132H (B) KRAS G12V (C) SARS-CoV-2 E484A (D) IDH1 R132H and KRAS G12V (E) IDH1 R132H and SARS-CoV-2 E484A (F) (G) IDH1 R132H and SARS-CoV-2 E484A (H) (I) IDH1 R132H, KRAS G12V and SARS-CoV-2 E484A.

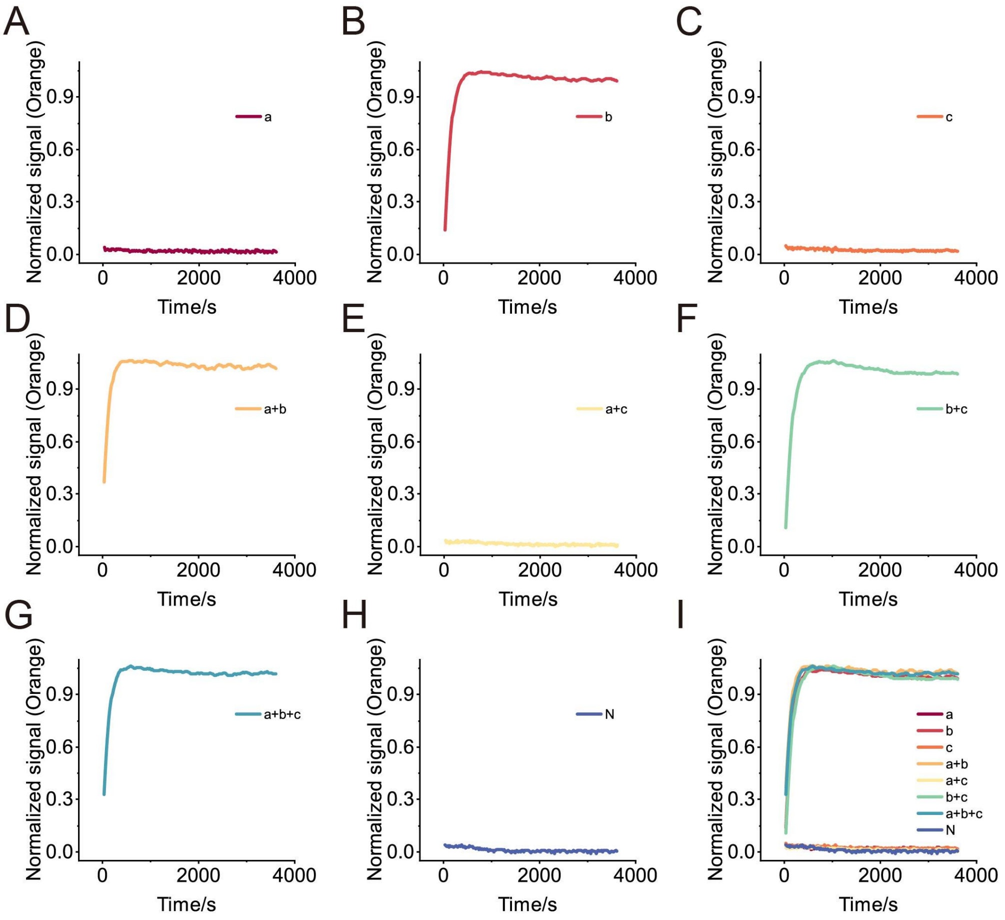


Figure S74. Detection of different plasmid combinations in the orange channel. (A) IDH1 R132H (B) KRAS G12V (C) SARS-CoV-2 E484A (D) IDH1 R132H and KRAS G12V (E) IDH1 R132H and SARS-CoV-2 E484A (F) (G) IDH1 R132H and SARS-CoV-2 E484A (H) (I) IDH1 R132H, KRAS G12V and SARS-CoV-2 E484A.

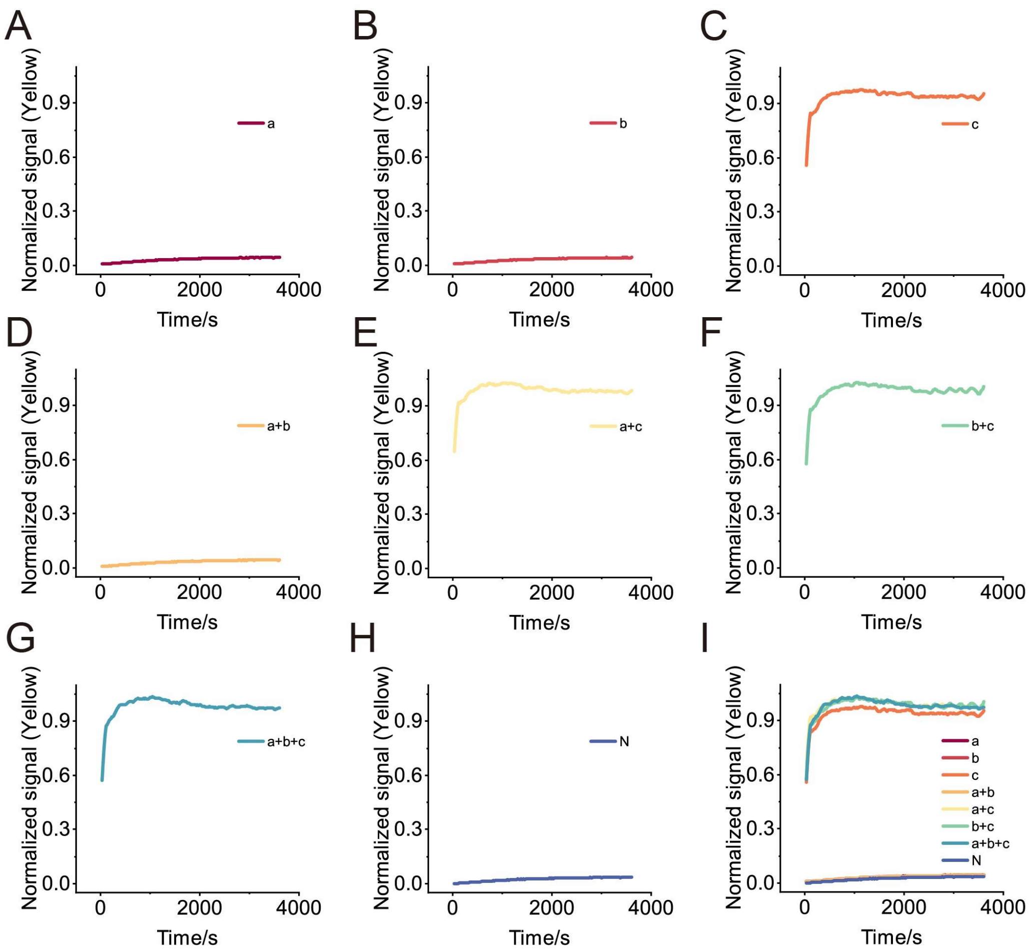


Figure S75. Detection of different plasmid combinations in the yellow channel. (A) IDH1 R132H (B) KRAS G12V (C) SARS-CoV-2 E484A (D) IDH1 R132H and KRAS G12V (E) IDH1 R132H and SARS-CoV-2 E484A (F) (G) IDH1 R132H and SARS-CoV-2 E484A (H) Negative samples (I) IDH1 R132H, KRAS G12V and SARS-CoV-2 E484A.

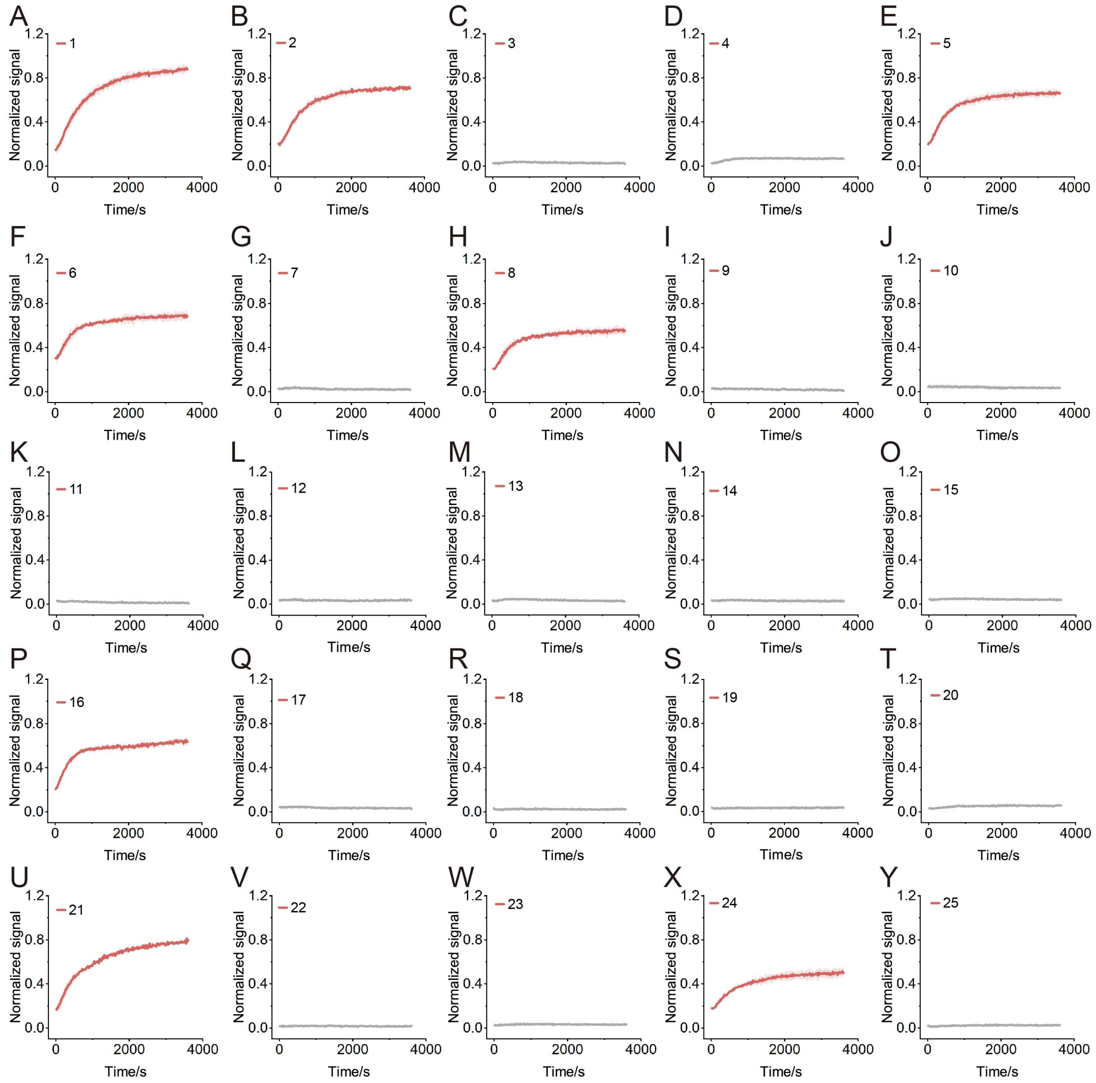


Figure S76. mcSDR fluorescence real-time curve results of brain glioma samples numbered 1-25.

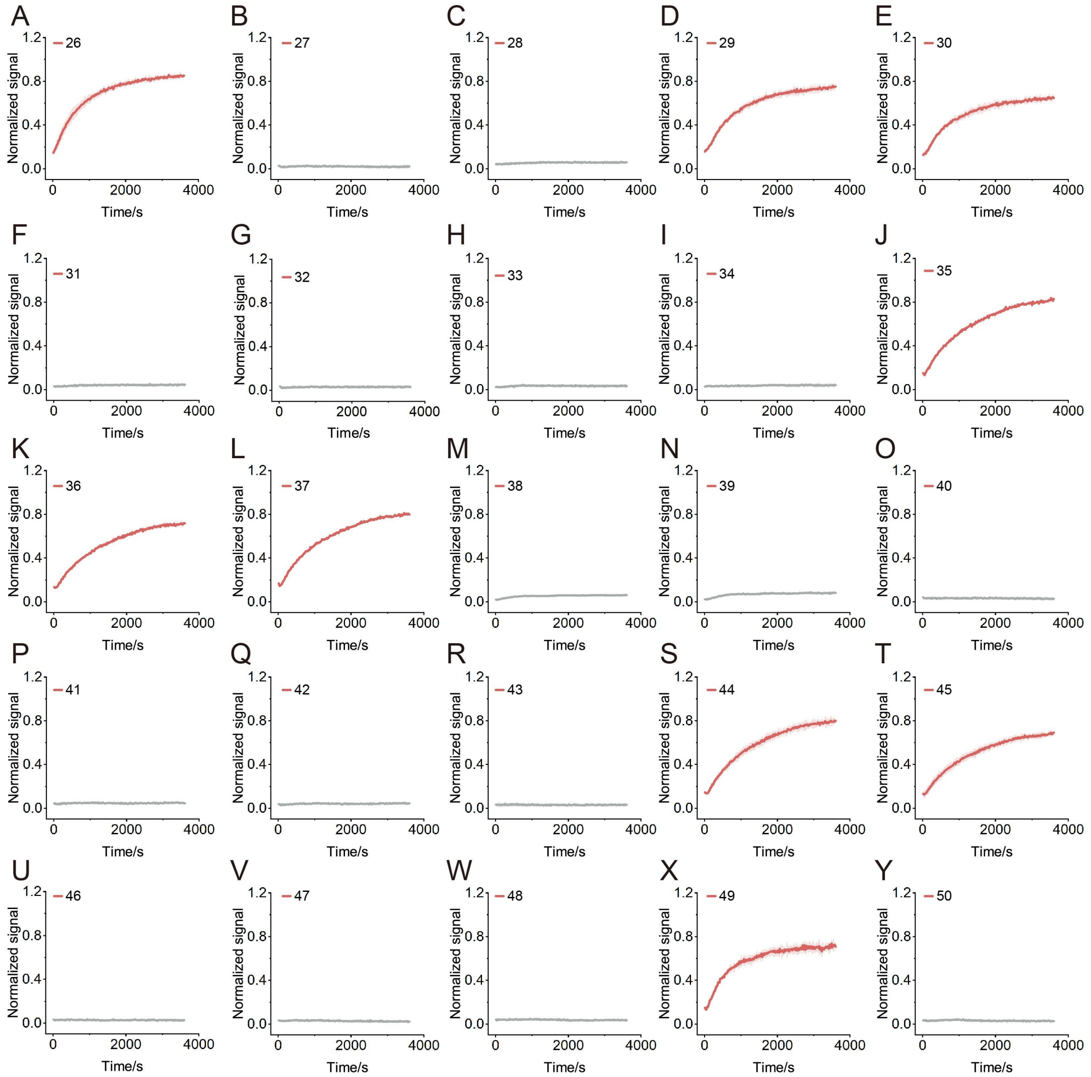


Figure S77. mcSDR fluorescence real-time curve results of brain glioma samples numbered 26-50.

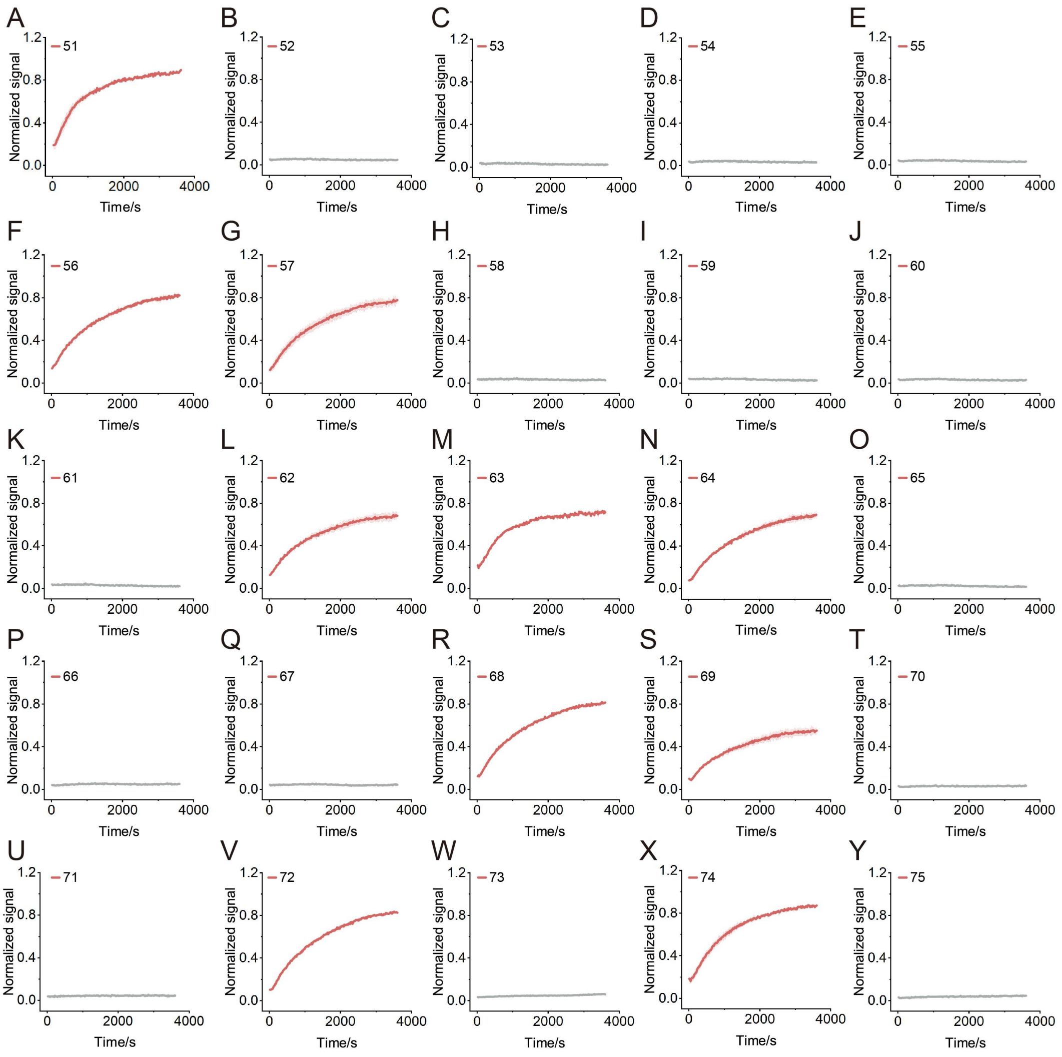


Figure S78. mcSDR fluorescence real-time curve results of brain glioma samples numbered 51-75.

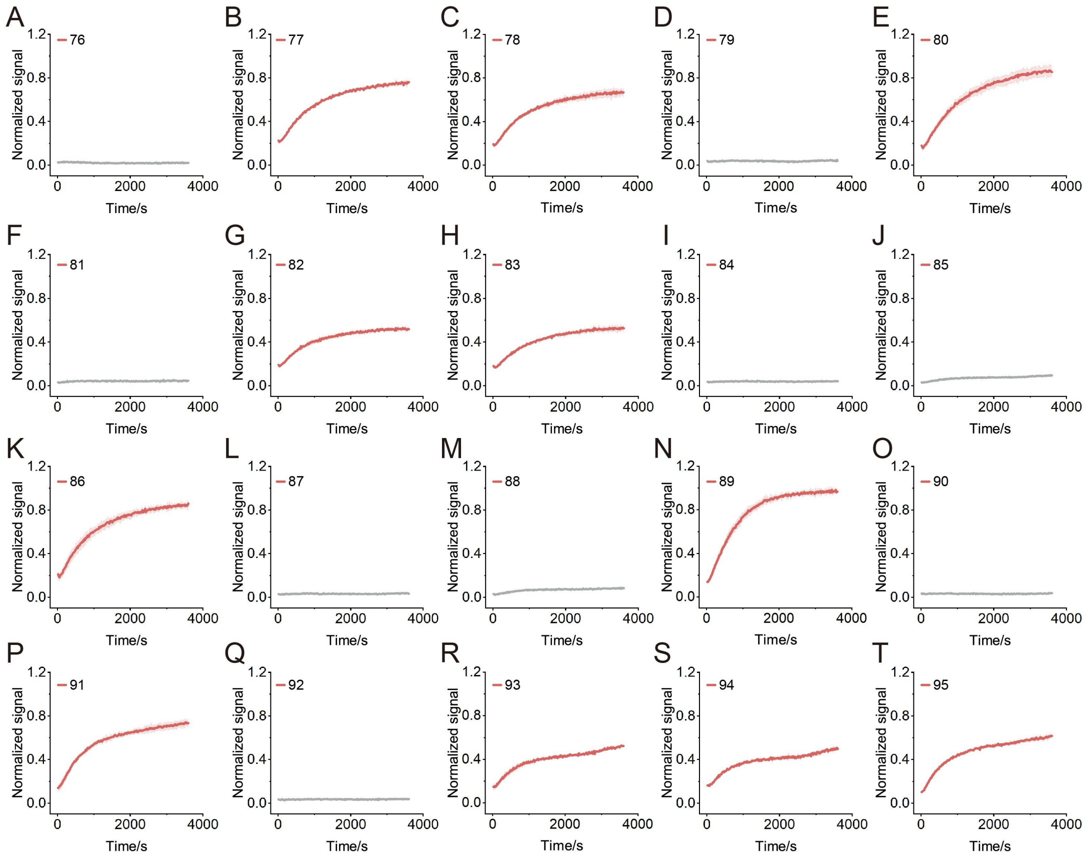


Figure S79. mcSDR fluorescence real-time curve results of brain glioma samples numbered 76-95.

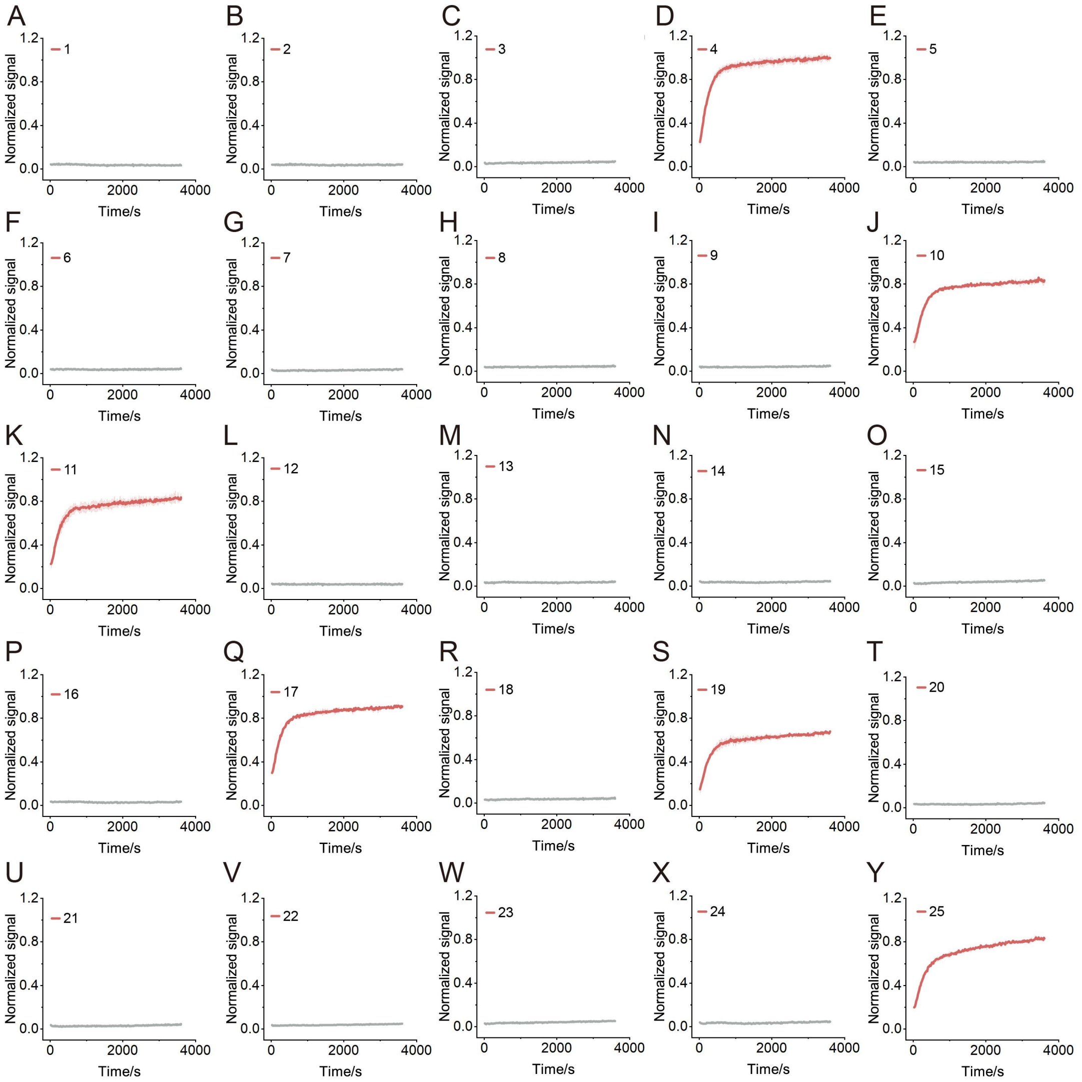


Figure S80. mcSDR fluorescence real-time curve results of colorectal cancer samples numbered 1-25.

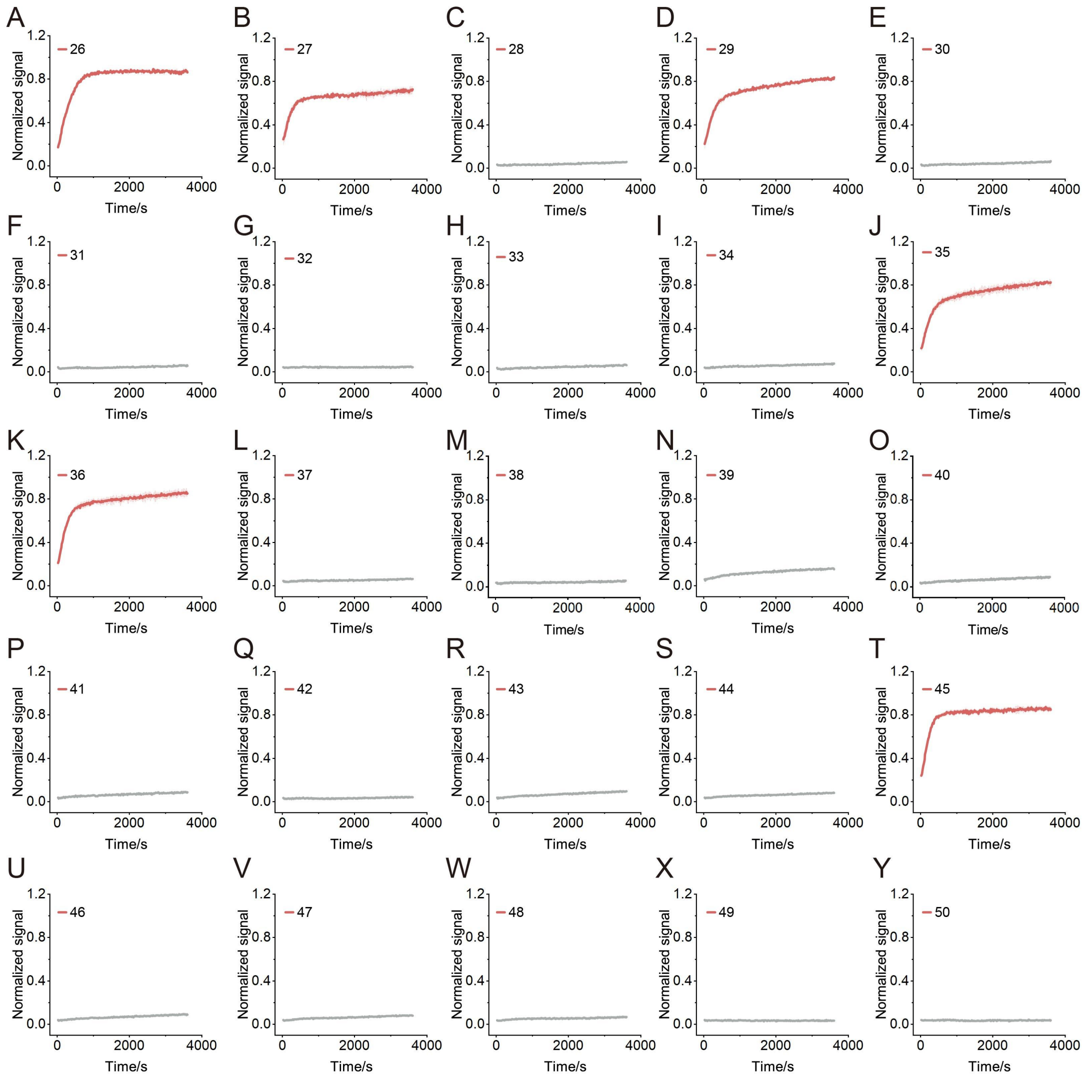


Figure S81. mcSDR fluorescence real-time curve results of colorectal cancer samples numbered 26-50.

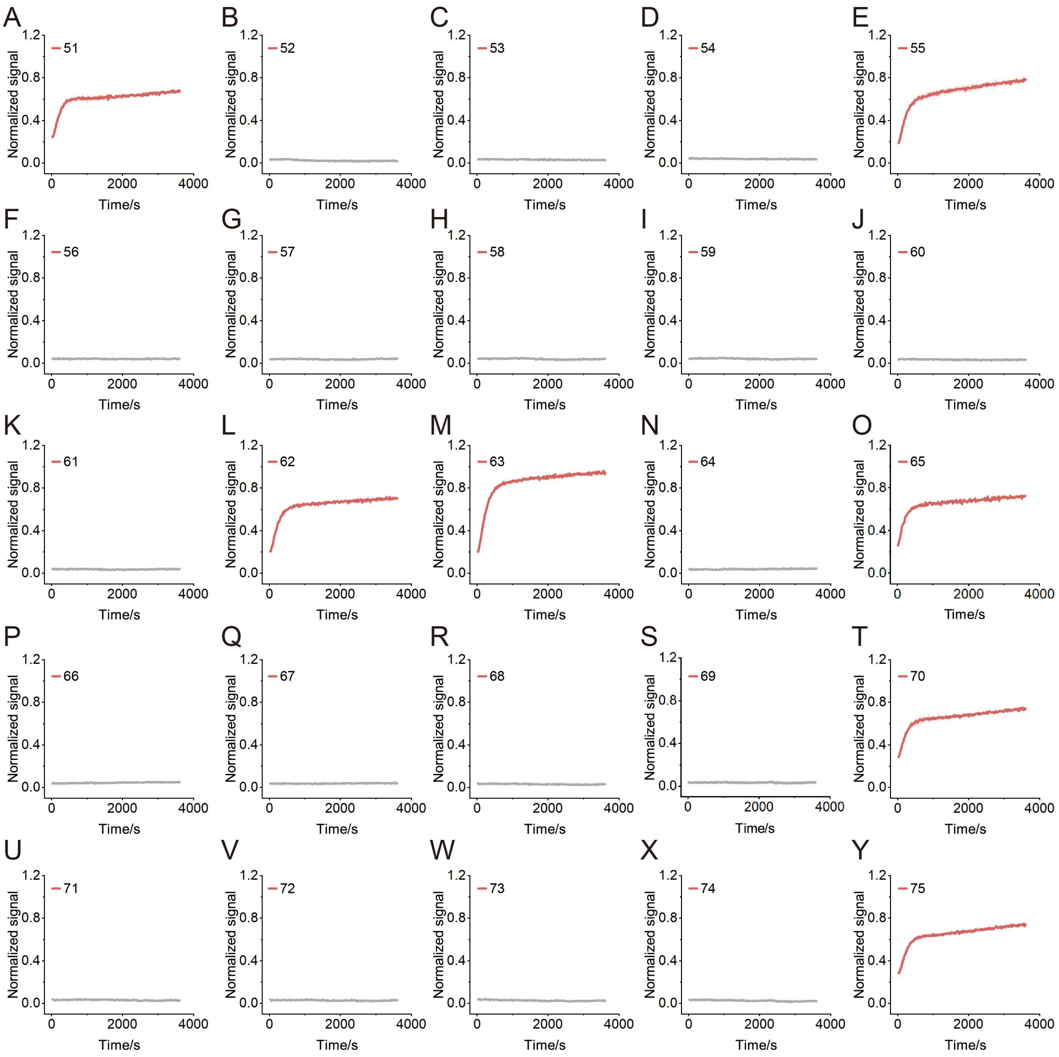


Figure S82. mcSDR fluorescence real-time curve results of colorectal cancer samples numbered 51-75.

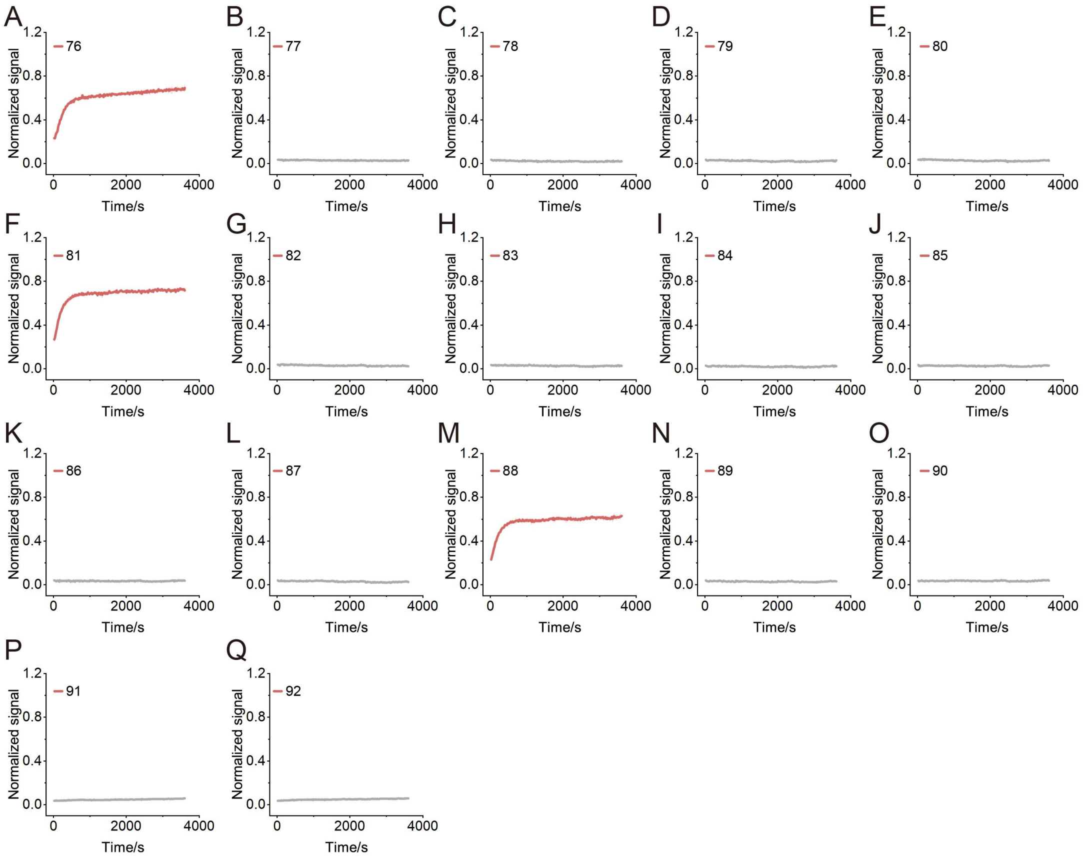


Figure S83. mcSDR fluorescence real-time curve results of colorectal cancer samples numbered 76-93.

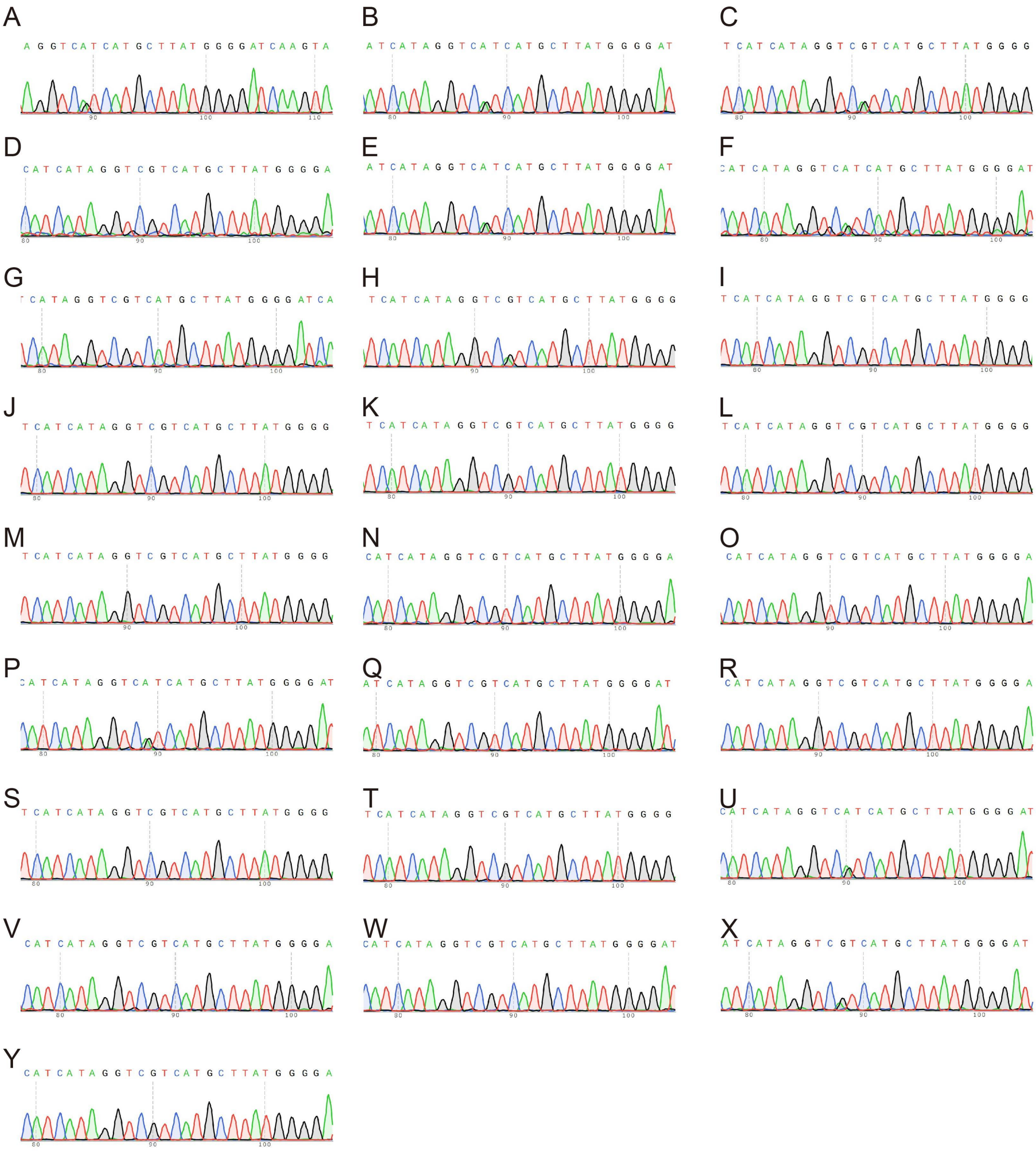

Figure S84. Sanger results of brain glioma samples numbered 1-25.

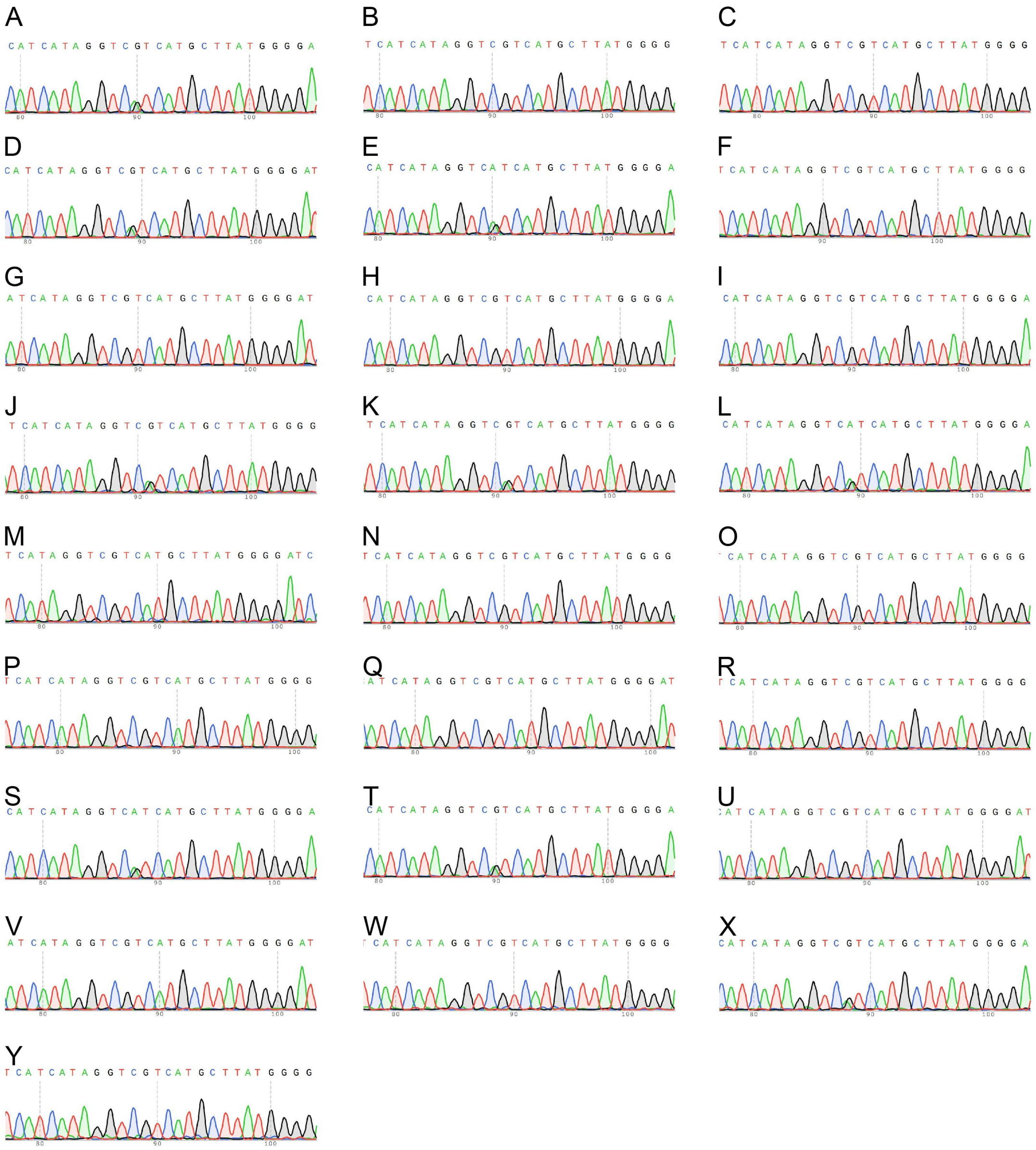


Figure S85. Sanger results for brain glioma samples numbered 26-50.

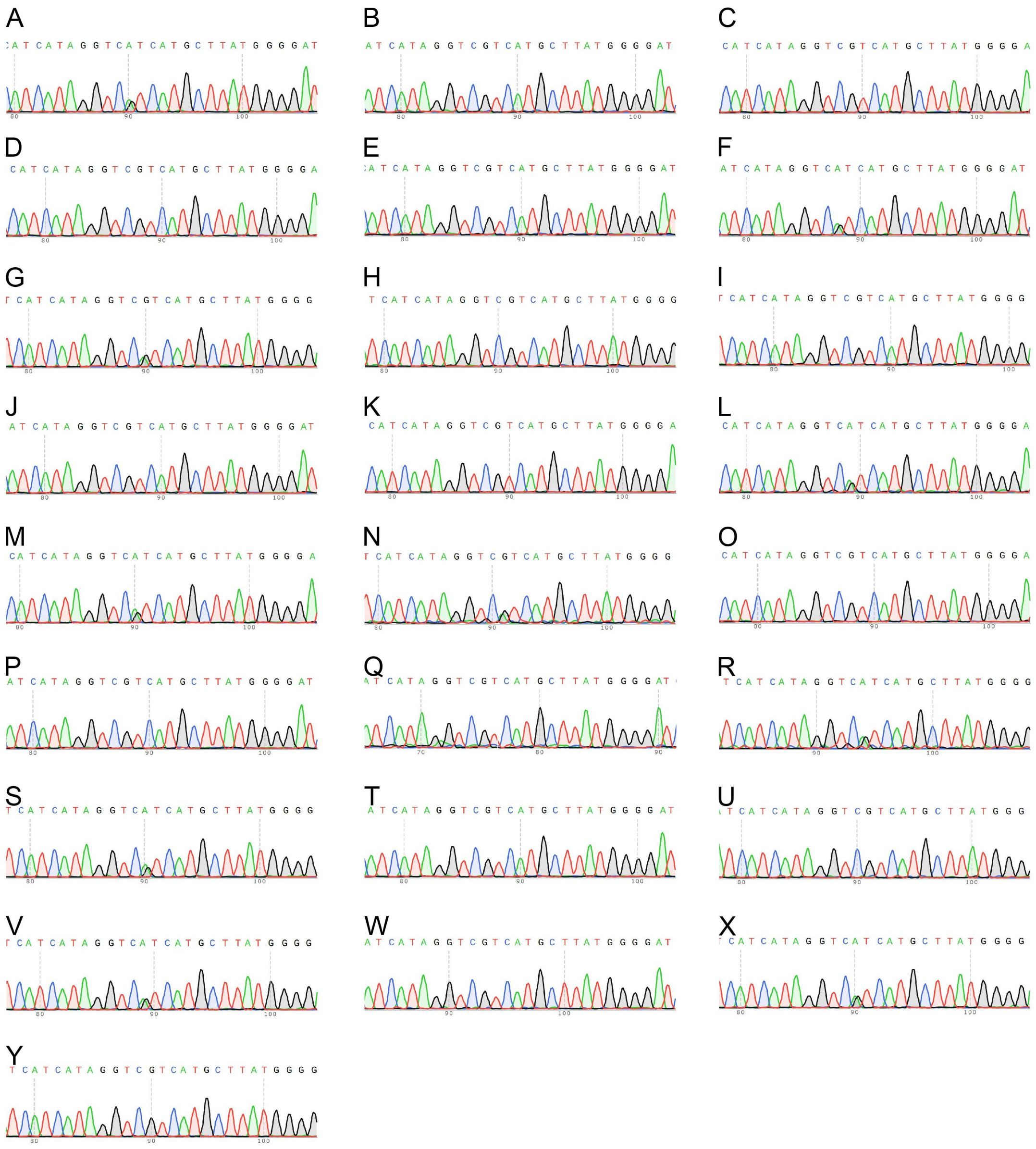

Figure S86. Sanger results for brain glioma samples numbered 51-75.

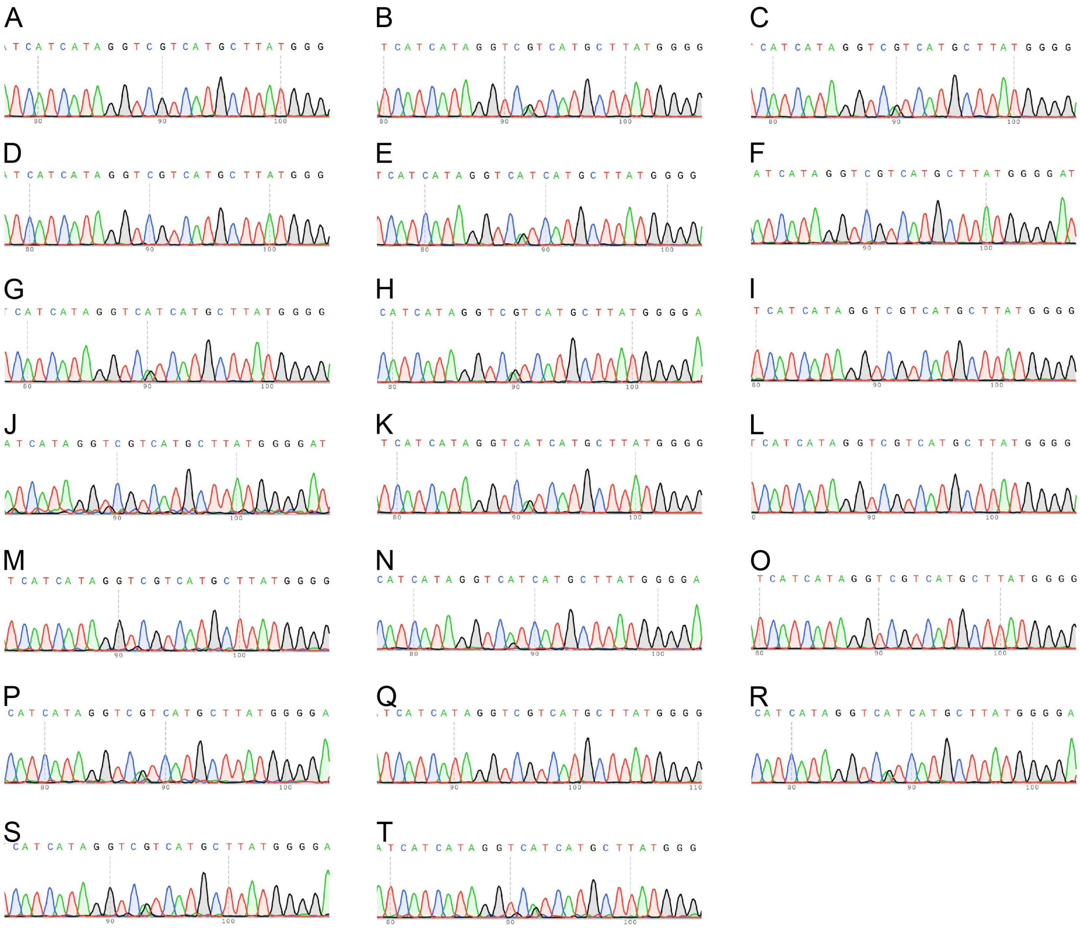


Figure S87. Sanger results for colorectal brain glioma numbered 76-95.

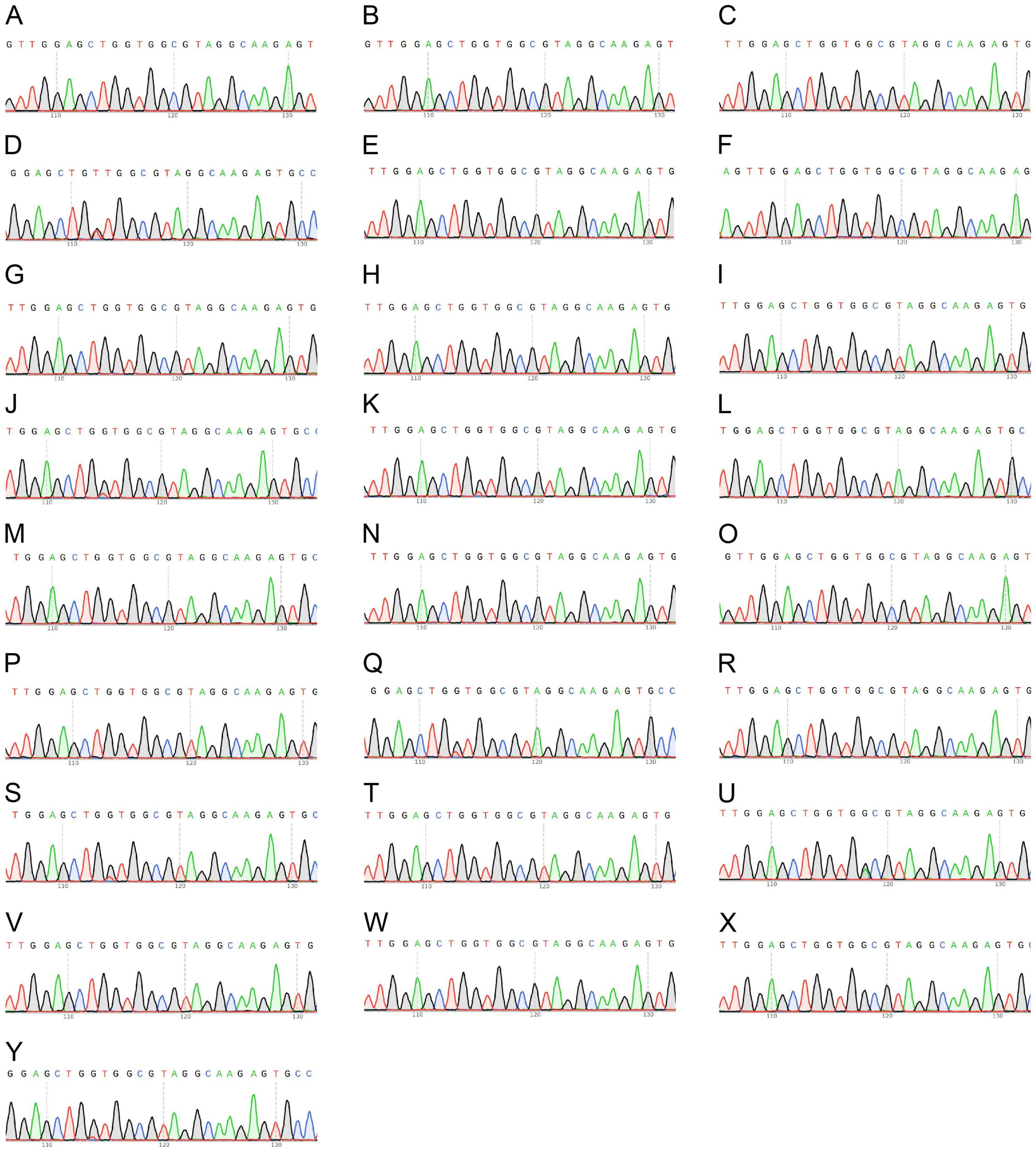

Figure S88. Sanger results for colorectal cancer samples 1-25.

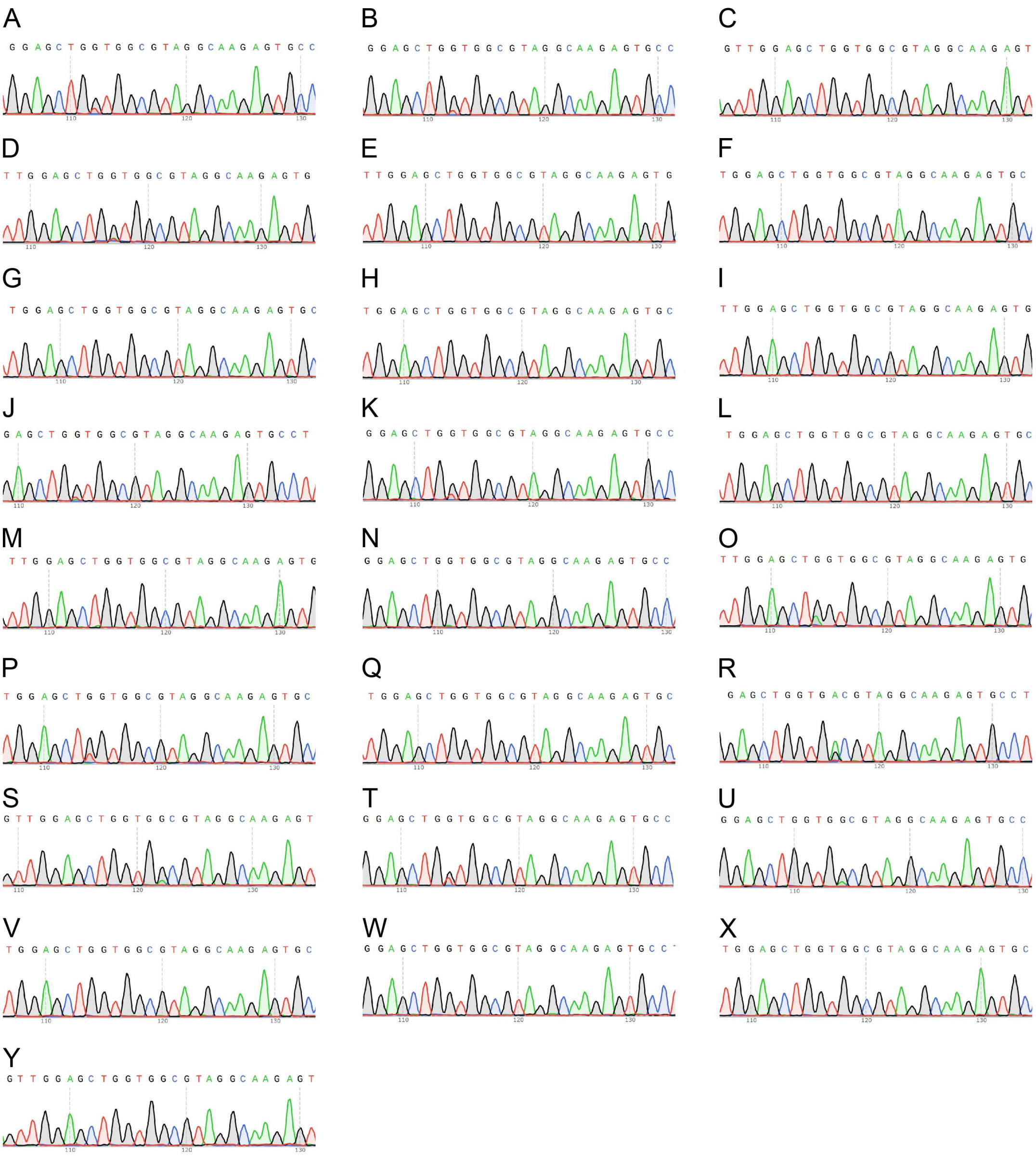


Figure S89. Sanger results for colorectal cancer samples 26-50.

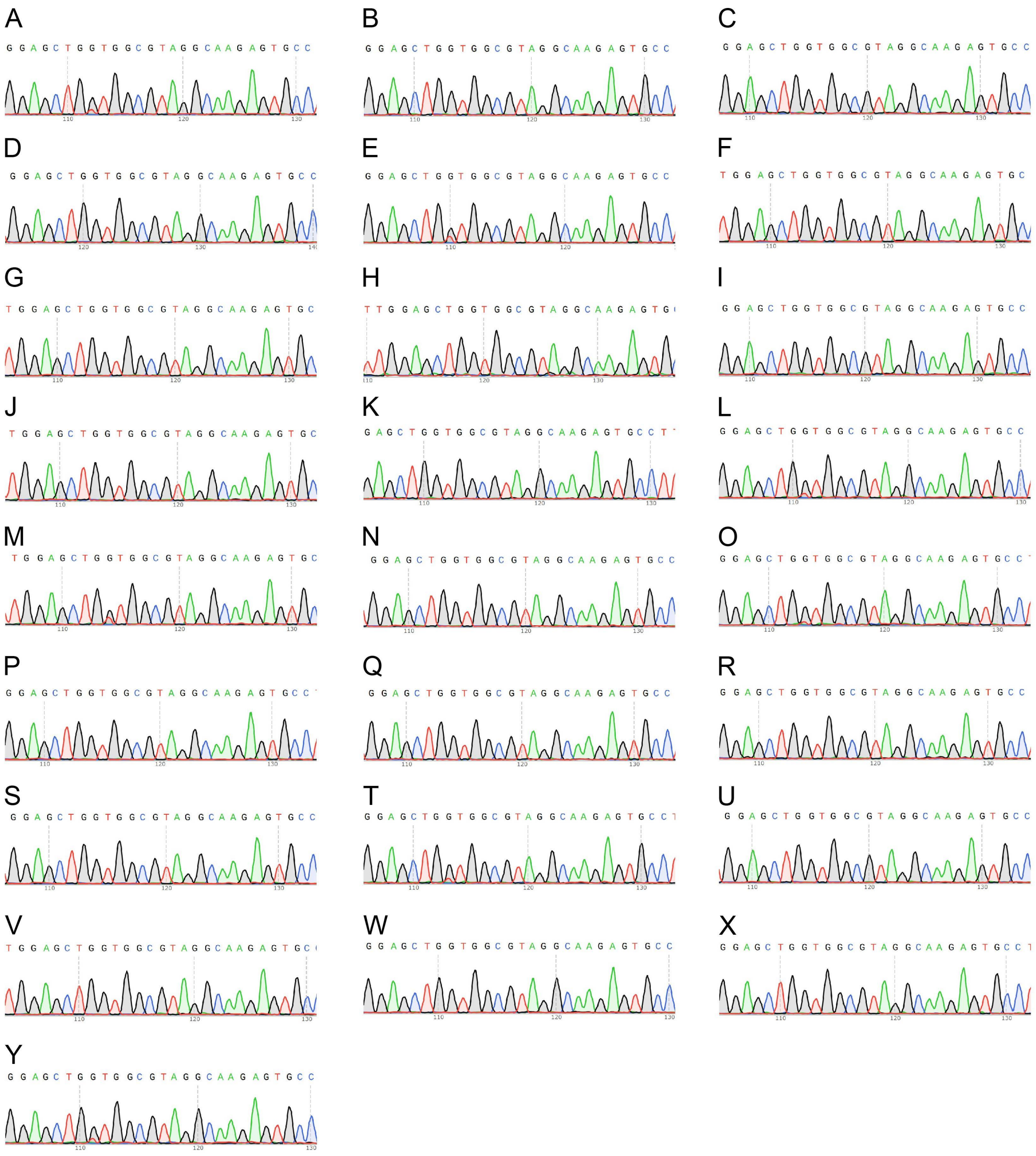


Figure S90. Sanger results for colorectal cancer samples 51-75.

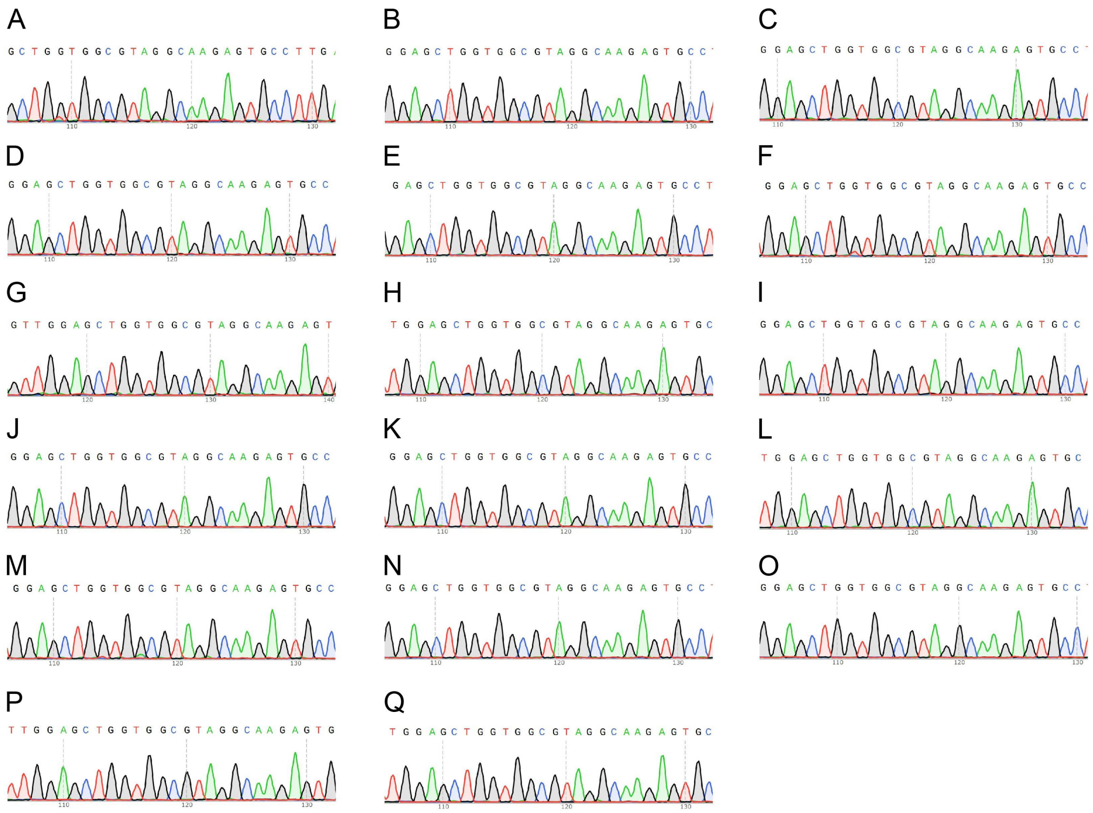


Figure S91. Sanger results for colorectal cancer samples 76-93.

**S2. Supplementary Tables**

Table S1. Sequence.

| | Oligonucleotides | |
|---|---|---|
| tSDR | Probe_F | CGCAGGGTAGGTAGGTAGGGTCGG/ROX/ |
| | Probe_Q | /BHQ2/CCGACCCTACCTACCTACCCTGCG GATGTGAAG |
| | PM_t9 | CTTCACATC CGCAGGGTAGGTAGGTAGGGTCGG |
| | PM_t8 | TTCACATC CGCAGGGTAGGTAGGTAGGGTCGG |
| | PM_t7 | TCACATC CGCAGGGTAGGTAGGTAGGGTCGG |
| | PM_t6 | CACATC CGCAGGGTAGGTAGGTAGGGTCGG |
| | PM_t5 | ACATC CGCAGGGTAGGTAGGTAGGGTCGG |
| | MM2_t9 | CTTCACATG CGCAGGGTAGGTAGGTAGGGTCGG |
| | MM2_t8 | TTCACATG CGCAGGGTAGGTAGGTAGGGTCGG |
| | MM2_t7 | TCACATG CGCAGGGTAGGTAGGTAGGGTCGG |
| | MM2_t6 | CACATG CGCAGGGTAGGTAGGTAGGGTCGG |
| | MM2_t5 | ACATG CGCAGGGTAGGTAGGTAGGGTCGG |
| | MM1_t9 | CTTCACATA CGCAGGGTAGGTAGGTAGGGTCGG |
| | MM1_t8 | TTCACATA CGCAGGGTAGGTAGGTAGGGTCGG |
| | MM1_t7 | TCACATA CGCAGGGTAGGTAGGTAGGGTCGG |
| | MM1_t6 | CACATA CGCAGGGTAGGTAGGTAGGGTCGG |
| | MM1_t6 | ACATA CGCAGGGTAGGTAGGTAGGGTCGG |
| | MM4_t9 | CTTCACATT CGCAGGGTAGGTAGGTAGGGTCGG |
| | MM4_t8 | TTCACATT CGCAGGGTAGGTAGGTAGGGTCGG |
| | MM4_t7 | TCACATT CGCAGGGTAGGTAGGTAGGGTCGG |
| | MM4_t6 | CACATT CGCAGGGTAGGTAGGTAGGGTCGG |
| | MM4_t5 | ACATT CGCAGGGTAGGTAGGTAGGGTCGG |
| | del1-t1_t9 | CTTCACAT CGCAGGGTAGGTAGGTAGGGTCGG |
| | del1-t1_t8 | TTCACAT CGCAGGGTAGGTAGGTAGGGTCGG |
| | del1-t1_t7 | TCACAT CGCAGGGTAGGTAGGTAGGGTCGG |
| | del1-t1_t6 | CACAT CGCAGGGTAGGTAGGTAGGGTCGG |
| | del1-t1_t5 | ACAT CGCAGGGTAGGTAGGTAGGGTCGG |
| | del1-b1_t9 | CTTCACATC GCAGGGTAGGTAGGTAGGGTCGG |
| | del1-b1_t8 | TTCACATC GCAGGGTAGGTAGGTAGGGTCGG |
| | del1-b1_t7 | TCACATC GCAGGGTAGGTAGGTAGGGTCGG |
| | del1-b1_t6 | CACATC GCAGGGTAGGTAGGTAGGGTCGG |
| | del1-b1_t5 | ACATC GCAGGGTAGGTAGGTAGGGTCGG |
| | del2_t9 | CTTCACAT GCAGGGTAGGTAGGTAGGGTCGG |
| | del2_t8 | TTCACAT GCAGGGTAGGTAGGTAGGGTCGG |
| | del2_t7 | TCACAT GCAGGGTAGGTAGGTAGGGTCGG |
| | del2_t6 | CACAT GCAGGGTAGGTAGGTAGGGTCGG |
| | del2_t5 | ACAT GCAGGGTAGGTAGGTAGGGTCGG |
| | | |
| Figure 2 | PM_t11 | TACTTCACATC CCTCGATACTCCGGTCCCGC |

| | | |
|---|---|---|
| | PM_t10 | ACTTCACATC CCTCGATACTCCGGTCCCGC |
| | PM_t9 | CTTCACATC CCTCGATACTCCGGTCCCGC |
| | PM_t8 | TTCACATC CCTCGATACTCCGGTCCCGC |
| | PM_t7 | TCACATC CCTCGATACTCCGGTCCCGC |
| | PM_t6 | CACATC CCTCGATACTCCGGTCCCGC |
| | MM_t11 | TACTTCACATG CCTCGATACTCCGGTCCCGC |
| | MM_t10 | ACTTCACATG CCTCGATACTCCGGTCCCGC |
| | MM_t9 | CTTCACATG CCTCGATACTCCGGTCCCGC |
| | MM_t8 | TTCACATG CCTCGATACTCCGGTCCCGC |
| | MM_t7 | TCACATG CCTCGATACTCCGGTCCCGC |
| | MM_t6 | CACATG CCTCGATACTCCGGTCCCGC |
| αmcSDR | H | GCGGGACCGGAGTATCGAGGCGCAGGGTAGGTAGGTAGGGTCGG |
| | Probe_F | CGCAGGGTAGGTAGGTAGGGTCGG/ROX/ |
| | Probe_Q | /BHQ2/CCGACCCTACCTACCTACCCTGCG TT GATGTGAAGTA |
| | PM_ass16 | TTCACATC CCTCGATACTCCGGTC |
| | MM_ass16 | TTCACATG CCTCGATACTCCGGTC |
| | H_ass16 | GACCGGAGTATCGAGG CGCAGGGTAGGTAGGTAGGGTCGG |
| | PM_ass12 | TTCACATC CCTCGATACTCC |
| | MM_ass12 | TTCACATG CCTCGATACTCC |
| | H_ass12 | GGAGTATCGAGG CGCAGGGTAGGTAGGTAGGGTCGG |
| | PM_ass8 | TTCACATC CCTCGATA |
| | MM_ass8 | TTCACATG CCTCGATA |
| | H_ass8 | TATCGAGG CGCAGGGTAGGTAGGTAGGGTCGG |
| | PM_ass6 | TTCACATC CCTCGA |
| | MM_ass6 | TTCACATG CCTCGA |
| | H_ass6 | TCGAGG CGCAGGGTAGGTAGGTAGGGTCGG |
| | 0T-Probe_Q | /BHQ2/CCGACCCTACCTACCTACCCTGCG GATGTGAAGTA |
| | 1T-Probe_Q | /BHQ2/CCGACCCTACCTACCTACCCTGCG T GATGTGAAGTA |
| | 3T-Probe_Q | /BHQ2/CCGACCCTACCTACCTACCCTGCG TTT GATGTGAAGTA |
| | 4T-Probe_Q | /BHQ2/CCGACCCTACCTACCTACCCTGCG TTTT GATGTGAAGTA |
| | Tspa_PM_t11 | TACTTCACATC TT CCTCGATACTCCGGTCCCGC |
| | Tspa_PM_t10 | ACTTCACATC TT CCTCGATACTCCGGTCCCGC |
| | Tspa_PM_t9 | CTTCACATC TT CCTCGATACTCCGGTCCCGC |
| | Tspa_PM_t8 | TTCACATC TT CCTCGATACTCCGGTCCCGC |
| | Tspa_PM_t7 | TCACATC TT CCTCGATACTCCGGTCCCGC |
| | Tspa_PM_t6 | CACATC TT CCTCGATACTCCGGTCCCGC |

| | | |
|---|---|---|
| | Tspa_MM_t11 | TACTTCACAT TT GCCTCGATACTCCGGTCCCGC |
| | Tspa_MM_t10 | ACTTCACAT TT GCCTCGATACTCCGGTCCCGC |
| | Tspa_MM_t9 | CTTCACAT TT GCCTCGATACTCCGGTCCCGC |
| | Tspa_MM_t8 | TTCACAT TT GCCTCGATACTCCGGTCCCGC |
| | Tspa_MM_t7 | TTCACAT TT GCCTCGATACTCCGGTCCCGC |
| | Tspa_MM_t6 | TCACAT TT GCCTCGATACTCCGGTCCCGC |
| | HBspa | GCGGGACCGGAGTATCGAGGC TT GCAGGGTAGGTAGGTAGGGTCGG |
| | HFspa | GCGGGACCGGAGTATCGAGG TT CGCAGGGTAGGTAGGTAGGGTCGG |
| | H_1 | GCGGGACCGGAGTATCGAGGCGCAGGGTAGGTAGGTAGGGTCG |
| | H_2 | GCGGGACCGGAGTATCGAGGCGCAGGGTAGGTAGGTAGGGTC |
| | H_3 | GCGGGACCGGAGTATCGAGGCGCAGGGTAGGTAGGTAGGGT |
| | H_4 | GCGGGACCGGAGTATCGAGGCGCAGGGTAGGTAGGTAGGG |
| | polyN | AGTAGGAGTAAGAGGGAGATAGGAAGAGAAGGAGATAAGGAAGAAACAAC |
| Figure 3 | | |
| γmcSDR | H | GCGGGACCGGAGTATCGAGGCACAGGGTAGGTAGGTAGGGTCGG |
| | Probe_F | CACAGGGTAGGTAGGTAGGGTCGG/ROX/ |
| | Probe_Q | /BHQ2/CCGACCCTACCTACCTACCCTGTG TT GATGTGAAGTA |
| εmcSDR | H | GCGGGACCGGAGTATCGAGGCATAGGGTAGGTAGGTAGGGTCGG |
| | Probe_F | CATAGGGTAGGTAGGTAGGGTCGG/ROX/ |
| | Probe_Q | /BHQ2/CCGACCCTACCTACCTACCCTATG TT GATGTGAAGTA |
| ηmcSDR | H | GCGGGACCGGAGTATCGAGGCATGGGGTAGGTAGGTAGGGTCGG |
| | Probe_F | CATGGGGTAGGTAGGTAGGGTCGG/ROX/ |
| | Probe_Q | /BHQ2/CCGACCCTACCTACCTACCCCATG TT GATGTGAAGTA |
| λmcSDR | H | GCGGGACCGGAGTATCGAGGCATGTGGTAGGTAGGTAGGGTCGG |
| | Probe_F | CATGTGGTAGGTAGGTAGGGTCGG/ROX/ |
| | Probe_Q | /BHQ2/CCGACCCTACCTACCTACCACATG TT GATGTGAAGTA |
| | | |

| | | |
|---|---|---|
| Type | Tar_A_t8 | TTCACATA CCTCGATACTCCGGTCCCGC |
| | Tar_G_t8 | TTCACATG CCTCGATACTCCGGTCCCGC |
| | Tar_C_t8 | TTCACATC CCTCGATACTCCGGTCCCGC |
| | Tar_T_t8 | TTCACATT CCTCGATACTCCGGTCCCGC |
| αmcSDR | H_A | GCGGGACCGGAGTATCGAGGTGCAGGGTAGGTAGGTAGGGTCGG |
| | H_G | GCGGGACCGGAGTATCGAGGCGCAGGGTAGGTAGGTAGGGTCGG |
| | H_C | GCGGGACCGGAGTATCGAGGGGCAGGGTAGGTAGGTAGGGTCGG |
| | H_T | GCGGGACCGGAGTATCGAGGAGCAGGGTAGGTAGGTAGGGTCGG |
| | Yt_A | TGCAGGGTAGGTAGGTAGGGTCGG |
| | Yt_G | CGCAGGGTAGGTAGGTAGGGTCGG |
| | Yt_C | GGCAGGGTAGGTAGGTAGGGTCGG |
| | Yt_T | AGCAGGGTAGGTAGGTAGGGTCGG |
| | 1Yd_G | CCGACCCTACCTACCTACCCTGCG TT TATGTGAAGTA |
| | 1Yd_C | CCGACCCTACCTACCTACCCTGCC TT TATGTGAAGTA |
| | 1Yd_T | CCGACCCTACCTACCTACCCTGCT TT TATGTGAAGTA |
| | 2Yd_A | CCGACCCTACCTACCTACCCTGCA TT CATGTGAAGTA |
| | 2Yd_C | CCGACCCTACCTACCTACCCTGCC TT CATGTGAAGTA |
| | 2Yd_T | CCGACCCTACCTACCTACCCTGCT TT CATGTGAAGTA |
| | 3Yd_A | CCGACCCTACCTACCTACCCTGCA TT GATGTGAAGTA |
| | 3Yd_G | CCGACCCTACCTACCTACCCTGCG TT GATGTGAAGTA |
| | 3Yd_T | CCGACCCTACCTACCTACCCTGCT TT GATGTGAAGTA |
| | 4Yd_A | CCGACCCTACCTACCTACCCTGCA TT AATGTGAAGTA |
| | 4Yd_G | CCGACCCTACCTACCTACCCTGCG TT AATGTGAAGTA |
| | 4Yd_C | CCGACCCTACCTACCTACCCTGCC TT AATGTGAAGTA |
| | | |
| γmcSDR | H_A | GCGGGACCGGAGTATCGAGGTACAGGGTAGGTAGGTAGGGTCGG |
| | H_G | GCGGGACCGGAGTATCGAGGCACAGGGTAGGTAGGTAGGGTCGG |
| | H_C | GCGGGACCGGAGTATCGAGGGACAGGGTAGGTAGGTAGGGTCGG |
| | H_T | GCGGGACCGGAGTATCGAGGAACAGGGTAGGTAGGTAGGGTCGG |
| | Yt_A | TACAGGGTAGGTAGGTAGGGTCGG |
| | Yt_G | CACAGGGTAGGTAGGTAGGGTCGG |
| | Yt_C | GACAGGGTAGGTAGGTAGGGTCGG |
| | Yt_T | AACAGGGTAGGTAGGTAGGGTCGG |
| | 1Yd_G | CCGACCCTACCTACCTACCCTGTG TT TATGTGAAGTA |
| | 1Yd_C | CCGACCCTACCTACCTACCCTGTC TT TATGTGAAGTA |

| | | |
|---|---|---|
| | 1Yd_T | CCGACCCTACCTACCTACCCTGTT TT TATGTGAAGTA |
| | 2Yd_A | CCGACCCTACCTACCTACCCTGTA TT CATGTGAAGTA |
| | 2Yd_C | CCGACCCTACCTACCTACCCTGTC TT CATGTGAAGTA |
| | 2Yd_T | CCGACCCTACCTACCTACCCTGTT TT CATGTGAAGTA |
| | 3Yd_A | CCGACCCTACCTACCTACCCTGTA TT GATGTGAAGTA |
| | 3Yd_G | CCGACCCTACCTACCTACCCTGTG TT GATGTGAAGTA |
| | 3Yd_T | CCGACCCTACCTACCTACCCTGTT TT GATGTGAAGTA |
| | 4Yd_A | CCGACCCTACCTACCTACCCTGTA TT AATGTGAAGTA |
| | 4Yd_G | CCGACCCTACCTACCTACCCTGTG TT AATGTGAAGTA |
| | 4Yd_C | CCGACCCTACCTACCTACCCTGTC TT AATGTGAAGTA |
| | | |
| εmcSDR | H_A | GCGGGACCGGAGTATCGAGGTATAGGGTAGGTAGGTAGGGTCGG |
| | H_G | GCGGGACCGGAGTATCGAGGCATAGGGTAGGTAGGTAGGGTCGG |
| | H_C | GCGGGACCGGAGTATCGAGGGATAGGGTAGGTAGGTAGGGTCGG |
| | H_T | GCGGGACCGGAGTATCGAGGAATAGGGTAGGTAGGTAGGGTCGG |
| | Yt_A | TATAGGGTAGGTAGGTAGGGTCGG |
| | Yt_G | CATAGGGTAGGTAGGTAGGGTCGG |
| | Yt_C | GATAGGGTAGGTAGGTAGGGTCGG |
| | Yt_T | AATAGGGTAGGTAGGTAGGGTCGG |
| | 1Yd_G | CCGACCCTACCTACCTACCCTATG TT TATGTGAAGTA |
| | 1Yd_C | CCGACCCTACCTACCTACCCTATC TT TATGTGAAGTA |
| | 1Yd_T | CCGACCCTACCTACCTACCCTATT TT TATGTGAAGTA |
| | 2Yd_A | CCGACCCTACCTACCTACCCTATA TT CATGTGAAGTA |
| | 2Yd_C | CCGACCCTACCTACCTACCCTATC TT CATGTGAAGTA |
| | 2Yd_T | CCGACCCTACCTACCTACCCTATT TT CATGTGAAGTA |
| | 3Yd_A | CCGACCCTACCTACCTACCCTATA TT GATGTGAAGTA |
| | 3Yd_G | CCGACCCTACCTACCTACCCTATG TT GATGTGAAGTA |
| | 3Yd_T | CCGACCCTACCTACCTACCCTATT TT GATGTGAAGTA |
| | 4Yd_A | CCGACCCTACCTACCTACCCTATA TT AATGTGAAGTA |
| | 4Yd_G | CCGACCCTACCTACCTACCCTATG TT AATGTGAAGTA |
| | 4Yd_C | CCGACCCTACCTACCTACCCTATC TT AATGTGAAGTA |
| different target | | |
| | IDH1_WT | ATAGGTCGTCATGCTTATGGGGATCAAG |
| | IDH1_R132H | ATAGGTCATCATGCTTATGGGGATCAAG |
| | H_R132H | CTTGATCCCCATAAGCATGACGAAGGGTAGGTAGGTAGGGTCGG |
| | Yt_R132H | CGAAGGGTAGGTAGGTAGGGTCGG |
| | Yd_R132H | CCGACCCTACCTACCTACCCTTCGTTTGACCTATGATG |

| | | |
|---|---|---|
| | R132C | CATAGGTT GTCATGCTTATGGGGATCAA |
| | R132C_WT | CATAGGTC GTCATGCTTATGGGGATCAA |
| | R132C_t9 | TCATAGGTT GTCATGCTTATGGGGATCAA |
| | R132C_WT_t9 | TCATAGGTC GTCATGCTTATGGGGATCAA |
| | H_R132C | TTGATCCCCATAAGCATGAC GACAGGGTAGGTAGGTAGGGTCGG |
| | Yt_R132C | GACAGGGTAGGTAGGTAGGGTCGG |
| | Yd_R132C | CCGACCCTACCTACCTACCCTGTCTTAACCTATGATG |
| | D614 | TATCAGGG TGTTAACTGCACAGAAGTCC |
| | D614_wt | TATCAGGA TGTTAACTGCACAGAAGTCC |
| | D614_t9 | TTATCAGGG TGTTAACTGCACAGAAGTCC |
| | D614_wt_t9 | TTATCAGGA TGTTAACTGCACAGAAGTCC |
| | H_D614 | GGACTTCTGTGCAGTTAACA TCCAGGGTAGGTAGGTAGGGTCGG |
| | Yt_D614 | TCCAGGGTAGGTAGGTAGGGTCGG |
| | εYd_D614 | CCGACCCTACCTACCTACCCTGGA TT CCCTGATAAAG |
| | K417N | CTGGAAAT ATTGCTGATTATAATTATAA |
| | K417N_wt | CTGGAAAG ATTGCTGATTATAATTATAA |
| | H_K417N | TTATAATTATAATCAGCAAT CTTAGGGTAGGTAGGTAGGGTCGG |
| | Yt_K417N | CTTAGGGTAGGTAGGTAGGGTCGG |
| | Yd_K417N | CCGACCCTACCTACCTACCCTAAG TT ATTTCCAGTTT |
| | K417T | ACTGGAAC GATTGCTGATTATAATTATA |
| | K417T_wt | ACTGGAAA GATTGCTGATTATAATTATA |
| | H_K417T | TATAATTATAATCAGCAATC TTTAGGGTAGGTAGGTAGGGTCGG |
| | Yt_K417T | TTTAGGGTAGGTAGGTAGGGTCGG |
| | Yd_K417T | CCGACCCTACCTACCTACCCTAAA TT GTTCCAGTTTG |
| | N501Y | ACCCACTT ATGGTGTTGGTTACCAACCA |
| | N501Y_wt | ACCCACTA ATGGTGTTGGTTACCAACCA |
| | H_N501Y | TGGTTGGTAACCAACACCAT TAGAGaGTAGGTAGGTAGGGTCGG |
| | Yt_N501Y | TAGAGaGTAGGTAGGTAGGGTCGG |
| | Yd_N501Y | CCGACCCTACCTACCTACtCTCTA TT AAGTGGGTTGG |
| | E484A | GGTGTTGC AGGTTTTAATTGTTACTTTC |
| | E484A_wt | GGTGTTGA AGGTTTTAATTGTTACTTTC |
| | H_E484A | GAAAGTAACAATTAAAACCT TCAAGGGTAGGTAGGTAGGGTCGG |
| | Yt_E484A | TCAAGGGTAGGTAGGTAGGGTCGG |
| | Yd_E484A | CCGACCCTACCTACCTACCCTTGA TT GCAACACCATT |
| | E484K | TGGTGTTA AAGGTTTTAATTGTTACTTT |
| | E484K_wt | TGGTGTTG AAGGTTTTAATTGTTACTTT |

| | | |
|---|---|---|
| | H_E484K | AAAGTAACAATTAAAACCTT CAAAGGGTAGGTAGGTAGGGTCGG |
| | Yt_E484K | CAAAGGGTAGGTAGGTAGGGTCGG |
| | Yd_E484K | CCGACCCTACCTACCTACCCTTTG TT TAACACCATTA |
| | H655Y | GGCTGAAT ATGTCAACAACTCATATGAG |
| | H655Y_wt | GGCTGAAC ATGTCAACAACTCATATGAG |
| | H655Y_t9 | GGGCTGAAT ATGTCAACAACTCATATGAG |
| | H655Y_wt_t9 | GGGCTGAAC ATGTCAACAACTCATATGAG |
| | H_H655Y | CTCATATGAGTTGTTGACAT GTTAGGGTAGGTAGGTAGGGTCGG |
| | Yt_H655Y | GTTAGGGTAGGTAGGTAGGGTCGG |
| | Yd_H655Y | CCGACCCTACCTACCTACCCTAACTAATTCAGCCCCT |
| | S371F | TATAATTT CGCATCATTTTCCACTTTTA |
| | S371F_wt | TATAATTC CGCATCATTTTCCACTTTTA |
| | S371F_t9 | ATATAATTT CGCATCATTTTCCACTTTTA |
| | S371F_wt_t9 | ATATAATTC CGCATCATTTTCCACTTTTA |
| | S371F_t10 | TATATAATTT CGCATCATTTTCCACTTTTA |
| | S371F_wt_t10 | TATATAATTC CGCATCATTTTCCACTTTTA |
| | S371F_t11 | CTATATAATTT CGCATCATTTTCCACTTTTA |
| | S371F_wt_t11 | CTATATAATTC CGCATCATTTTCCACTTTTA |
| | H_S371F | TAAAAGTGGAAAATGATGCG GAAAGGGTAGGTAGGTAGGGTCGG |
| | Yt_S371F | GAAAGGGTAGGTAGGTAGGGTCGG |
| | Yd_S371F | CCGACCCTACCTACCTACCCTTTCTTAAATTATATAG |
| | L858R | TTTGGGCG GGCCAAACTGCTGGGTGCGG |
| | L858R_wt | TTTGGGCT GGCCAAACTGCTGGGTGCGG |
| | L858R_t9 | TTTTGGGCG GGCCAAACTGCTGGGTGCGG |
| | L858R_wt_t9 | TTTTGGGCT GGCCAAACTGCTGGGTGCGG |
| | H_L858R | CCGCACCCAGCAGTTTGGCC AGCAGGGTAGGTAGGTAGGGTCGG |
| | Yt_L858R | AGCAGGGTAGGTAGGTAGGGTCGG |
| | Yd_L858R | CCGACCCTACCTACCTACCCTGCTTTCGCCCAAAATC |
| | T790M | CTCATCAC GCAGCTCATGCCCTTCGGCT |
| | T790M_wt | CTCATCAT GCAGCTCATGCCCTTCGGCT |
| | T790M_t9 | GCTCATCAC GCAGCTCATGCCCTTCGGCT |
| | T790M_wt_t9 | GCTCATCAT GCAGCTCATGCCCTTCGGCT |
| | H_T790M | AGCCGAAGGGCATGAGCTGC ATGAGGGTAGGTAGGTAGGGTCGG |
| | Yt_T790M | ATGAGGGTAGGTAGGTAGGGTCGG |
| | Yd_T790M | CCGACCCTACCTACCTACCCTCATTTGTGATGAGCTG |
| | G12CSR_t7 | GGAGCTG GTGGCGTAGGCAAGAGTGCC |

| | | |
|---|---|---|
| | G12CSR | TGGAGCTG GTGGCGTAGGCAAGAGTGCC |
| | G12CSR_t9 | TTGGAGCTG GTGGCGTAGGCAAGAGTGCC |
| | H_G12CSR | GGCACTCTTGCCTACGCCAC CAGAGGGTAGGTAGGTAGGGTCGG |
| | G12R | TGGAGCTC GTGGCGTAGGCAAGAGTGCC |
| | G12R_t9 | TTGGAGCTC GTGGCGTAGGCAAGAGTGCC |
| | G12C_t7 | GGAGCTT GTGGCGTAGGCAAGAGTGCC |
| | G12C | TGGAGCTT GTGGCGTAGGCAAGAGTGCC |
| | G12S | TGGAGCTA GTGGCGTAGGCAAGAGTGCC |
| | Yt_G12CSR | CAGAGGGTAGGTAGGTAGGGTCGG |
| | Yd_G12R | CCGACCCTACCTACCTACCCTCTG TT GAGCTCCAACT |
| | Yd_G12C | CCGACCCTACCTACCTACCCTCTG TT AAGCTCCAACT |
| | Yd_G12S | CCGACCCTACCTACCTACCCTCTG TT TAGCTCCAACT |
| | G12VDA_t7 | GAGCTGG TGGCGTAGGCAAGAGTGCCT |
| | G12VDA | GGAGCTGG TGGCGTAGGCAAGAGTGCCT |
| | G12A_t7 | GAGCTGC TGGCGTAGGCAAGAGTGCCT |
| | G12A | GGAGCTGC TGGCGTAGGCAAGAGTGCCT |
| | G12D_t7 | GAGCTGA TGGCGTAGGCAAGAGTGCCT |
| | G12D | GGAGCTGA TGGCGTAGGCAAGAGTGCCT |
| | G12V_t7 | GAGCTGT TGGCGTAGGCAAGAGTGCCT |
| | G12V | GGAGCTGT TGGCGTAGGCAAGAGTGCCT |
| | H_G12DA | AGGCACTCTTGCCTACGCCA CCAAGGGTAGGTAGGTAGGGTCGG |
| | H_G12V | AGGCACTCTTGCCTACGCCA CCAAGTGaAGTGAGTGAGGGTCGG |
| | Yt_G12DA | CCAAGGGTAGGTAGGTAGGGTCGG |
| | Yd_G12A | CCGACCCTACCTACCTACCCTTGG TT GCAGCTCCAAC |
| | Yd_G12D | CCGACCCTACCTACCTACCCTTGG TT TCAGCTCCAAC |
| | Yt_G12V | CCAAGTGAAGTGAGTGAGGGTCGG |
| | Yd_G12V | CCGACCCTCACTCACTTCACTTGG TT ACAGCTCCAAC |
| | G13CS | AGCTGGTG GCGTAGGCAAGAGTGCCTTG |
| | G13C | AGCTGGTT GCGTAGGCAAGAGTGCCTTG |
| | H_G13C | CAAGGCACTCTTGCCTACGC CACAGGGTAGGTAGGTAGGGTCGG |
| | Yt_G13C | CACAGGGTAGGTAGGTAGGGTCGG |
| | Yd_G13C | CCGACCCTACCTACCTACCCTGTG TT AACCAGCTCCA |
| | G13S | AGCTGGTA GCGTAGGCAAGAGTGCCTTG |
| | H_G13S | CAAGGCACTCTTGCCTACGC CACAtGGTAGGTAGGTAGGGTCGG |
| | Yt_G13S | CACAtGGTAGGTAGGTAGGGTCGG |
| | Yd_G13S | CCGACCCTACCTACCTACCaTGTG AA TACCAGCTCCA |
| | G13VD_t7 | CTGGTGG CGTAGGCAAGAGTGCCTTGA |
| | G13VD | GCTGGTGG CGTAGGCAAGAGTGCCTTGA |

| | | |
|---|---|---|
| | G13D_t7 | CTGGTGA CGTAGGCAAGAGTGCCTTGA |
| | G13D | GCTGGTGA CGTAGGCAAGAGTGCCTTGA |
| | H_G13D | TCAAGGCACTCTTGCCTACG<br>CCAAGGGTAGGTAGGTAGGGTCGG |
| | Yt_G13D | CCAAGGGTAGGTAGGTAGGGTCGG |
| | Yd_G13D | CCGACCCTACCTACCTACCCTTGG TT TCACCAGCTCC |
| | G13V | GCTGGTGT CGTAGGCAAGAGTGCCTTGA |
| | H_G13V | TCAAGGCACTCTTGCCTACG<br>CCAAGTGaAGTGAGTGAGcGTCcG |
| | Yt_G13V | CCAAGTGaAGTGAGTGAGcGTCcG |
| | Yd_G13V | CGGACGCTCACTCACTTCACTTGG TT ACACCAGCTCC |
| | Q61H | CAGGTCAC GAGGAGTACAGTGCAATGAG |
| | Q61H_wt | CAGGTCAA GAGGAGTACAGTGCAATGAG |
| | H_Q61H | CTCATTGCACTGTACTCCTC<br>TTGAGGGTAGGTAGGTAGGGTCGG |
| | Yt_Q61H | TTGAGGGTAGGTAGGTAGGGTCGG |
| | Yd_Q61H | CCGACCCTACCTACCTACCCTCAA TT GTGACCTGCTG |
| | SMAD7 | GAGGAAAC AGGACCCCAGAGCTCCCTCA |
| | SMAD7_wt | GAGGAAAT AGGACCCCAGAGCTCCCTCA |
| | H_SMAD7 | TGAGGGAGCTCTGGGGTCCT<br>ATTAGGGTAGGTAGGTAGGGTCGG |
| | Yt_SMAD7 | ATTAGGGTAGGTAGGTAGGGTCGG |
| | Yd_SMAD7 | CCGACCCTACCTACCTACCCTAAT TAG TTTCCTCTTT |
| | D594G | AAAGGTGATTTTGGTCTAGCTACAGTGA |
| | D594G_wt | AAAGGTGGTTTTGGTCTAGCTACAGTGA |
| | H_D594G | TCACTGTAGCTAGACCAAAA<br>TCAAGGGTAGGTAGGTAGGGTCGG |
| | Yt_D594G | TCAAGGGTAGGTAGGTAGGGTCGG |
| | Yd_D594G | CCGACCCTACCTACCTACCCTTGA TT CCACCTTTTTT |
| | H1047L | CTTGCACATCATGGTGGCTGGAAAGATT |
| | H1047L_wt | CTTGCACTTCATGGTGGCTGGAAAGATT |
| | H_H1047L | AATCTTTCCAGCCACCATGA<br>TGTAGGGTAGGTAGGTAGGGTCGG |
| | Yt_H1047L | TGTAGGGTAGGTAGGTAGGGTCGG |
| | Yd_H1047L | CCGACCCTACCTACCTACCCTACA aT AGTGCAAGAGA |
| | DNMT1 | CAGGGGTCACGGGAGGGCAGAACTAGTC |
| | DNMT1_wt | CAGGGGTGACGGGAGGGCAGAACTAGTC |
| | H_DNMT1 | GACTAGTTCTGCCCTCCCGT<br>CACAGGGTAGGTAGGTAGGGTCGG |
| | Yt_DNMT1 | CACAGGGTAGGTAGGTAGGGTCGG |
| | Yd_DNMT1 | CCGACCCTACCTACCTACCCTGTG TT GACCCCTGTTT |
| | IVS564 | GTTAAGGTAATAGCAATATCTCTGCATA |
| | IVS564_wt | GTTAAGGCAATAGCAATATCTCTGCATA |

| | | |
|---|---|---|
| | IVS564_t9 | GGTTAAGGTAATAGCAATATCTCTGCATA |
| | IVS564_wt_t9 | GGTTAAGGCAATAGCAATATCTCTGCATA |
| | H_ IVS564 | TATGCAGAGATATTGCTATT GCCAGGGTAGGTAGGTAGGGTCGG |
| | Yt_ IVS564 | GCCAGGGTAGGTAGGTAGGGTCGG |
| | Yd_IVS564 | CCGACCCTACCTACCTACCCTGGCTTACCTTAACCCA |
| Figure 4 and 5 | | |
| | z-IDH1-wt | GTTCAAGTTGAAACAAATGTGGAAATCACCAAATGGCACCATACGAAATATTCTGGGTGGCACGGTCTTCAGAGAAGCCATTATCTGCAAAAATATCCCCCGGCTTGTGAGTGGATGGGTAAAACCTATCATCATAGGTCGTCATGCTTATGGGGATCAAGTAAGTCATGTTGGCAATAATGTGATTTTGCATGTTTTTTTTTTCATGGCCCAGAAATTTCCAACTTGTATGTGTTTTATTCTTATCTTTTGGTATCTACACCCATTAAGCAAGGTATGAAATTG |
| | z-IDH1-R132H | GTTCAAGTTGAAACAAATGTGGAAATCACCAAATGGCACCATACGAAATATTCTGGGTGGCACGGTCTTCAGAGAAGCCATTATCTGCAAAAATATCCCCCGGCTTGTGAGTGGATGGGTAAAACCTATCATCATAGGTCATCATGCTTATGGGGATCAAGTAAGTCATGTTGGCAATAATGTGATTTTGCATGTTTTTTTTTTCATGGCCCAGAAATTTCCAACTTGTATGTGTTTTATTCTTATCTTTTGGTATCTACACCCATTAAGCAAGGTATGAAATTG |
| | z-E484_wt | AATTCTAACAATCTTGATTCTAAGGTTGGTGGTAATTATAATTACCTGTATAGATTGTTTAGGAAGTCTAATCTCAAACCTTTTGAGAGAGATATTTCAACTGAAATCTATCAGGCCGGTAGCACACCTTGTAATGGTGTTGAAGGTTTTAATTGTTACTTTCCTTTACAATCATATGGTTTCCAACCCACTAATGGTGTTGGTTACCAACCATACAGAGTAGTAGTACTTTCTTTTGAACTTCTACATGCACCAGCAACTGTTTGTGGACCTAAAAAGTCTACT |
| | z-E484A | AATTCTAACAATCTTGATTCTAAGGTTGGTGGTAATTATAATTACCTGTATAGATTGTTTAGGAAGTCTAATCTCAAACCTTTTGAGAGAGATATTTCAACTGAAATCTATCAGGCCGGTAGCACACCTTGTAATGGTGTTGCAGGTTTTAATTGTTACTTTCCTTTACAATCATATGGTTTCCAACCCACTAATGGTGTTGGTTACCAACCATACAGAGTAGTAGTACTTTCTTTTGAACTTCTACATGCACCAGCAACTGTTTGTGGACCTAAAAAGTCTACT |
| | z-KRAS_wt | GTATTAAAAGGTACTGGTGGAGTATTTGATAGTGTATTAACCTTATGTGTGACATGTTCTAATATAGTCACATTTTCATTATTTTTATTATAAGGCCTGCTGAAAATGACTGAATATAAACTTGTGGTAGTTGGAGCTGGTGGCGTAGGCAAGAGTGCCTTGACGATACAGCTAATTCAGAATCATTTTGTGGACGAATATGATCCAACAATAGAGGTAAATCTTGTTTTAATATGCATATTACTGGTGCAGGACCATTCTTTGATACAGATAAAGGTTT |

| | | |
|---|---|---|
| | z-KRAS_G12V | GTATTAAAAGGTACTGGTGGAGTATTTGATAGTGTATTAACC TTATGTGTGACATGTTCTAATATAGTCACATTTTCATTATTTTT ATTATAAGGCCTGCTGAAAATGACTGAATATAAACTTGTGGT AGTTGGAGCTGTTGGCGTAGGCAAGAGTGCCTTGACGATAC AGCTAATTCAGAATCATTTTGTGGACGAATATGATCCAACAAT AGAGGTAAATCTTGTTTTAATATGCATATTACTGGTGCAGGA CCATTCTTTGATACAGATAAAGGTTT |
| | IDH1-FP | GTGGCACGGTCTTCAGAGAA |
| | IDH1-RP | p-CCAACATGACTTACTTGATCCCC |
| | R132H-O | TAGGTTTTACCCATCCACTCACAAGCCGGG |
| | Yd_R132H-t8 | /BHQ2/CCGACCCTACCTACCTACCCTTCGT TT GACCTAT |
| | E484-FP | GCCGGTAGCACACCTTGTAAT |
| | E484-RP | p-CAGTTGCTGGTGCATGTAGA |
| | E484-O | AAGGTGTGCTACCGGCCTGATAGATTTCAG |
| | Yd_E484A-t8 | /BHQ2/CCGACCCTACCTACCTACCCTTGA TT GCAACACC |
| | G12V-FP | AGGCCTGCTGAAAATGACTGA |
| | G12V-RP | p-AGAATGGTCCTGCACCAGTAA |
| | G12V-O2 | CAACTACCACAAGTTTATATTCAGTCATTTTCAGC |
| | Yd_G12V-t7 | /BHQ2/CCGACCCTCACTCACTTCACTTGG TT ACAGCTC |
| For muti | | |
| | Yt_R132H | CGAAGGGTAGGTAGGTAGGGTCGG/Cy5/ |
| | Yd_R132H-t8 | /BHQ3/CCGACCCTACCTACCTACCCTTCGT TT GACCTAT |
| | Yt_E484A | TCAAGGGTAGGTAGGTAGGGTCGG/HEX/ |
| | Yd_E484A-t8 | /BHQ1/CCGACCCTACCTACCTACCCTTGA TT GCAACACC |
| | Yt_G12VDA | CCAAGTGAAGTGAGTGAGGGTCGG/ROX/ |
| | Yd_G12V-t7 | /BHQ2/CCGACCCTCACTCACTTCACTTGG TT ACAGCTC |
| Primer for Sanger | | |
| | R132H-213F | AAATCACCAAATGGCACCATA |
| | R132H-627R | ACCAACATAAAAATGGCAAT |
| | G12V-188F | TTTGTATTAAAAGGTACTGGTGG |
| | G12V-570R | AGGAAAGTAAAGTTCCCAT |

Table S2. A comparison of the mutation detection technologies.

| Method | Principle | Mutation LoD (%) | Working time (h) | Multiplexity | Ref. |
|---|---|---|---|---|---|
| Microarray (MIP) | PCR+Hybridization | 1-10 | 16+ | high | (7) |
| ARMS PCR | PCR | 1-10 | 2 | low | (8) |
| NGS | Sequencing | 1 | 48+ | high | (9) |
| Digital PCR | PCR | 0.05-0.1 | 3 | low | (10) |
| NanoSeq | Sequencing | 0.01 | 48+ | high | (11) |
| competitive DNA probe system and DSN digestion | Enzyme digestion +SDR | 1 | 3 | low | (12) |
| BDA | SDR+PCR | 0.1 | 2 | low | (13) |
| PANDA | PCR+SDR+ | 0.1 | 2 | low | (14) |
| mcSDR | PCR+SDR | 0.1 | 2 | low | This work |

Table S3. Patient information for IDH1.

| Sample (No.) | Patient (No.) | Sex | Age | Disease |
|---|---|---|---|---|
| 1 | 58 | Man | 34 | astrocytomas |
| 2 | 14 | Man | 59 | oligodendrogliomas |
| 3 | 54 | Women | 55 | astrocytomas |
| 4 | 27 | Women | 69 | glioblastoma |
| 5 | 20 | Women | 66 | astrocytomas |
| 6 | 17 | Man | 49 | oligodendrogliomas |
| 7 | 29 | Man | 52 | glioblastoma |
| 8 | 61 | Man | 60 | astrocytomas |
| 9 | 11 | Man | 21 | glioblastoma |
| 10 | 69 | Man | 50 | glioblastoma |
| 11 | 37 | Women | 61 | glioblastoma |
| 12 | 34 | Women | 51 | astrocytomas |
| 13 | 84 | Man | 53 | glioblastoma |
| 14 | 67 | Man | 57 | glioblastoma |
| 15 | 76 | Women | 68 | glioblastoma |
| 16 | 47 | Man | 42 | astrocytomas |
| 17 | 28 | Women | 37 | glioblastoma |
| 18 | 39 | Man | 31 | astrocytomas |
| 19 | 50 | Man | 70 | glioblastoma |
| 20 | 70 | Man | 69 | glioblastoma |
| 21 | 15 | Man | 42 | oligodendrogliomas |
| 22 | 32 | Man | 67 | glioblastoma |
| 23 | 21 | Women | 59 | glioblastoma |
| 24 | 82 | Women | 43 | astrocytomas |
| 25 | 10 | Women | 47 | glioblastoma |
| 26 | 45 | Women | 37 | glioblastoma |
| 27 | 19 | Women | 54 | glioblastoma |
| 28 | 59 | Man | 51 | glioblastoma |
| 29 | 12 | Women | 53 | oligodendrogliomas |
| 30 | 62 | Man | 50 | oligodendrogliomas |
| 31 | 56 | Women | 61 | glioblastoma |
| 32 | 52 | Man | 73 | glioblastoma |
| 33 | 72 | Women | 71 | glioblastoma |
| 34 | 18 | Man | 72 | glioblastoma |
| 35 | 65 | Man | 53 | astrocytomas |
| 36 | 9 | Man | 41 | oligodendrogliomas |
| 37 | 71 | Man | 38 | astrocytomas |
| 38 | 60 | Women | 52 | oligodendrogliomas |
| 39 | 31 | Man | 61 | glioblastoma |
| 40 | 6 | Women | 58 | glioblastoma |

| | | | | |
|---|---|---|---|---|
| 41 | 23 | Women | 74 | glioblastoma |
| 42 | 22 | Women | 74 | glioblastoma |
| 43 | 80 | Man | 62 | glioblastoma |
| 44 | 35 | Man | 52 | oligodendrogliomas |
| 45 | 78 | Women | 53 | oligodendrogliomas |
| 46 | 8 | Women | 60 | glioblastoma |
| 47 | 68 | Women | 15 | astrocytomas |
| 48 | 38 | Women | 64 | glioblastoma |
| 49 | 2 | Women | 38 | oligodendrogliomas |
| 50 | 13 | Women | 51 | glioblastoma |
| 51 | 41 | Women | 47 | oligodendrogliomas |
| 52 | 4 | Women | 49 | glioblastoma |
| 53 | 36 | Man | 61 | glioblastoma |
| 54 | 33 | Man | 76 | glioblastoma |
| 55 | 26 | Man | 51 | glioblastoma |
| 56 | 57 | Man | 47 | astrocytomas |
| 57 | 64 | Man | 56 | oligodendrogliomas |
| 58 | 90 | Man | 62 | glioblastoma |
| 59 | 30 | Women | 58 | glioblastoma |
| 60 | 86 | Man | 67 | glioblastoma |
| 61 | 73 | Women | 34 | glioblastoma |
| 62 | 16 | Women | 56 | oligodendrogliomas |
| 63 | 5 | Women | 40 | oligodendrogliomas |
| 64 | 25 | Man | 28 | oligodendrogliomas |
| 65 | 43 | Man | 44 | glioblastoma |
| 66 | 55 | Women | 53 | glioblastoma |
| 67 | 24 | Man | 68 | glioblastoma |
| 68 | 7 | Women | 32 | astrocytomas |
| 69 | 1 | Women | 43 | oligodendrogliomas |
| 70 | 3 | Women | 58 | glioblastoma |
| 71 | 74 | Women | 70 | glioblastoma |
| 72 | 40 | Man | 56 | astrocytomas |
| 73 | 75 | Man | 72 | glioblastoma |
| 74 | 42 | Man | 66 | oligodendrogliomas |
| 75 | 77 | Man | 68 | glioblastoma |
| 76 | 79 | Women | 48 | glioblastoma |
| 77 | 44 | Women | 24 | astrocytomas |
| 78 | 46 | Man | 30 | oligodendrogliomas |
| 79 | 81 | Man | 81 | glioblastoma |
| 80 | 48 | Women | 33 | astrocytomas |
| 81 | 83 | Women | 37 | glioblastoma |
| 82 | 49 | Women | 37 | astrocytomas |
| 83 | 51 | Man | 45 | astrocytomas |

| | | | | |
|---|---|---|---|---|
| 84 | 85 | Women | 70 | glioblastoma |
| 85 | 87 | Women | 69 | glioblastoma |
| 86 | 53 | Women | 36 | oligodendrogliomas |
| 87 | 88 | Man | 59 | glioblastoma |
| 88 | 89 | Man | 31 | glioblastoma |
| 89 | 63 | Man | 56 | astrocytomas |
| 90 | 94 | Women | 60 | glioblastoma |
| 91 | 66 | Women | 58 | oligodendrogliomas |
| 92 | 92 | Man | 70 | glioblastoma |
| 93 | 93 | Women | 34 | oligodendrogliomas |
| 94 | 91 | Women | 36 | oligodendrogliomas |
| 95 | 95 | Man | 54 | oligodendrogliomas |

Table S4. Patient information for KRAS.

| Sample (No.) | Patient (No.) | Sex | Age | Disease |
|---|---|---|---|---|
| 1 | 19 | Man | 68 | Malignant Rectal Tumor |
| 2 | 50 | Man | 70 | Malignant Rectal Tumor |
| 3 | 51 | Man | 73 | Malignant Colonic Tumor |
| 4 | 38 | Man | 56 | Malignant Rectal Tumor |
| 5 | 62 | Woman | 51 | Malignant Colonic Tumor |
| 6 | 63 | Man | 36 | Malignant Colonic Tumor |
| 7 | 58 | Woman | 62 | Malignant Colonic Tumor |
| 8 | 1 | Man | 59 | Malignant Rectal Tumor |
| 9 | 33 | Man | 45 | Malignant Rectal Tumor |
| 10 | 41 | Woman | 70 | Malignant Colonic Tumor |
| 11 | 7 | Woman | 68 | Malignant Rectal Tumor |
| 12 | 47 | Man | 75 | Malignant Colonic Tumor |
| 13 | 14 | Man | 50 | Malignant Colonic Tumor |
| 14 | 12 | Man | 60 | Malignant Rectal Tumor |
| 15 | 31 | Man | 55 | Malignant Rectal Tumor |
| 16 | 5 | Man | 83 | Malignant Rectal Tumor |
| 17 | 23 | Man | 60 | Malignant Colonic Tumor |
| 18 | 48 | Man | 70 | Malignant Colonic Tumor |
| 19 | 37 | Woman | 60 | Malignant Rectal Tumor |
| 20 | 55 | Man | 50 | Malignant Rectal Tumor |
| 21 | 25 | Woman | 60 | Malignant Rectal Tumor |
| 22 | 45 | Man | 79 | Malignant Colonic Tumor |
| 23 | 18 | Man | 44 | Malignant Rectal Tumor |
| 24 | 8 | Woman | 50 | Malignant Colonic Tumor |
| 25 | 53 | Man | 69 | Malignant Colonic Tumor |
| 26 | 44 | Woman | 56 | Malignant ileal tumor |
| 27 | 29 | Woman | 50 | Malignant Rectal Tumor |
| 28 | 36 | Man | 50 | Malignant Colonic Tumor |
| 29 | 34 | Woman | 52 | Malignant Rectal Tumor |
| 30 | 27 | Man | 75 | Malignant Colonic Tumor |
| 31 | 39 | Man | 68 | Malignant Rectal Tumor |
| 32 | 57 | Woman | 72 | Malignant Colonic Tumor |
| 33 | 59 | Man | 60 | Malignant Rectal Tumor |
| 34 | 17 | Woman | 55 | Malignant Rectal Tumor |
| 35 | 10 | Man | 79 | Malignant Rectal Tumor |
| 36 | 91 | Woman | 80 | Malignant Colonic Tumor |
| 37 | 83 | Woman | 84 | Malignant Colonic Tumor |
| 38 | 70 | Woman | 72 | Malignant Colonic Tumor |
| 39 | 11 | Man | 75 | Malignant Colonic Tumor |
| 40 | 61 | Woman | 55 | Malignant Rectal Tumor |

| | | | | |
|---|---|---|---|---|
| 41 | 46 | Man | 69 | Malignant Rectal Tumor |
| 42 | 6 | Man | 61 | Malignant Rectal Tumor |
| 43 | 2 | Woman | 67 | Malignant Colonic Tumor |
| 44 | 56 | Woman | 61 | Malignant Rectal Tumor |
| 45 | 67 | Man | 59 | Malignant Colonic Tumor |
| 46 | 60 | Man | 80 | Malignant Colonic Tumor |
| 47 | 4 | Woman | 65 | Malignant Rectal Tumor |
| 48 | 82 | Woman | 55 | Malignant Colonic Tumor |
| 49 | 80 | Man | 84 | Malignant Colonic Tumor |
| 50 | 22 | Man | 69 | Malignant Colonic Tumor |
| 51 | 32 | Man | 76 | Malignant Rectal Tumor |
| 52 | 16 | Man | 69 | Malignant Colonic Tumor |
| 53 | 26 | Woman | 47 | Malignant Rectal Tumor |
| 54 | 28 | Woman | 57 | Malignant Colonic Tumor |
| 55 | 85 | Woman | 60 | Malignant Rectal Tumor |
| 56 | 21 | Man | 59 | Malignant Rectal Tumor |
| 57 | 13 | Man | 73 | Malignant Rectal Tumor |
| 58 | 15 | Woman | 70 | Malignant Colonic Tumor |
| 59 | 20 | Man | 71 | Malignant Rectal Tumor |
| 60 | 68 | Woman | 83 | Malignant Colonic Tumor |
| 61 | 35 | Man | 48 | Malignant Colonic Tumor |
| 62 | 65 | Man | 53 | Malignant Colonic Tumor |
| 63 | 49 | Woman | 70 | Malignant Rectal Tumor |
| 64 | 69 | Man | 80 | Malignant Colonic Tumor |
| 65 | 42 | Man | 58 | Malignant Colonic Tumor |
| 66 | 52 | Woman | 59 | Malignant Rectal Tumor |
| 67 | 43 | Man | 60 | Malignant Colonic Tumor |
| 68 | 24 | Woman | 32 | Malignant Colonic Tumor |
| 69 | 54 | Man | 63 | Malignant Colonic Tumor |
| 70 | 40 | Woman | 81 | Malignant Rectal Tumor |
| 71 | 30 | Woman | 53 | Malignant Rectal Tumor |
| 72 | 92 | Woman | 74 | Malignant Colonic Tumor |
| 73 | 73 | Man | 55 | Malignant Rectal Tumor |
| 74 | 72 | Woman | 73 | Malignant Colonic Tumor |
| 75 | 75 | Man | 88 | Malignant Rectal Tumor |
| 76 | 86 | Man | 60 | Malignant Colonic Tumor |
| 77 | 90 | Woman | 70 | Malignant Colonic Tumor |
| 78 | 71 | Man | 77 | Malignant Rectal Tumor |
| 79 | 87 | Man | 56 | Malignant Rectal Tumor |
| 80 | 76 | Woman | 77 | Malignant Colonic Tumor |
| 81 | 84 | Woman | 59 | Malignant Rectal Tumor |
| 82 | 74 | Woman | 82 | Malignant Rectal Tumor |
| 83 | 78 | Man | 70 | Malignant Colonic Tumor |

| | | | | |
|---|---|---|---|---|
| 84 | 89 | Woman | 60 | Malignant Rectal Tumor |
| 85 | 77 | Man | 60 | Malignant Colonic Tumor |
| 86 | 79 | Woman | 65 | Malignant Rectal Tumor |
| 87 | 9 | Man | 49 | Malignant Rectal Tumor |
| 88 | 66 | Man | 69 | Malignant Rectal Tumor |
| 89 | 3 | Man | 76 | Malignant Rectal Tumor |
| 90 | 88 | Man | 51 | Malignant Rectal Tumor |
| 91 | 64 | Woman | 76 | Malignant Colonic Tumor |
| 92 | 81 | Woman | 60 | Malignant Colonic Tumor |

References


1. Lai, W., Ren, L., Tang, Q., Qu, X., Li, J., Wang, L., Li, L., Fan, C. and Pei, H. (2018) Programming Chemical Reaction Networks Using Intramolecular Conformational Motions of DNA. *ACS nano*, **12**, 7093-7099.
2. Genot, A.J., Zhang, D.Y., Bath, J. and Turberfield, A.J. (2011) Remote toehold: a mechanism for flexible control of DNA hybridization kinetics. *J. Am. Chem. Soc.*, **133**, 2177-2182.
3. Grosberg, A. (1998) *Theoretical and Mathematical Models in Polymer Research*. Academic Press, Boston.
4. Zhang, D.Y. and Winfree, E. (2009) Control of DNA strand displacement kinetics using toehold exchange. *J. Am. Chem. Soc.*, **131**, 17303-17314.
5. Murphy, M.C., Rasnik, I., Cheng, W., Lohman, T.M. and Ha, T. (2004) Probing single-stranded DNA conformational flexibility using fluorescence spectroscopy. *Biophys J.*, **86**, 2530-2537.
6. Srinivas, N., Ouldridge, T.E., Sulc, P., Schaeffer, J.M., Yurke, B., Louis, A.A., Doye, J.P. and Winfree, E. (2013) On the biophysics and kinetics of toehold-mediated DNA strand displacement. *Nucleic Acids Res.*, **41**, 10641-10658.
7. Hiatt, J.B., Pritchard, C.C., Salipante, S.J., O'Roak, B.J. and Shendure, J. (2013) Single molecule molecular inversion probes for targeted, high-accuracy detection of low-frequency variation. *Genome Res*, **23**, 843-854.
8. Didelot, A., Le Corre, D., Luscan, A., Cazes, A., Pallier, K., Emile, J.F., Laurent-Puig, P. and Blons, H. (2012) Competitive allele specific TaqMan PCR for KRAS, BRAF and EGFR mutation detection in clinical formalin fixed paraffin embedded samples. *Exp. Mol. Pathol.*, **92**, 275-280.
9. Newman, A.M., Bratman, S.V., To, J., Wynne, J.F., Eclov, N.C., Modlin, L.A., Liu, C.L., Neal, J.W., Wakelee, H.A., Merritt, R.E. *et al.* (2014) An ultrasensitive method for quantitating circulating tumor DNA with broad patient coverage. *Nat. Med.*, **20**, 548-554.
10. Olmedillas-López, S., Olivera-Salazar, R., García-Arranz, M. and García-Olmo, D. (2022) Current and Emerging Applications of Droplet Digital PCR in Oncology: An Updated Review. *Mol. Diagn. Ther.*, **26**, 61-87.
11. Abascal, F., Harvey, L.M.R., Mitchell, E., Lawson, A.R.J., Lensing, S.V., Ellis, P., Russell, A.J.C., Alcantara, R.E., Baez-Ortega, A., Wang, Y. *et al.* (2021) Somatic mutation landscapes at single-molecule resolution. *Nature*, **593**, 405-410.
12. Zhang, L., Wang, Y., Guo, Y., Chen, H., Yu, W., Zhang, Z. and Xie, G. (2021) A comprehensive system for detecting rare single nucleotide variants based on competitive

DNA probe and duplex-specific nuclease. *Analytica chimica acta*, **1166**, 338545.

13. Wu, L.R., Chen, S.X., Wu, Y., Patel, A.A. and Zhang, D.Y. (2017) Multiplexed enrichment of rare DNA variants via sequence-selective and temperature-robust amplification. *Nat. Biomed. Eng.*, **1**, 714-723.
14. Huang, D., Deng, H., Zhou, J., Wang, G.A., Lei, Q., Guo, C., Peng, W., Liang, P., Shen, C., Ying, B. *et al.* (2023) Mismatch-Guided Deoxyribonucleic Acid Assembly Enables Ultrasensitive and Multiplex Detection of Low-Allele-Fraction Variants in Clinical Samples. *J. Am. Chem. Soc.*, **145**, 20412-20421.
